# Probe‐Free, Primer‐Programmed Amplicon Encoding Enables Nanopore‐Based Multiplex Detection With Deep Learning Decoding

**DOI:** 10.1002/advs.78381

**Published:** 2026-10-22

**Authors:** Md. Ahasan Ahamed, Muhammad Asad Ullah Khalid, Wahid Uz Zaman, Anthony J. Politza, Weihua Guan

**Affiliations:** ^1^ Department of Intelligent Systems Engineering Indiana University Bloomington Indiana USA; ^2^ Department of Electrical Engineering Pennsylvania State University University Park Pennsylvania USA; ^3^ Department of Computer Science and Engineering Pennsylvania State University University Park Pennsylvania USA; ^4^ Department of Biomedical Engineering Pennsylvania State University University Park Pennsylvania USA

**Keywords:** deep learning, molecular diagnostics, multiplex nucleic acid testing, primer‐programmed amplicon encoding, recombinase polymerase amplification, solid‐state nanopore sensing

## Abstract

Single‐reaction multiplex nucleic acid testing (NATs) must both co‐amplify multiple targets and distinguish the resulting amplicons within the product mixture. Most conventional assays achieve this distinction using target‐specific reporters or other external encoding elements. Here, we introduce a primer‐programmed multiplexing strategy in which primer design generates target‐associated amplicons with predefined lengths that are read electrically with nanopores. In a proof‐of‐concept implementation, one‐pot recombinase polymerase amplification (RPA) generated four amplicons with predefined product lengths from Monkeypox (Mpox), Cowpox, Vaccinia, and human RNase P. The resulting amplicons were analyzed with glass nanopores, and individual translocation events were classified by a deep neural network (DNN) based on a six‐dimensional feature set derived from nanopore recordings. Event‐level decoding achieved >95% accuracy, >94.7% sensitivity, and >98.3% specificity, and aggregation of classified events yielded sample‐level calls with >93.3% sensitivity and >93.9% specificity in blinded samples, with qualitative calls matching polymerase chain reaction (PCR) results across the 9 PCR‐benchmarked samples. These results demonstrate a probe‐free multiplexing architecture in which target identity is programmed through primer‐defined amplicon design and decoded from the resulting multidimensional nanopore response using a DNN, eliminating the need for target‐specific reporter chemistry and opening new opportunities for all‐electrical multiplex nucleic acid testing based on unmodified amplification products.

## Graphical abstract

Primer‐programmed amplicon encoding enables reporter‐probe‐free multiplex nucleic acid identification using glass nanopore sensing and deep neural network decoding.

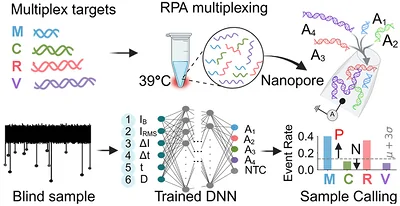

## 1 Introduction

Multiplexed NATs are central to infectious disease diagnosis. Many clinical syndromes can arise from pathogens with overlapping clinical presentations [1]. Respiratory infections [2, 3, 4], febrile illness [5, 6, 7], and rash syndromes [8] are common examples. Co‐infections further complicate diagnosis and clinical management [9]. Panel‐based molecular tests, therefore, provide clinically useful information from a single specimen [4]. Rapid multiplex detection can guide treatment decisions, reduce unnecessary antimicrobial use [10], and support outbreak surveillance [11]. Multiplexing is commonly implemented in two formats: multiple singleplex assays are run in parallel, or multiple targets are amplified within a single reaction. Parallel singleplex testing preserves assay simplicity but increases sample consumption, reagent use, and workflow burden [12]. In contrast, single‐reaction multiplex amplification allows pathogen targets and internal controls to be co‐amplified under shared conditions [13, 14, 15]. This integration reduces handling steps and improves assay efficiency [16]. As a result, single‐reaction multiplex assays are increasingly used in syndromic diagnostics.

Despite these practical advantages, most multiplex NATs assign target identity through extrinsic encoding [17]. In quantitative PCR and digital PCR, sequence‐specific probes carry distinct fluorophores [18], and targets are distinguished through spectrally separated detection channels [15]. Other approaches extend multiplex capacity through fluorescence lifetime encoding [19], temperature‐dependent signatures [20], or spatial barcoding on arrays [21] and bead libraries [22, 23]. Although effective, these strategies share a common feature: target identity is encoded by external reporters rather than by the amplification product itself. Practical multiplex performance is therefore shaped by the number of distinguishable reporters, spectral crosstalk, calibration requirements, and the availability of distinct optical detection channels [15, 24, 25]. Increasing the multiplex level also introduces biochemical constraints within the amplification reaction. As the number of primers and probes increases, the number of possible oligonucleotide interactions also increases, which can introduce amplification bias [26, 27]. These side reactions consume reagents and reduce amplification efficiency [27]. Competition among targets can compress dynamic range and create target‐dependent sensitivity. These effects complicate assay optimization and make multiplex assay design increasingly difficult. Collectively, these limitations motivate reconsideration of how multiplex decoding can be implemented. Nucleic acid amplification generates discrete DNA molecules with defined sequence composition and length [28], and these unmodified molecular properties could provide target‐associated information directly. If amplification products can be measured at the single‐molecule level, the amplicon itself could serve as the identifier. In this case, multiplex decoding would then rely on molecular features rather than on designed reporter chemistry.

Solid‐state nanopores provide a direct electrical interface for measuring properties of DNA molecules [29, 30, 31, 32]. Under an applied electric field, individual DNA strands translocate through a nanometer‐scale pore and generate transient ionic current blockades. The amplitude, duration, and waveform of each event reflect molecular size and conformation [33, 34]. Nanopore sensors can therefore detect DNA fragments at the single‐molecule level and distinguish molecules with different lengths [35]. Prior studies have also used nanopores for molecular identification and multiplex readout through designed DNA carriers [36], DNA nanoarray barcodes [37], site‐specific protein barcodes [38], and related engineered barcode architectures. These studies demonstrate the capability of nanopores to decode molecular identity, but the target‐identifying information is typically introduced through external carrier structures, affinity‐mediated binding, conjugation chemistry, barcode‐forming reagents, or engineered molecular labels rather than being inherent to the amplification product itself. Such strategies may also require additional assembly or binding steps, and incomplete target‐carrier association can reduce the fraction of informative sensing events. By contrast, primer‐programmed unmodified amplicon encoding does not require target‐specific reporter probes, engineered carriers, affinity labels, or post‐amplification barcode‐forming chemistry for target identification. Target identity is encoded at the primer‐design stage through the length of the amplification product itself, which is also the nanopore‐sensed identifier.

A complementary approach is therefore to use the unmodified amplification product itself as the identifier, so that the same molecule generated by amplification also provides the physical feature used for decoding. This distinction is important because probe‐free multiplex amplicon decoding presents a more complex measurement problem than reading purified engineered barcodes or well‐defined analytes. Recent studies have coupled nanopores with nucleic acid amplification to electronically detect amplification products [30, 32, 39]. Computational analysis using machine learning (ML) can further improve the interpretation of nanopore signals by integrating multidimensional signal features [34, 40, 41]. This is useful because conventional threshold‐based analysis often depends on manually selected cutoffs for individual descriptors, such as blockade amplitude, dwell time, or event charge deficit. These cutoffs can be sensitive to pore geometry, event frequency, baseline noise, and experimental conditions. In contrast, a trained classifier can combine multiple event descriptors within a consistent decision framework, reducing reliance on manually chosen single‐parameter thresholds. Probe‐free multiplex decoding nevertheless remains challenging [42]. Single‐reaction multiplex amplification can produce complex mixtures containing both intended amplicons and nonspecific products [27]. Nanopore signals may also vary across pores and fluctuate over time [43, 44]. These sources of heterogeneity limit simple threshold‐based decoding. An analytical framework must therefore convert large numbers of stochastic single‐molecule events into reliable sample‐level calls anchored to appropriate negative controls.

Here, we present a probe‐free multiplex decoding framework based on primer‐programmed unmodified amplicon encoding and nanopore readout. As a proof‐of‐concept model implementation of this strategy, we used single‐reaction RPA and designed primer sets that generate amplicons with separable lengths. Unmodified amplicon length generated by pre‐programmed primers thus serves as an encoding dimension. Mixed amplification products were analyzed using glass nanopores, and individual translocation events were classified using multidimensional nanopore signal features with a DNN model. Event‐level predictions were then aggregated into sample‐level calls using thresholds defined relative to the no‐template control (NTC). Using this framework, we demonstrate four‐target multiplex detection within a single reaction and reproducible sample‐level decoding across independent nanopores. These results demonstrate a proof‐of‐concept for primer‐programmed amplicon encoding as a strategy for probe‐free multiplex NATs with all‐electrical nanopore readout and provide a basis for evaluating the encoding principle with additional amplification targets.

## 2 Results and Discussion

### 2.1 Framework for Primer‐Programmed Multiplex Amplicon Decoding and Sample Calling

To translate primer‐programmed unmodified amplicon encoding into a reliable multiplex readout, we established a nanopore‐based decoding framework in which nanopore translocation signals are first recorded from individual DNA molecules and then decoded by a DNN to assign each event to one of four target‐associated amplicon classes or a non‐target (NT) class, thereby converting single‐molecule measurements into sample‐level positive or negative calls. In this framework, probe‐free multiplexing is achieved by designing RPA primer sets that produce amplicons with distinct lengths, such that target identity is encoded directly in the amplification products. Figure 1A outlines the overall workflow. A sample containing four representative targets (M, C, R, and V) is amplified in a single RPA reaction to generate length‐defined amplicons. These products are then analyzed using sub‐10 nm glass nanopores, where individual DNA molecules translocate through the pore under an applied electric field and produce transient ionic current blockades recorded in current–time (*I*–*t*) traces.

**FIGURE 1:**
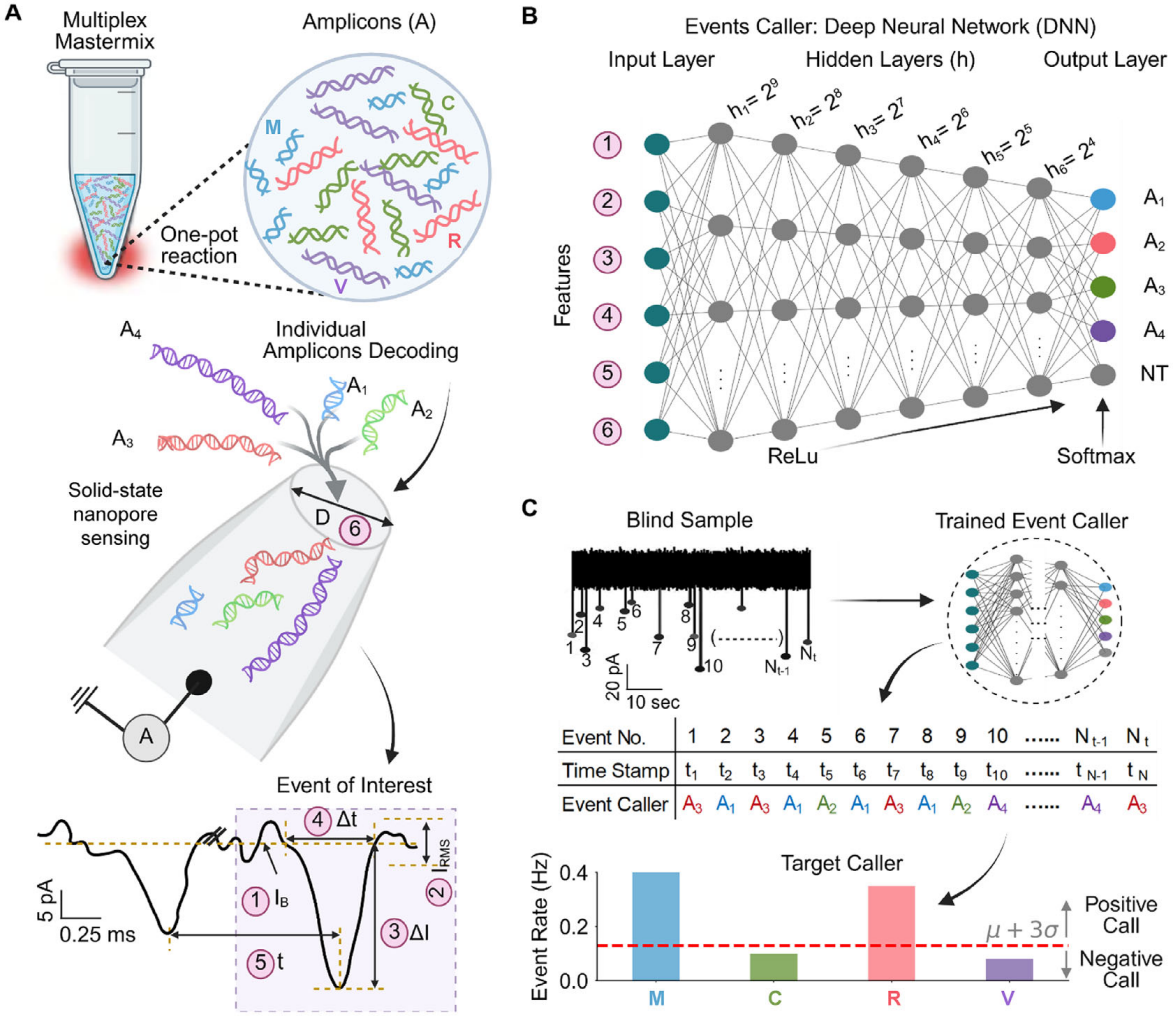
Probe‐free decoding of primer‐programmed amplicons by nanopore sensing and DNN‐based classification. (A) Schematic of the multiplex amplification and nanopore sensing workflow. Four targets (M, C, R, and V) are amplified in a single reaction to generate amplicons with distinct lengths (A_1_, A_2_, A_3_, and A_4_). The resulting amplicons translocate through a glass nanopore and generate ionic current signals recorded as *I*–*t* traces. Six quantitative features are extracted from each event: baseline current, RMS current, blockade amplitude, dwell time, time since the previous event, and nanopore diameter. (B) Event caller: A DNN model is trained on these six features to assign individual events to one of five classes: A_1_, A_2_, A_3_, A_4_, or NT. Feature vectors propagate through multiple nonlinear hidden layers to produce predicted classes. (C) Target caller: Event classifications from blinded samples are aggregated to calculate target‐specific event rates over the acquisition period. A target is called positive when the event rate exceeds a statistical threshold defined as µ + 3σ of the event‐rate distribution measured across NTC replicates.

To prepare raw nanopore recordings for DNN‐based decoding, each detected translocation event was converted into a quantitative feature vector. Six event‐level features were extracted: baseline current (I_B_), root‐mean‐square (RMS) current (I_RMS_), blockade amplitude (ΔI), dwell time (Δt), time since the previous event (t), and nanopore diameter (D). I_B_ and I_RMS_ describe the local baseline and noise environment, whereas ΔI and Δt describe the individual current‐blockade event; t captures the temporal spacing between consecutive events, and D accounts for pore‐dependent signal scaling. Representative feature extraction and event‐level values are shown in Figure S1. Primer design defines target‐associated amplicons with distinct product lengths, while the six extracted features characterize their multidimensional nanopore responses. Because the four amplicons also differ in sequence composition, the present study does not isolate sequence‐independent length discrimination. The DNN therefore decodes the composite electrical response of the primer‐programmed amplicons, with local baseline, noise, temporal spacing, and pore size providing contextual information needed to accommodate temporal and sensor‐to‐sensor variability.

We asked whether primer‐programmed RPA products could be reliably distinguished using conventional single‐parameter or two‐parameter nanopore descriptors alone. We examined blockade amplitude‐dwell time (ΔI‐Δt) distributions and event charge deficit (ECD) values for the four singleplex target classes. As shown in Figure S2, the ΔI–Δt distributions for M, C, R, and V showed substantial overlap. Adjacent‐target ECD comparisons using two‐sided Welch's t‐tests yielded *p* = 0.318 (M vs. C), *p* = 6.5 × 10^−6^ (C vs. R), and *p* = 0.056 (R vs. V), with two of the three comparisons not statistically significant. These results indicate that conventional low‐dimensional nanopore descriptors are insufficient for robust multiplex decoding.

We therefore asked whether combining multiple event‐level and pore‐related features could improve discrimination. To capture multidimensional and potentially nonlinear relationships among these features, we trained a DNN to classify individual nanopore events using the six features. The model assigns each event to one of five event classes: four target‐associated amplicon classes (A_1_‐A_4_) or a non‐target (NT) class (Figure 1B). The NT class was included to represent events that do not match the four target‐associated amplicon classes, including background species, nonspecific products, and RPA‐associated artifacts. This design allowed the classifier to use multiple signal descriptors simultaneously. To visualize whether the six‐feature representation contained class‐associated information beyond conventional nanopore descriptors, we analyzed representative 3‐plex and 4‐plex datasets using t‐distributed stochastic neighbor embedding (t‐SNE). As shown in Figure S3, t‐SNE projections of the standardized raw six‐feature input vectors showed partial class‐associated structure, but the target classes remained closely positioned and partially overlapping, indicating that the raw feature space was not fully separable. In contrast, t‐SNE projections of the penultimate‐layer DNN representation showed clearer class organization after nonlinear transformation by the trained network. These results support the use of DNN‐based multidimensional feature learning for event classification, although t‐SNE was used only for qualitative visualization and was not used for sample calling.

To convert event‐level DNN predictions into sample‐level positive or negative calls, classified events were aggregated over the full acquisition period. For each sample, the total measurement time *T* was defined as the sum of the inter‐event intervals *t*_1_ to *t*_N_ (Figure 1C), and target‐specific event rates were calculated over the corresponding acquisition period. A target was called positive when its DNN‐assigned event rate exceeded the pre‐established µ + 3σ = 0.14 Hz NTC threshold, where µ = 0.066 Hz and σ = 0.024 Hz. The NTC background distribution was characterized using 11 independent measurements, and the event‐rate distribution showed no significant departure from normality by the Shapiro‐Wilk test (*p* = 0.21) (Figure S4A–D). The resulting threshold was then tested, with µ and σ held fixed, on 3 additional NTCs measured on nanopores not used for the 11 baseline NTC measurements. These independent NTCs produced event rates of 0.050, 0.032, and 0.046 Hz, all below the fixed 0.14 Hz decision boundary, and were therefore correctly classified as negative (Figure S4E,F). Importantly, the DNN was fully trained and fixed before threshold estimation. The µ + 3σ criterion was therefore applied only as a downstream sample‐level decision boundary and did not participate in event‐level model training or target assignment. Under these conditions, target‐associated amplicons typically produced event rates above this threshold, whereas background species and inherent RPA artifacts produced sparse, low‐frequency events that remained below threshold and were often characterized by longer dwell times (Figure S5A). Typical amplicon translocation events exhibit dwell times of approximately 0.2–0.8 ms with blockade amplitudes ranging from 25 to 200 pA in an amplicon‐ and pore‐dependent manner; representative events are shown in Figure S5B–E. Representative waveform comparisons further showed that NT and background events were heterogeneous, including shallow long‐duration events, high‐amplitude long‐duration events, and irregular broad waveforms, whereas target amplicons showed more reproducible class‐dependent event morphologies. These results establish a probe‐free electrical readout approach based on primer‐programmed amplicon encoding, multidimensional event classification, and NTC‐referenced sample calling.

### 2.2 Design and Validation of the Primer‐Programmed Multiplex Assay

To implement primer‐programmed amplicon encoding for multiplex detection, amplification reactions must generate discrete target‐associated amplicons with predefined product lengths that support downstream nanopore discrimination. We therefore designed and validated an RPA reaction, which supports rapid isothermal amplification and enables multiplex reactions in a single pot while producing defined amplicon lengths suitable for downstream nanopore discrimination [45, 46]. RPA was selected not because it is intrinsically superior to PCR or other amplification methods, but because its isothermal operation and primer‐defined amplicon products are compatible with the present primer‐programmed amplicon strategy and direct nanopore readout. In contrast, amplification chemistries that generate heterogeneous or concatemeric products are less directly suited for decoding. The assay was configured to distinguish four targets through their corresponding amplicon lengths. The assay targets Mpox (M; 110 bp), Cowpox (C; 241 bp), human RNase P gene (R; 310 bp), and Vaccinia (V; 449 bp), as summarized in Figure 2A. The detailed target sequences are shown in Table S1. Primer pairs were designed to generate target‐associated amplicons with distinct predefined lengths, providing a controlled assay‐design parameter for downstream nanopore decoding, as shown in Figure 2B. We next screened predicted primer‐interaction free energies (>−9 kcal mol^−1^) to reduce potential cross‐reactivity (Figure S6) [47]. To validate the multiplex RPA assay and further confirm primer specificity, we performed polymerase chain reaction (PCR) followed by gel electrophoresis (Figure S7), which showed no detectable cross‐amplification. On this basis, we selected the final primer panel for RPA multiplex amplification.

**FIGURE 2:**
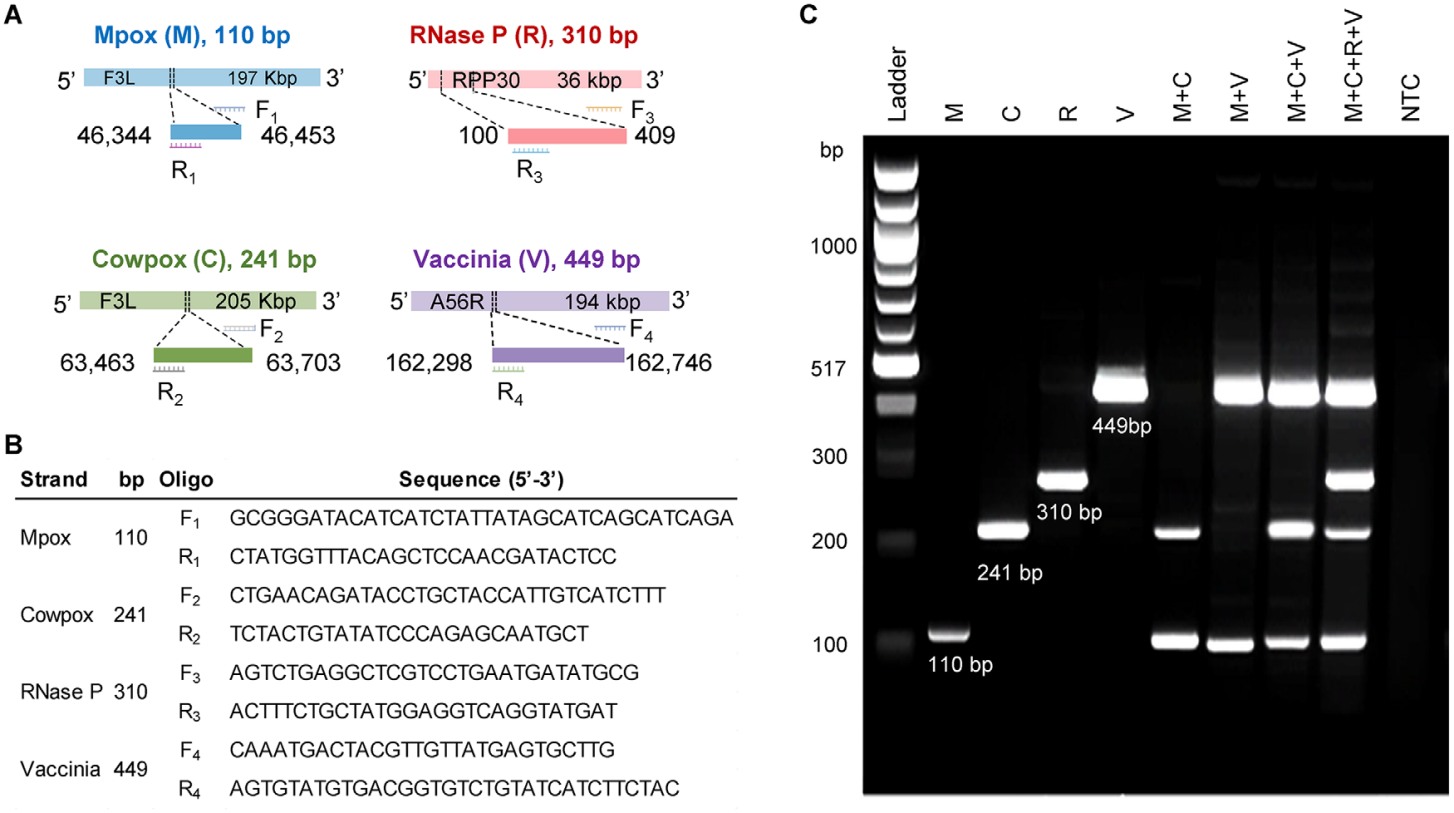
Design and validation of the primer‐programmed multiplex RPA assay. (A) Schematic of four targets used for primer‐programmed amplicon encoding: Mpox (M), 110 bp; Cowpox (C), 241 bp; human RNase P (R), 310 bp; Vaccinia (V), 449 bp. The targets include lesion‐associated poxviruses, M, C, and V, with R serving as a process control. Primer pairs F_1_/R_1_, F_2_/R_2_, F_3_/R_3_, and F_4_/R_4_ define the expected amplicon lengths for multiplex identification. (B) Oligonucleotide sequences used in this study. Forward and reverse primers are listed 5′ to 3′ together with strand orientation and expected amplicon length for each target. (C) Agarose gel validation of multiplex RPA amplification. Lane 1, ladder; lanes 2–5, singleplex M, C, R, and V; lanes 6–7, duplex M+C and M+V; lane 8, triplex M+C+V; lane 9, four‐plex M+C+R+V; lane 10, NTC. Bands at 110, 241, 310, and 449 bp correspond to the designed amplicon lengths. Conditions: 3% agarose, GelRed stain, RPA 20 min at 39°C, electrophoresis at 6 V cm^−1^.

To evaluate the performance of the multiplex assay, we first examined RPA products by gel electrophoresis (Figure 2C). Singleplex reactions produced clear, target‐specific bands for M, C, R, and V at the expected sizes. Representative duplex reactions (M+C and M+V), a representative triplex reaction (M+C+V), and the four‐plex reaction (M+C+R+V) all showed band patterns consistent with the corresponding combinations, confirming that the multiplex assay supports simultaneous amplification of multiple targets and that the results are consistent with the PCR validation results in Figure S7. Accordingly, the present assay demonstrates four‐target multiplexing within the tested 110–449 bp coding range, but it was not designed to define the minimum resolvable amplicon‐length spacing. The slight apparent shifts in the bands observed in some multiplex lanes are attributed to minor lane‐to‐lane electrophoretic variation and sample‐composition effects rather than changes in amplicon identity. In multiplex reactions, differences in total DNA amount, residual salts, amplification components, and relative amplicon abundance can slightly affect DNA migration through the agarose matrix and alter the apparent band center during imaging. Importantly, these bands remained within the expected size ranges and were consistent with the corresponding singleplex controls and DNA ladder. Faint bands and smearing are also present in the NTC, indicating low‐level nonspecific RPA background [48]. Such background products are common in RPA reactions and typically consist of short nonspecific fragments and primer‐derived species. In the nanopore workflow, these background molecules contribute to sparse low‐frequency events that remain below the NTC‐derived detection threshold and are further rejected by DNN‐based event classification, thereby minimizing their impact on target identification (Figure S4A–D). These results support the feasibility of the primer‐programmed multiplex RPA assay for downstream nanopore analysis. Detailed singleplex and multiplex reaction compositions are provided in Tables S2–S5. The present study was designed for qualitative multiplex target identification rather than as a quantitative diagnostic benchmarking study. Representative singleplex, duplex, triplex, and four‐plex compositions were therefore selected to assess multiplex amplification and downstream target calling across increasing assay complexity rather than to exhaustively evaluate the full concentration‐composition space. Endpoint gel electrophoresis revealed differences in product yield among singleplex and multiplex reactions, but this endpoint measurement cannot directly quantify target‐specific amplification efficiency. Rigorous determination of amplification efficiency would require concentration‐dependent kinetic measurements with target‐resolved quantitative readout, typically using target‐specific fluorescent probes. Such measurements are beyond the scope of the present probe‐free proof‐of‐concept assay. For qualitative target identification, equal amplification efficiency across targets is not required. Comprehensive quantitative validation will further require systematic assessment of target‐specific limits of detection, dynamic range, concentration‐dependent performance, matrix effects, assay variability, turnaround time, and workflow integration. These parameters remain subjects for future validation.

### 2.3 Optimization of Nanopore Sensor Working Window

To test a nanopore operating window suitable for multiplexed RPA amplicon detection, we characterized DNA fragments spanning 50–500 bp, covering the expected RPA amplicon size range of approximately 80–500 bp [49]. The designed multiplex amplicons span approximately 100–500 bp, providing distinct predefined amplicon lengths for multiplex identification. To establish a lower‐fragment‐length detectability test for the nanopore platform, we examined the translocation behavior of the lower‐bound amplicon size (75 bp DNA fragment). This lower‐bound test assesses whether the nanopore sensor can detect DNA fragments shorter than the smallest designed amplicons. Measurements were performed using a ∼6 nm nanopore in 1 M KCl (Figure S8). Electrical characterization shows a linear current–voltage (*I*–*V*) response and stable baseline currents across applied biases from +100 to +400 mV (Figure S8A,B). Representative *I*–*t* traces reveal clear translocation events whose frequency increases with applied bias, while statistical analysis shows a linear voltage dependence of event rate, exponentially distributed interarrival times, as expected for a Poisson arrival process, and Gaussian blockade amplitude distributions (Figure S8C–F), indicating reproducible signal signatures for short DNA fragments. These results demonstrate that a 75 bp DNA fragment generates detectable nanopore translocation events above the electrical noise floor under the tested conditions, establishing detectability below the size of the smallest designed RPA amplicon.

Because nanopore diameter strongly affects capture efficiency, translocation dynamics, and blockade amplitude, we next systematically evaluated the relationship between pore size and DNA fragment length. We estimated nanopore diameter by ionic conductance using the conical‐pore conductance model described in Equation S1 in Section S1. We extracted conductance values from linear fits to current–voltage (*I*–*V*) curves measured in the ohmic regime. Representative *I*–*V* measurements for 9 independent pores showed high linearity, with R^2^ values ≥ 0.99, validating the use of conductance‐based diameter estimation (Figure S9). Replicate pores within the three evaluated nominal diameter groups showed low between‐pore variation in conductance‐derived diameter, with coefficients of variation of 5.1%–8.9% (Figure S10). We used these three replicate groups to assess the reproducibility of laser‐pulled nanopore fabrication across representative pore‐size regimes. Together, these measurements show that nanopore diameter can be reproducibly characterized across independent pores.

The *I*–*t* traces captured by nanopore sensors reveal three distinct regimes in the sensing process: a clogging regime, a less sensitive regime, and an optimal sensing regime, as illustrated in Figure 3A. The clogging probability increases if the radius of gyration (*r*) of the DNA is much larger than the nanopore diameter. The radius of gyration follows the relation *r^2^* = *LP*/3, where *L* = DNA contour length (*N* × 0.34 nm), *N* = fragment size (bp), and *P* is the persistence length [50, 51]. Translocation typically occurs through a stochastic threading process [52]. To summarize the physical phenomena that emerge as both nanopore diameter and DNA fragment length vary, Figure S11 summarizes the resulting transport behaviors and sensing regimes across the tested size combinations. For longer DNA fragments, such as 300 and 500 bp, with corresponding *r* values of 41.2 and 53.2 nm, the probability of clogging increases markedly when small nanopores (<6.3 nm) are used. Fragments shorter than approximately 300 bp behave as rod‐like molecules, whereas longer fragments adopt coiled conformations, increasing the probability that coiled molecules obstruct the pore entrance during capture before successful threading occurs. In contrast, for short rod‐like fragments such as 75 and 100 bp, large pores (>9.1 nm) produce very high translocation velocities and shallow blockades, causing many events to fall below the detection threshold and reducing sensing sensitivity. Figure 3B shows that nanopores with sub‐10 nm diameters define the most favorable operating regime for RPA amplicons under the tested conditions. This size range supports efficient threading and high event rates for DNA fragments from 75 to 500 bp without substantial clogging. In the corresponding heat map, green indicates regions where events are detected, red indicates regions where events are undetected, and black indicates regions associated with pore clogging. The dashed box highlights the assay operating region at nanopore diameters of approximately 6.3–9.1 nm and DNA fragment lengths of 100–500 bp. The 50‐ and 75‐bp fragments were evaluated separately as a lower‐bound detection test. Within this window, increasing nanopore diameter initially raises the event frequency because DNA molecules are captured and transported more readily. When the diameter exceeds this range, however, the event frequency decreases, as weaker, less specific interactions between DNA and the pore reduce the likelihood of efficient capture and translocation. These results identify an assay‐specific operating window of 6.3–9.1 nm for stable signal acquisition across multiple nanopores.

**FIGURE 3:**
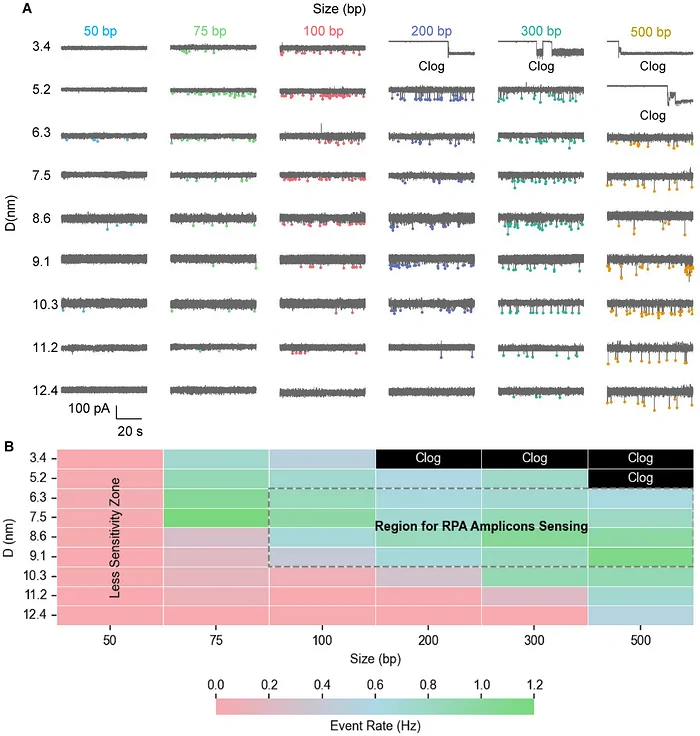
Size‐dependent nanopore response across DNA fragment sizes and pore diameters. (A) Representative *I*–*t* traces for 1 nM DNA fragments of 50, 75, 100, 200, 300, and 500 bp acquired with nanopores of 3.4–12.4 nm diameter at 400 mV. Scale bars: 100 pA and 20 s. (B) Heat map of mean event rate as a function of fragment size and pore diameter. Color encodes event rate (Hz); light red denotes no detectable events, and black denotes conditions in which pore clogging prevented event acquisition. The dashed rectangle marks the operating window used for RPA amplicon sensing, spanning fragments of 100–500 bp and pore diameters of approximately 6.3–9.1 nm.

### 2.4 Deep Neural Network Decoding of Nanopore Translocation Events

To classify nanopore events generated from primer‐programmed multiplex RPA in the presence of background signals and pore‐to‐pore variability, we developed a multidimensional machine‐learning framework that maps event‐associated signal features to target identity. To first establish measurement conditions compatible with amplified products, NTC, M, R, and M+R duplex samples were evaluated using a 6.3 nm nanopore across different RPA‐product dilutions. Detectable events were retained across the tested conditions, while event rates decreased approximately linearly with increasing dilution (Figure S12). A 20× dilution was therefore selected to reduce matrix‐associated interference and clogging while maintaining sufficient event frequency for classification.

To determine the most informative feature representation for DNN‐based event classification, we evaluated eight candidate features derived from nanopore translocation signals and experimental parameters, including baseline current (I_B_), RMS current (I_RMS_), blockage amplitude (ΔI), dwell time (Δt), inter‐peak interval (t), nanopore diameter (D), voltage (V), and salt conductivity (σ). These variables capture molecular translocation characteristics and experimental conditions that influence nanopore signals. Feature selection identified I_B_, I_RMS_, ΔI, Δt, t, and D as the most informative features for classification, yielding the highest model accuracy (Figure 4A). To evaluate the stability of this feature representation, each feature subset was retrained across eight runs using different random seeds, and performance is reported as mean ± standard deviation rather than from a single run. The selected six‐feature set showed the highest mean accuracy across these repeated evaluations and was therefore retained for subsequent model development. To further assess the contribution of individual inputs, we performed one‐feature‐at‐a‐time ablation of the selected six‐feature representation. Removing RMS current, pore diameter, or blockade amplitude produced the largest decreases in classification accuracy, whereas removal of baseline current, inter‐event interval, or dwell time had smaller effects (Figure S13A). These results indicate that robust decoding depends on both amplicon‐associated translocation information and sensor‐context features.

**FIGURE 4:**
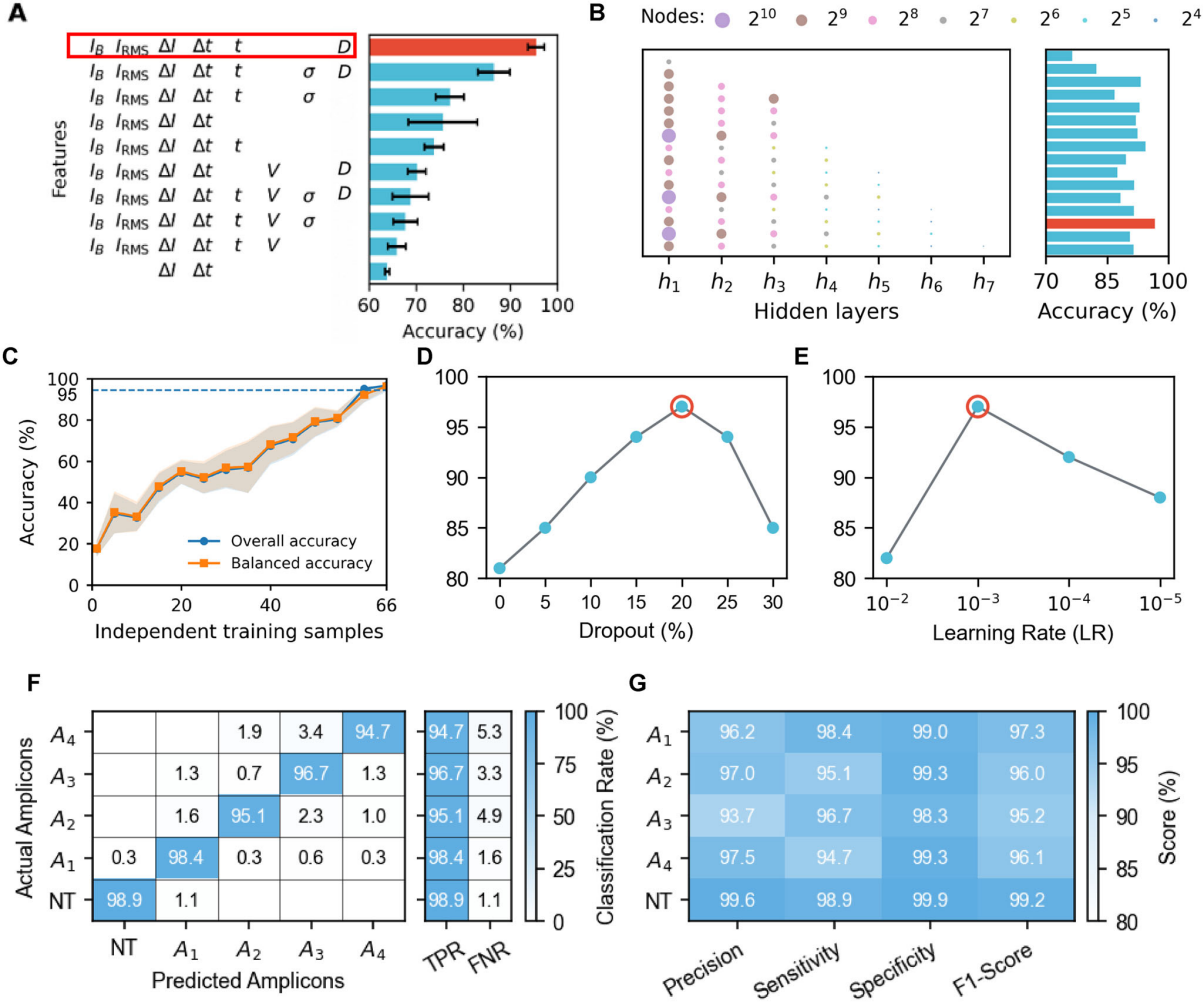
Feature selection, model comparison, DNN architecture exploration, and training optimization for event‐level amplicon classification. (A) Feature‐selection analysis across different feature subsets. Accuracies are reported as mean ± standard deviation over eight runs using different random seeds. The six‐feature subset I_B_, I_RMS_, ΔI, Δt, t, and d (red bar) showed the highest mean accuracy and was selected for subsequent model development. (B) Network‐architecture optimization across different hidden‐layer node configurations; the architecture with hidden layers of 512, 256, 128, 64, 32, and 16 nodes gave the highest classification accuracy. (C) Independent‐sample learning curve showing overall and balanced DNN accuracy across increasing numbers of training samples. Shaded regions indicate variability in model performance, and the dashed horizontal line denotes 95% accuracy. Overall and balanced accuracy reached 96.7% and 96.2%, respectively, with the complete 66‐sample development cohort (∼7500 event‐level observations). (D) Effect of dropout rate on model performance. (E) Effect of learning rate on model performance. (F) Confusion matrix for five‐class event‐level classification (A_1_‐A_4_ and NT). Most predictions fall on the diagonal, consistent with accurate classification in the held‐out test set. (G) Summary performance metrics including precision, sensitivity, specificity, and F1 score.

Using this fixed six‐feature representation, we next compared representative machine‐learning classifiers to determine whether the multidimensional signal space benefited from deep nonlinear mapping. Random forest, support vector machine, and logistic regression achieved accuracies of 81.43%, 80.50%, and 65.65%, respectively, whereas the DNN achieved 96.7% (Figure S13B). Based on this comparison, the DNN was selected for subsequent architecture and hyperparameter optimization. NT was included as an explicit event class together with A_1_–A_4_, enabling background and nonspecific events to be modeled within the same five‐class classification framework. The model‐development workflow is summarized in Figure S13C. Activation functions were evaluated separately using the fixed DNN framework, with ReLU providing the highest classification accuracy among the tested functions (Figure S13D).

The 66‐sample development cohort acquired across 21 nanopores was used for feature selection and model optimization, with representative current traces shown in Figure S14. Although the cohort size is reported in terms of independent nanopore samples, model training and evaluation were performed using event‐level data, comprising approximately 7500 individual events in total across the 66 samples. The standardized event‐level feature matrix was partitioned into training, validation, and internal‐test subsets for model development. Model weights were optimized only using the training subset through backpropagation with cross‐entropy loss, while the validation subset was used for model selection and early stopping. The internal test subset remained excluded from weight updating and was used only for performance assessment within the development cohort.

We then systematically evaluated 16 fully connected architectures spanning one to seven hidden layers and multiple node configurations. Classification accuracy ranged from 76.4% for a single 128‐node layer to 96.7% for the six‐layer 512–256–128–64–32–16 configuration (Figure 4B and Figure S15A). To distinguish event volume from independent experimental diversity, we evaluated model performance as a function of the number of whole independent samples used for training. Overall accuracy reached 95.1% with 61 samples and 96.7% with the complete 66‐sample development cohort, whereas balanced accuracy reached 92.3% and 96.2%, respectively (Figure 4C). These results show that performance continued to improve with increasing independent sample diversity, while the additional gain in overall accuracy became smaller near the upper end of the tested sample range. Key hyperparameters were subsequently tuned. A batch size of 64 yielded the best performance (Figure S15B), whereas a dropout rate of 20% most effectively controlled overfitting (Figure 4D). Learning‐rate optimization identified 10^−3^ as providing stable and efficient convergence (Figure 4E). A 60/20/20 training‐validation‐test partition was used for internal model development and evaluation (Figure S15C). The training subset was used for weight optimization, the validation subset for model selection and early stopping, and the internal test subset was withheld from weight updating and used only to assess performance within the development cohort. This internal event‐level partition was distinct from the subsequent evaluation performed on independently acquired nanopore measurements.

After the feature set, network architecture, and hyperparameters were fixed, the trained classifier was evaluated using a separate held‐out cohort of 23 samples measured on 16 different nanopores that were not represented in model training, comprising approximately 1508 event‐level observations in total. This independent‐pore evaluation was used to assess generalization beyond the nanopores included in the development cohort. To evaluate DNN performance for five‐class classification of A_1_‐A_4_ and NT, we generated an event‐count confusion matrix (Figure S15D) and calculated class‐specific performance metrics (Figures 4F,G). The event count matrix shows that most predictions fall on the diagonal: 307 of 312 A_1_ events, 291 of 306 A_2_ events, 296 of 306 A_3_ events, 306 of 323 A_4_ events, and 258 of 261 NT events are correctly classified. To quantify false target assignment from background events, we specifically evaluated the NT row of the confusion matrix. The event‐level false classification rate for NT was defined as the fraction of actual NT events predicted as A_1_, A_2_, A_3_, or A_4_. Among 261 held‐out NT events, 258 were correctly classified as NT, while 3 were assigned to target classes, corresponding to an NT false target‐assignment rate of 1.1%. This analysis directly evaluates nonspecific‐background misclassification within the same five‐class DNN framework used for target classification. The high NT sensitivity of 98.9% and the inclusion of NT in the per‐class precision, specificity, F1‐score, and area under the receiver operating characteristic curve (ROC‐AUC) analyses further indicate that background events were quantitatively evaluated rather than excluded by manual filtering. The corresponding percentage matrix reports true positive rates of 98.4% (A_1_), 95.1% (A_2_), 96.7% (A_3_), 94.7% (A_4_), and 98.9% (NT), with false negative rates below 6% for all classes (Figure 4F). Misclassifications are rare and occur mainly between neighboring amplicons, reflecting partial overlap in the feature space. Consistent with these patterns, precision, sensitivity, specificity, and F1 scores are uniformly high across classes, ranging from 93.7%–99.6%, 94.7%–98.9%, 98.9%–99.9%, and 95.2%–99.2%, respectively (Figure 4G). ROC analysis further demonstrates excellent class separability, with AUC values between 0.99 and 1.00 for all five classes (Figure S15E). As a complementary leave‐one‐pore‐out assessment in Figure S15F, we excluded all events from one physical nanopore from model‐weight training and used them only for evaluation, then retrained the classifier on events from the remaining training pores. Classification accuracy across the evaluable left‐out pores ranged from 81.6% to 95.9%. Figure S16 shows representative signals from this held‐out evaluation, highlighting the variability encountered across independent nanopores.

### 2.5 Integrated Analytical Workflow Benchmarking and Cross‐Pore Consistency

To evaluate the analytical workflow and reproducibility, we benchmarked the complete RPA–nanopore‐DNN workflow against PCR validation using 9 samples spanning singleplex through four‐plex target compositions (Figure 5A). These mixtures correspond to the PCR validation dataset shown in Figure S7 and include an NTC to assess false‐positive susceptibility. This benchmarking experiment evaluates the integrated analytical workflow under the tested laboratory conditions, from multiplex amplification to nanopore measurement and DNN‐based event decoding against an orthogonal reference method.

**FIGURE 5:**
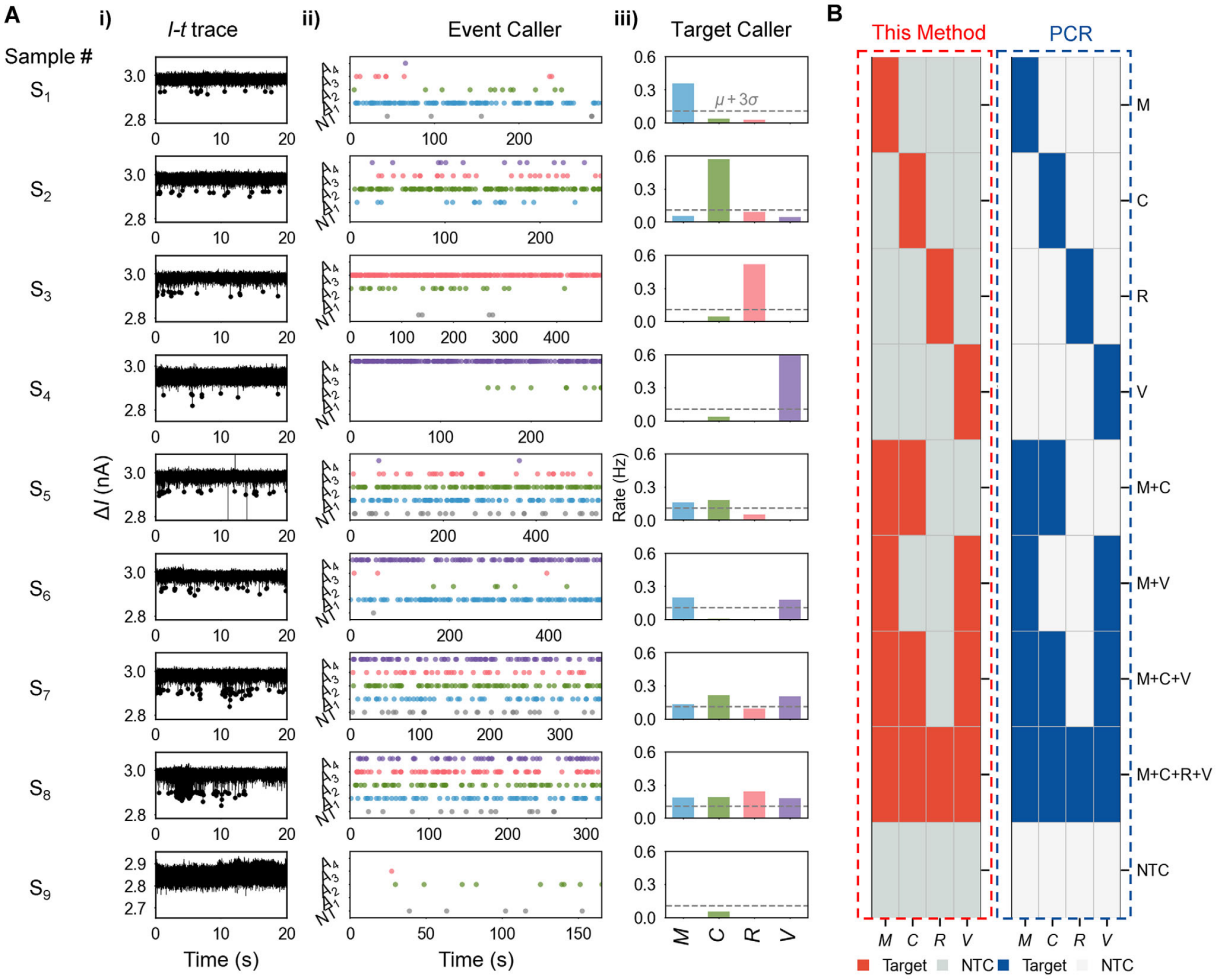
Benchmarking the RPA–nanopore–DNN multiplex workflow against PCR. (A) 9 samples (S_1_–S9) corresponding to the mixtures in Figure 2C were analyzed using the event caller and target caller. i) Representative *I*–*t* traces for each sample; ii) time‐stamped event‐level classifications for A_1_, A_2_, A_3_, A_4_, and NT; iii) target‐specific event‐rate histograms for each sample. The decision threshold is defined as µ + 3σ of the event‐rate distribution measured across NTC replicates. Target identities are reported as M, C, R, and V below each histogram. (B) Benchmarking matrix comparing ground‐truth target composition with qualitative calls generated by the nanopore‐DNN workflow. For the 9 benchmark mixtures tested, the nanopore‐DNN qualitative calls matched the PCR‐defined target compositions shown in Figure S7.

For each sample, raw *I*–*t* traces were processed by an event caller that extracts feature vectors for individual translocation events and streams these features into the trained DNN, enabling time‐stamped, event‐level assignments to the four amplicon classes (A_1_–A_4_) and NT. Event‐level classifications were then aggregated into target‐specific event rates to convert single‐molecule detections into interpretable sample‐level calls. A decision boundary, defined as µ + 3σ of event rates measured from multiple NTC replicates, was applied to distinguish positive from negative calls for each target channel. The resulting qualitative calls for M, C, R, and V were compiled into a benchmarking matrix and directly compared with PCR‐defined ground truth for the same mixtures (Figure 5B; Figure S7). For all 9 benchmark mixtures tested, the nanopore‐DNN qualitative calls matched the PCR‐defined target compositions. This experiment was used to compare the workflow with PCR across different mixture complexities and was not intended to provide a broader statistical performance estimate. Singleplex, duplex, triplex, and four‐plex samples were correctly resolved, while the NTC sample remained negative across all targets. Within the tested panel, this agreement indicates that event‐level classification can be translated into accurate sample‐level decisions across mixtures of increasing complexity.

To further evaluate whether multiplex sample calling is reproducible across independent nanopore sensors, we repeated representative reactions sequentially on three independent nanopores (P_1_, P_2_, and P_3_) under identical conditions (1 M KCl, 400 mV). We applied the same event‐to‐sample classification workflow in each case, from raw *I*–*t* trace to event classification, rate aggregation, and final qualitative calling using a fixed statistical threshold (µ + 3σ) derived from the event‐rate distribution measured across NTC replicates. In Figure 6A(i–iv), a singleplex target (M) yields a dominant stream of DNN‐assigned events and a corresponding event‐rate peak that consistently exceeds the threshold across P_1_, P_2_, and P_3_, while all non‐present targets remain below the decision threshold and are correctly called negative. In Figure 6B(i–iv), the duplex mixture (M+C) produces two target‐specific event streams and two target peaks that reproducibly cross the threshold across all nanopores. In Figure 6C(i–iv), the triplex mixture (M+C+V) shows sustained event‐level classifications and rate elevations for three targets, with the absent target remaining below threshold. In Figure 6D(i–iv), the four‐plex mixture (M+C+R+V) yields above‐threshold event‐rate signatures for all four targets on each pore, with fully concordant qualitative outputs across P_1_, P_2_, and P_3_ despite intrinsic pore‐to‐pore variability. Because each event included locally extracted I_B_ and I_RMS_ together with ΔI and Δt (Figure S1), pore‐dependent shifts in baseline current, noise level, blockade amplitude, and dwell time were represented in the event‐level feature matrix rather than treated as unmodeled variation.

**FIGURE 6:**
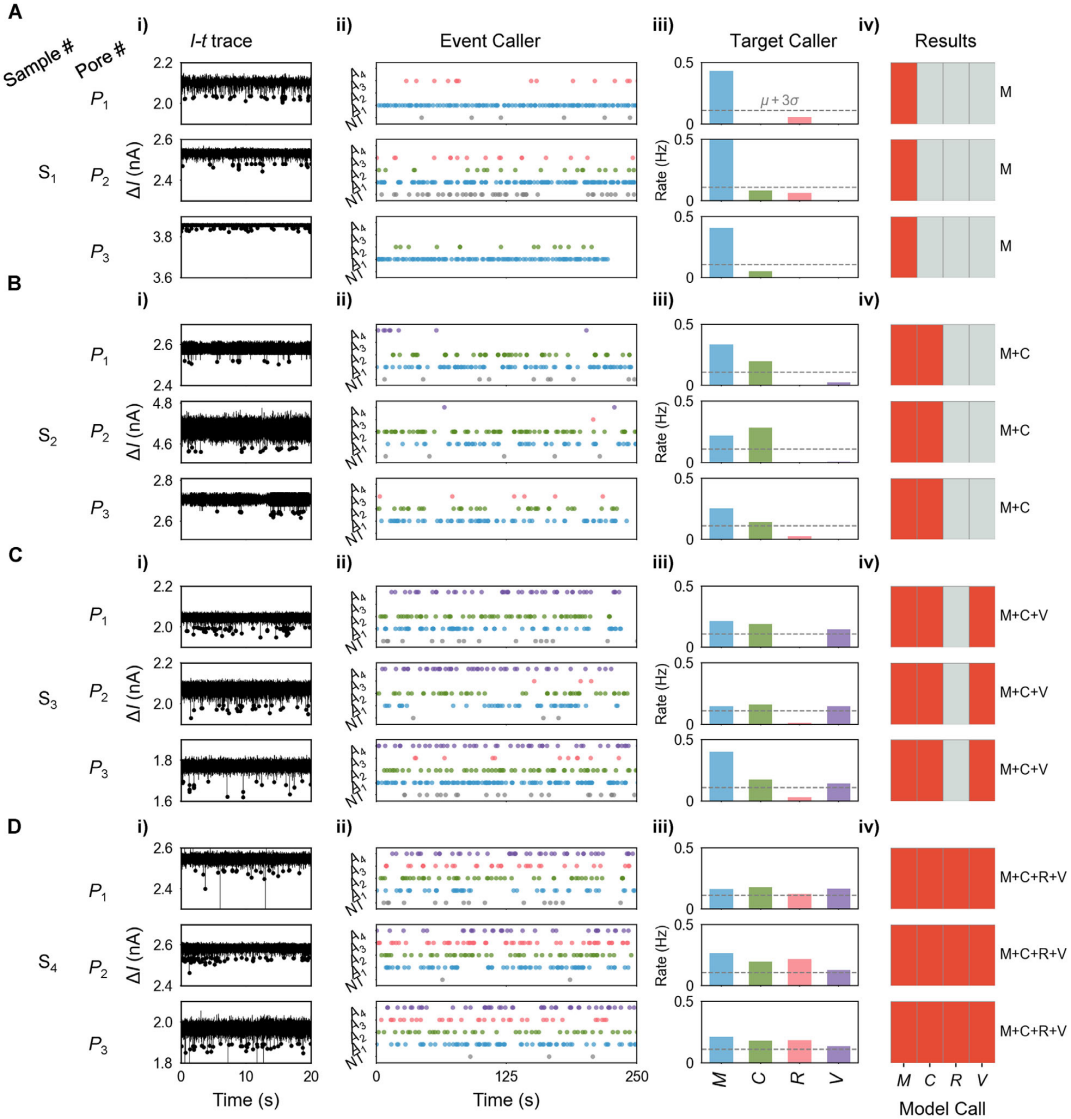
Reproducible multiplex sample‐level calling across independent nanopores. (A–D) Representative results for singleplex through four‐plex mixtures measured sequentially on three independent nanopores (P_1_, P_2,_ and P_3_) under identical conditions (1 M KCl, 400 mV). Within each row, subpanels i‐iv show: i, raw *I*–*t* trace; ii, event‐caller output with time‐stamped event‐level classifications for each target class (A_1_‐A_4_ and NT); iii, target‐caller output showing target‐resolved event‐rate histograms with the NTC‐referenced decision threshold (µ + 3σ); iv, sample‐level qualitative calls for M, C, R, and V (red, positive; gray, negative). Across nanopores, present targets produce above‐threshold event rates and positive calls, whereas absent targets remain below threshold.

To directly assess whether DNN event‐classification performance varies with nanopore diameter within the experimentally usable sensing regime, we performed a diameter‐stratified analysis of the test dataset (Figure S17). A total of 4904 events representing the five event classes, A_1_‐A_4_ and NT, were evaluated, including 1303 events acquired at 6.3 nm, 1400 at 7.5 nm, 1160 at 8.6 nm, and 1041 at 9.1 nm. The corresponding overall event‐level classification accuracies were 92.9%, 97.4%, 96.8%, and 97.8%, respectively. An additional larger‐pore comparison at 11.2 nm yielded an overall accuracy of 93.9%. Classification therefore remained robust across the selected 6.3–9.1 nm operating window. These results indicate that DNN performance is not completely invariant to nanopore diameter but remains high within the pore‐size window identified from the sensing analysis in Figure 3B. Pores below ∼6.3 nm were not included because they frequently clogged and did not provide stable event acquisition, whereas larger pores generated shallow and rapidly translocating events with reduced detectability. Accordingly, this analysis validates DNN classification performance within the selected 6.3–9.1 nm operating window rather than across all pore sizes, directly linking the physical pore‐selection criterion to event‐level decoding performance and supporting the use of this window for reproducible multiplex classification. Nanopore diameter is therefore interpreted as a sensor‐context variable that modulates the electrical response and classification fidelity, rather than as an independent molecular identifier. These results show that the event decoder and event‐rate‐based target caller produced consistent multiplex calls across the tested independent pores under the specified laboratory conditions.

### 2.6 Multiplex Target Identification in Blinded Samples

To translate event‐level nanopore classifications into sample‐level calls, we implemented a sample‐level calling framework that converts DNN‐classified events into multiplex target decisions. The target caller converts the time‐stamped classification stream into target‐resolved event rates and applies an NTC‐referenced statistical decision boundary, defined as µ + 3σ of the event rate measured across NTC replicates (Figure 7A). Using this rule, each reaction is decoded into direct binary calls for each target (M, C, R, and V) based on whether the corresponding event rate exceeds the decision threshold. This event rate‐based approach enables multiplex identification from singleplex to four‐plex reactions while maintaining negative calls when amplification products are absent, even when total event frequencies vary across samples. Representative examples spanning singleplex, duplex, triplex, four‐plex, and NTC conditions are shown in Figure 7A. These examples show that event‐level nanopore classifications can be consolidated into sample‐level multiplex decisions without evidence of systematic channel cross‐talk between encoded amplicons.

**FIGURE 7:**
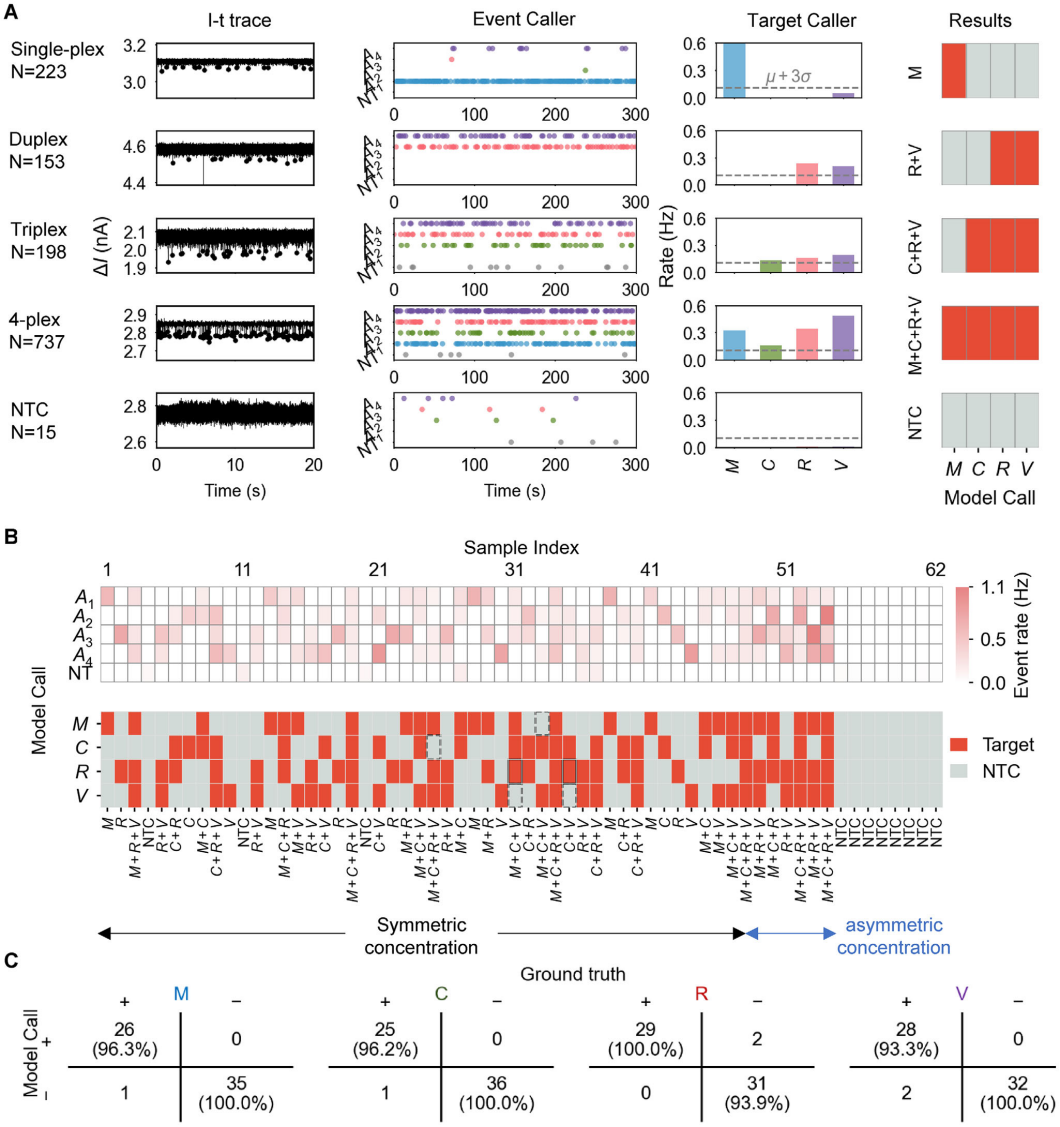
Blinded multiplex target calling using nanopore sensing and DNN‐based event classification. (A) Representative current–time (*I*–*t*) traces for five conditions: singleplex (M), duplex (R+V), triplex (C+R+V), four‐plex (M+C+R+V), and NTC. Event caller outputs (middle) show time‐stamped classifications by target; sample caller bars (right) report target‐specific event rates. The decision threshold is set to µ + 3σ relative to the true NTC baseline. (B) Blinded cohort of 62 samples spanning singleplex, duplex, triplex, four‐plex, and NTC conditions. Top: heat map of event rate by target and sample index. Bottom: model‐call matrix showing binary sample calls: red indicates target present, gray indicates a negative or target‐absent call representing NTC; red cells with a solid rectangular outline indicate false positives (FP), and gray cells with a dashed rectangular outline indicate false negatives (FN). Sample compositions are arranged from symmetric to asymmetric target concentrations, as indicated below the matrix. (C) Per‐target comparison of model calls with ground truth across the blinded cohort. Confusion matrices report true‐positive, false‐negative, false‐positive, and true‐negative counts for M, C, R, and V. Sensitivities were 96.3% (95% CI, 81.0%–99.9%) for M, 96.2% (95% CI, 80.4%–99.9%) for C, 100% (95% CI, 88.1%–100%) for R, and 93.3% (95% CI, 77.9%–99.2%) for V. Specificities were 100% (95% CI, 90.0%–100%), 100% (95% CI, 90.3%–100%), 93.9% (95% CI, 79.8%–99.3%), and 100% (95% CI, 89.1%–100%), respectively.

To evaluate the performance of the target caller under unbiased conditions, we applied the same event‐based classification and decision workflow to a blinded cohort of 62 samples spanning singleplex through four‐plex mixtures and NTC controls. The blinded cohort was acquired using sample preparations and physical nanopores that were not represented in the 66‐sample model‐development dataset; thus, both the samples and sensors were unseen during model development. This blind evaluation tests whether multiplex decisions remain accurate when sample identities are unknown and when run‐to‐run and pore‐to‐pore variability can alter event statistics. The resulting call heat map shows that target‐resolved event‐rate signals are largely confined to the expected channels and closely match the ground‐truth mixture compositions (Figure 7B), with full sample‐by‐sample outputs provided in Figure S18. Across the blinded cohort, six discrepancies were observed, including two false positives (FP) and four false negatives (FN). These errors were primarily associated with borderline event‐rate trajectories near the NTC‐referenced decision threshold or occasional event‐level misclassification, rather than with systematic channel cross‐talk. Principal component analysis (PCA) of representative correctly classified and discrepant samples showed that correctly classified samples contained dense target‐event populations with clearer separation from NT events, whereas discrepant samples contained sparse target‐consistent events near the decision boundary or low‐abundance nonspecific events assigned to an additional target class (Figure S19). Because event‐rate‐based calling could be affected by unequal target abundance, we further examined asymmetric multi‐target mixtures containing input concentrations spanning 10^1^ to 10^5^copies µL^−1^. These mixtures were designed to test whether high‐abundance targets could mask low‐abundance targets or bias event‐rate decisions. Agarose gel electrophoresis confirmed co‐amplification of the intended targets, and nanopore analysis showed that each target‐specific event rate remained above its independent NTC‐derived threshold when the corresponding target was present (Figure S20). Across samples #49‐#54, the model calls matched the expected mixture compositions, with no missed low‐abundance targets and no false positives. These asymmetric mixtures were designed to assess qualitative target recovery under unequal target inputs and were not intended to establish target‐specific limit of detection, quantitative dynamic range, or amplification efficiency. These results indicate that, in the six tested asymmetric mixtures, each intended target remained above its independently defined background threshold despite unequal target inputs.

To quantify multiplex calling performance, we computed per‐target sensitivity and specificity across the blinded samples. The target caller achieved sensitivities of 96.3% (M), 96.2% (C), 100.0% (R), and 93.3% (V), with specificities of 100% (M), 100% (C), 93.9% (R), and 100% (V) (Figure 7C). The corresponding exact binomial 95% confidence intervals (CI) were 81.0%–99.9%, 80.4%–99.9%, 88.1%–100%, and 77.9%–99.2% for the sensitivities of M, C, R, and V, respectively, and 90.0%–100%, 90.3%–100%, 79.8%–99.3%, and 89.1%–100% for the corresponding specificities. These CIs reflect the uncertainty associated with the modest number of positive and negative samples for each target; therefore, these values should be interpreted as proof‐of‐concept performance estimates rather than definitive clinical performance characteristics. The present validation was performed using synthetic DNA mixtures and therefore does not capture the complexity of clinical specimens, including excess human or microbiome DNA, fragmented nucleic acids, extraction variability, low target abundance, or RNA targets. Evaluation in representative biological matrices across broader concentration ranges will therefore be required to establish analytical sensitivity and translational robustness. Overall, these results show that the primer‐programmed multiplex RPA assay, combined with nanopore sensing and DNN‐based event decoding, produced accurate and reproducible sample‐level multiplex calls in the tested blinded synthetic‐sample cohort.

## 3 Conclusion

This work demonstrates primer‐programmed amplicon encoding as a probe‐free strategy for multiplex nucleic acid identification by coupling one‐pot RPA with solid‐state nanopore readout and deep learning‐based decoding. Primer‐programmed amplicons with predefined product lengths enable direct electrical identification of Mpox, Cowpox, Vaccinia, and human RNase P without reliance on target‐specific reporter probes or multichannel optical detection, while an assay‐specific nanopore operating window of 6.3–9.1 nm supports stable signal acquisition. Multidimensional feature‐based classification using a DNN achieves high event‐level performance, with true positive rates of 94.7%–98.9% and strong precision, sensitivity, specificity, and F1 scores. Aggregation of event‐level predictions using an NTC‐referenced decision rule enables robust sample‐level calling, with qualitative calls matching PCR‐defined target compositions across the 9 benchmark mixtures tested and reproducible performance across independent nanopores, while blinded testing demonstrates sensitivities of 93.3%–100% and specificities of 93.9%–100%. This work demonstrates a multiplexing architecture in which target identity is encoded in the physical properties of the amplification product itself rather than in an external reporter. The present four‐target synthetic system establishes the feasibility of the encoding and decoding framework but does not define the minimum resolvable amplicon‐length spacing or maximum multiplex capacity. Future scalability will depend on the usable amplicon‐length coding space, multiplex amplification robustness, and nanopore resolution, whereas clinical translation will require quantitative validation in representative biological matrices, broader concentration‐dependent testing, and systematic evaluation of target‐specific amplification efficiency. Improved translocation control and signal resolution may enable denser primer‐programmed amplicon encoding within the amplification‐compatible size range.

## 4 Methods and Experimental Section

### 4.1 Materials and Chemicals

Quantitative synthetic Mpox DNA (VR‐3270SD, 100 µL, 2.8 × 10^5^ copies µL^−1^) and Cowpox DNA (VR‐302) were purchased from ATCC. Human RNase P DNA (Hs RPP30 Positive Control, 10006626) and gBlocks gene fragments for Vaccinia were obtained from Integrated DNA Technologies (IDT). Key materials, 100 bp DNA Ladder (#N0467S), Gel Loading Dye, Purple (6X) (#B7025), and nuclease‐free water (B1500), were purchased from New England Biolabs Inc. (NEB). Additional reagents, such as TaqMan Fast Virus 1‐Step Multiplex Master Mix (#5555532) and Tris‐EDTA buffer, were acquired from Thermo Fisher and Sigma‐Aldrich. Biotium GelRed Nucleic Acid Gel Stain, 10 000× (#NC9594719), was purchased from Fisher Scientific. RPA reaction kits and enzymes (#TALQBAS01) were provided by TwistDx Ltd (Cambridge, UK). Experimental components, including quartz capillaries (0.5 mm inner diameter and 1 mm outer diameter; Sutter Instrument, USA), silver wire (0.008 inches, 25 feet), and accessories such as pipette holders (#663040), seals (#647110), and pin connectors (#647110), were sourced from A‐M Systems. A microinjector equipped with 34‐gauge needles was also purchased from World Precision Instruments.

### 4.2 RPA Assay

Design. Synthetic DNA fragments were designed from the Mpox F3L region (nt 46,344–46,453; ON563414.3), the Cowpox F3L region (nt 63,463–63,703; based on the ATCC reference sequence and similar to Cowpox Brighton, AF482758), and the Vaccinia A56R region (nt 162,298–162,746; NC_006998.1), together with the RNase P target (nt 100–409). Primers were designed to match these regions (Table S1), and sequence specificity was verified by BLAST. Primer quality was assessed using the TwistDx RPA primer‐design guidelines and the PrimedRPA open‐source Python code [53]. Candidate primers were screened for primer length, GC content, repeat regions, and sequence specificity, and potential primer interactions, including hairpin formation, self‐dimerization, and hetero‐dimerization, were evaluated to minimize predicted primer‐primer interactions. Singleplex Reaction. Each RPA reaction (Table S2) was prepared in a 25 µL total volume containing 1.25 µL each of forward and reverse primers (500 nM), 12.50 µL of 2× RPA reaction buffer (1X), 2.50 µL of 10× Basic E‐mix (1X), 2.00 µL of dNTP mix (800–2000 µM), 1.25 µL of 20× Core Reaction Mix (1X), 2.00 µL of nuclease‐free water, and 1.00 µL of the appropriate target template (Mpox, Cowpox, human RNase P, or Vaccinia; 2 × 10^5^ copies µL^−1^). 1.25 µL Magnesium acetate (Mg(AcO)_2_) was added last, at 14 mM, to prevent premature initiation of the reaction, yielding a 23.75 µL master mixture before reaction initiation. Reactions were incubated at 39°C for 20 min. Multiplex Reaction. Multiplex RPA reactions were prepared in a 25 µL total volume (23.75 µL master mixture plus 1.25 µL Mg(AcO)_2_) and were incubated at 39°C for 20 min. In duplex, triplex, and four‐plex formats, each primer was maintained at an adjustable final concentration of 400–500 nM to balance amplification across targets. The same 2× RPA buffer and 10× Basic E‐mix used in singleplex reactions were used for multiplex assays. The dNTP mix was used at a final concentration of 1000–2400 µM, depending on the multiplex level. The 20× Core Reaction Mix was adjusted 1.0–1.6× to support higher multiplex complexity. Template inputs were 10^5^ copies µL^−1^ stocks for duplex, triplex, and four‐plex reactions to maintain the total template volume. Mg(AcO)_2_ was added as the final step to initiate the reaction. Detailed formulations are provided in Tables S3–S5. Gel analysis of RPA amplicons. After RPA, the amplicons were resolved by 3% agarose gel electrophoresis at 6 V/cm using GelRed stain. The bands of interest were then analyzed using the BIO‐RAD GelDoc Go Imaging System with a 20‐s exposure.

### 4.3 PCR Assay

PCR reactions (Tables S6–S9) were prepared in a 20 µL total volume using TaqMan Fast Virus 1‐Step (Multiplex) Master Mix at 1× final concentration. Primer concentrations were maintained at 600 nM final for singleplex reactions and adjusted to final concentrations of 400–600 nM final per primer for duplex, triplex, and four‐plex formats to balance amplification across targets. Target templates were prepared at a concentration of 10^5^ copies µL^−1^. Amplicons were resolved on a 3% agarose gel run at 6 V/cm with GelRed staining, and bands were imaged using a BIO‐RAD GelDoc Go Imaging System with a 20‐s exposure.

### 4.4 Nanopore Fabrication and Measurement

Quartz capillaries were cleaned in a Piranha solution (H_2_SO_4_:H_2_O_2_ = 3:1) at 85°C for 30 min, then rinsed with deionized water and dried in a vacuum oven at 120°C for 20 min. Nanopores were fabricated using a laser pipette puller (P‐2000, Sutter Instruments, USA) under a single‐line protocol (heat 650, filament 4, velocity 60, delay 145, pull 160–215) and two‐line protocol (Line 1: “heat 750, filament 5, velocity 50, delay 140, and pull 50” and Line 2: “heat 715, filament 4, velocity 30, delay 145, and pull 215–245” signal‐to‐noise ratio) [54, 55, 56]. The finished pores were filled with either 1 M KCl, 2 M LiCl, or 4 M LiCl in Tris‐EDTA buffer (pH 8.0), and electrolyte solutions were filtered through a 0.2 µm syringe filter (WHA67802502, Whatman) before use [57]. Amplification products were passed through the same 0.2 µm filter and diluted 20‐fold prior to measurement, so that the recorded signals originated predominantly from the target amplicons. A constant voltage was applied using a NI‐6363 DAQ card (National Instruments, USA), and the ionic current was amplified with an Axopatch 200B amplifier (Molecular Devices, USA) [54, 55]. Data were sampled at 100 kHz, low‐pass filtered at 10 kHz in LabVIEW, and recorded inside a custom Faraday cage using Ag/AgCl electrodes in the cis and trans chambers, with the cis side grounded.

### 4.5 Data Collection and Analysis

Nanopore data for DNN training were collected from 66 samples measured using 21 nanopores in Figure S14. The diameters of these model‐training pores ranged from 4.70 to 11.44 nm, confirming that the training data spanned a broad pore‐size distribution (Figure S15F). Approximately 100–300 translocation events were acquired per sample, corresponding to an expected Poisson counting uncertainty of ∼10%–5.8%, respectively [44]. For each event, ΔI, Δt, I_B_, and I_RMS_ were extracted within a 1000 ms window centered on the event, comprising 500 ms before and 500 ms after the event. I_B_ and I_RMS_ were treated as event‐specific local descriptors rather than fixed global values for an entire pore or recording. ΔI was calculated relative to the event‐specific local baseline, reducing the influence of absolute current offsets between pores and slow baseline drift within a recording. These acquisition and quality‐control criteria minimized the influence of temporal drift on ΔI and Δt during individual measurements. For feature‐space visualization, t‐SNE was applied to standardized six‐feature input vectors and to the penultimate‐layer activations extracted from the trained DNN. To assess the pore‐size dependence of DNN performance within the selected broad diameter window, overall event‐level accuracy was calculated separately for each nanopore diameter as the proportion of events correctly assigned to A_1_‐A_4_ or NT. Post‐processing and peak extractions were performed using custom Python 3.13.3 scripts, keeping the signal‐to‐noise ratio (SNR) >3. Peaks were detected using a threshold of µ′ + 3σ′, where µ′ and σ′ denote the mean and standard deviation of the baseline current. A held‐out validation dataset consisting of 23 samples measured on independent nanopores was used for intermediate model evaluation (Figure S16). A separate 62‐sample blinded cohort was subsequently evaluated using sample preparations and physical nanopores that were not represented in the 66‐sample development dataset. This cohort was not used for feature selection, hyperparameter optimization, or model weight updating and therefore served as an independent unseen‐sample and unseen‐pore evaluation of the fixed classifier. For sample‐level calling, NTC event rates were calculated by dividing the number of detected events by the corresponding acquisition duration for each replicate. Approximate normality was evaluated using the Shapiro‐Wilk test and Q‐Q analysis. The sample‐level decision threshold was defined as µ + 3σ of the NTC event‐rate distribution and was applied only after DNN event classification. Sample‐level sensitivity and specificity are reported with two‐sided 95% exact binomial CI calculated using the Clopper–Pearson method.

## Author Contributions

M.A.A. designed and validated the assay, developed methodology, conducted investigations, conceptualized, coded, acquired data, performed formal analysis, conducted data analysis, and wrote the original draft. M.A.U.K. handled data visualization, review & editing, and supervised data analysis. W.U.Z. contributed ideas for DNN implementation, assisted with model optimization, and provided insights into improving model performance. A.J.P. wrote the necessary programming code for data analysis. W.G. conceived and designed the project, provided resources, secured funding, administered the project, supervised the overall experimental design, and reviewed & edited the manuscript. All authors read and approved the final manuscript.

## Conflicts of Interest

The authors declare no conflicts of interest.

## Supporting information




**Supporting File**: advs78381‐sup‐0001‐SuppMat.pdf.

## Data Availability

The data are available from the corresponding author upon reasonable request.
