# Supplementary material for: Probe‐Free, Primer‐Programmed Amplicon Encoding Enables Nanopore‐Based Multiplex Detection With Deep Learning Decoding
Source: Adv Sci (Weinh). 2026 Oct 22:e78381. Online ahead of print. doi: 10.1002/advs.78381 (PMC13690978; doi:10.1002/advs.78381)
Supplement: Supplementary file 1 [file ADVS-9999-e78381-s001.pdf]

Probe-Free, Primer-Programmed Amplicon Encoding Enables Nanopore-Based Multiplex Detection with Deep Learning Decoding

Md. Ahasan Ahamed[1,2], Muhammad Asad Ullah Khalid[1], Wahid Uz Zaman[3], Anthony J. Politza[4], and Weihua Guan[1*]

[1]Department of Intelligent Systems Engineering, Indiana University, Bloomington, IN, 47408, United States
[2]Department of Electrical Engineering, Pennsylvania State University, University Park, PA, 16802, United States
[3]Department of Computer Science and Engineering, Pennsylvania State University, University Park, PA, 16802, United States
[4]Department of Biomedical Engineering, Pennsylvania State University, University Park, PA, 16802, United States

* Corresponding author: guanw@iu.edu

Table of contents **Page**

Section 1. Conical nanopore diameter formula approximation -------------------------- 2
Section 2. Supplementary Figures and Legends-- 3-30
Section 3. Supplementary Table-- 31-34

Section S1. Conical Nanopore Diameter Formula Approximation

The total resistance (R) of a conical nanopore: $R=\frac{1}{\sigma}(\frac{4l}{\pi d_b d_t}+\frac{1}{d_b}+\frac{1}{d_t})$ where σ is the electrolyte conductivity (S/m), d_b is the base diameter of the nanopore (µm), d_t is the tip diameter of the nanopore (µm), and l is the nanopore length (µm).

Now, neglect the term access resistance $\frac{1}{d_b}+\frac{1}{d_t}$, Access resistance refers to the electrical resistance encountered by ions as they enter or exit the nanopore from the surrounding electrolyte. In this case, it contributes ~0.1% to the total measured resistance (R), indicating that the pore resistance is the dominant component. Then, the equation becomes $R=\frac{1}{\sigma}.\frac{4l}{\pi d_b d_t}$. We know the conductance G=1/R, so $G=\sigma.\frac{\pi d_b d_t}{4l}$. Consider $d_t = D$

So, the nanopore (tip) diameter becomes $D=\frac{4Gl}{\pi\sigma d_b}$....................................... (**Equation S1**)

Section S2. Supplemental Figures and Legends

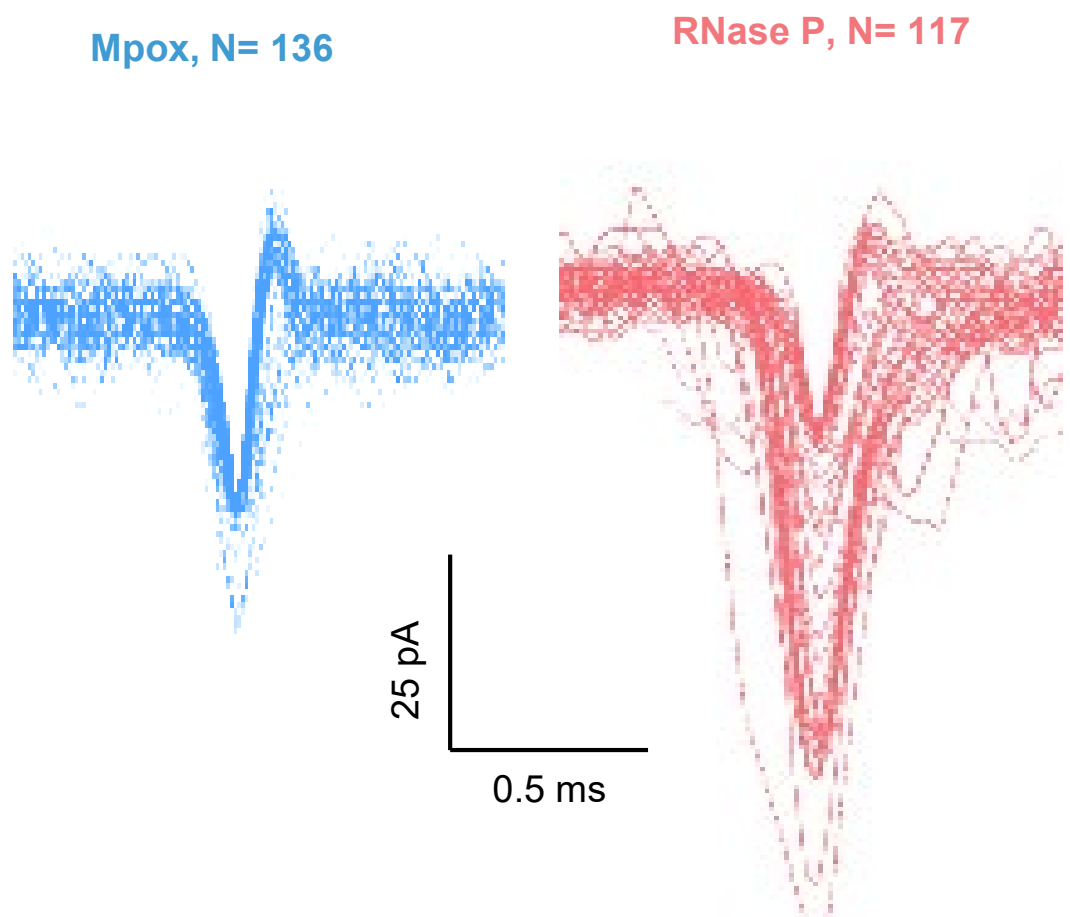

M

Event #	I_B (nA)	I_{RMS} (pA)	ΔI (pA)	Δt (ms)	t (s)
1	1.49989	5.18654	28.88034	0.67000	4.52688
2	1.49689	5.17617	28.82258	9.02192	0.23091
3	1.49839	5.18136	28.85146	2.86713	3.17446
⋮	⋮	⋮	⋮	⋮	⋮
135	1.49069	5.05751	61.11873	3.16049	1.84095
136	1.49505	5.07273	62.59168	4.28000	0.48245

R

Event #	I_B (nA)	I_{RMS} (pA)	ΔI (pA)	Δt (ms)	t (s)
1	1.50210	6.05391	57.79233	1.36000	0.26348
2	1.49910	6.04180	63.65788	1.55688	4.52587
3	1.50060	6.04786	74.66757	0.89910	2.61870
⋮	⋮	⋮	⋮	⋮	⋮
116	1.51790	6.99478	62.46650	4.27144	0.48149
117	1.51078	7.00879	67.10334	1.20000	0.69680

Figure S1. Extracted waveform features used for DNN classification. The left panel shows representative ionic current signals from a nanopore measurement, with annotated extraction of the first four key features used in model training: baseline current (I_B), RMS current (I_{RMS}), current blockage amplitude (ΔI), and dwell time (Δt). These features were derived from individual translocation events and reflect both pore characteristics and amplicon-specific properties. The right panel shows the extracted feature table from the left figures.

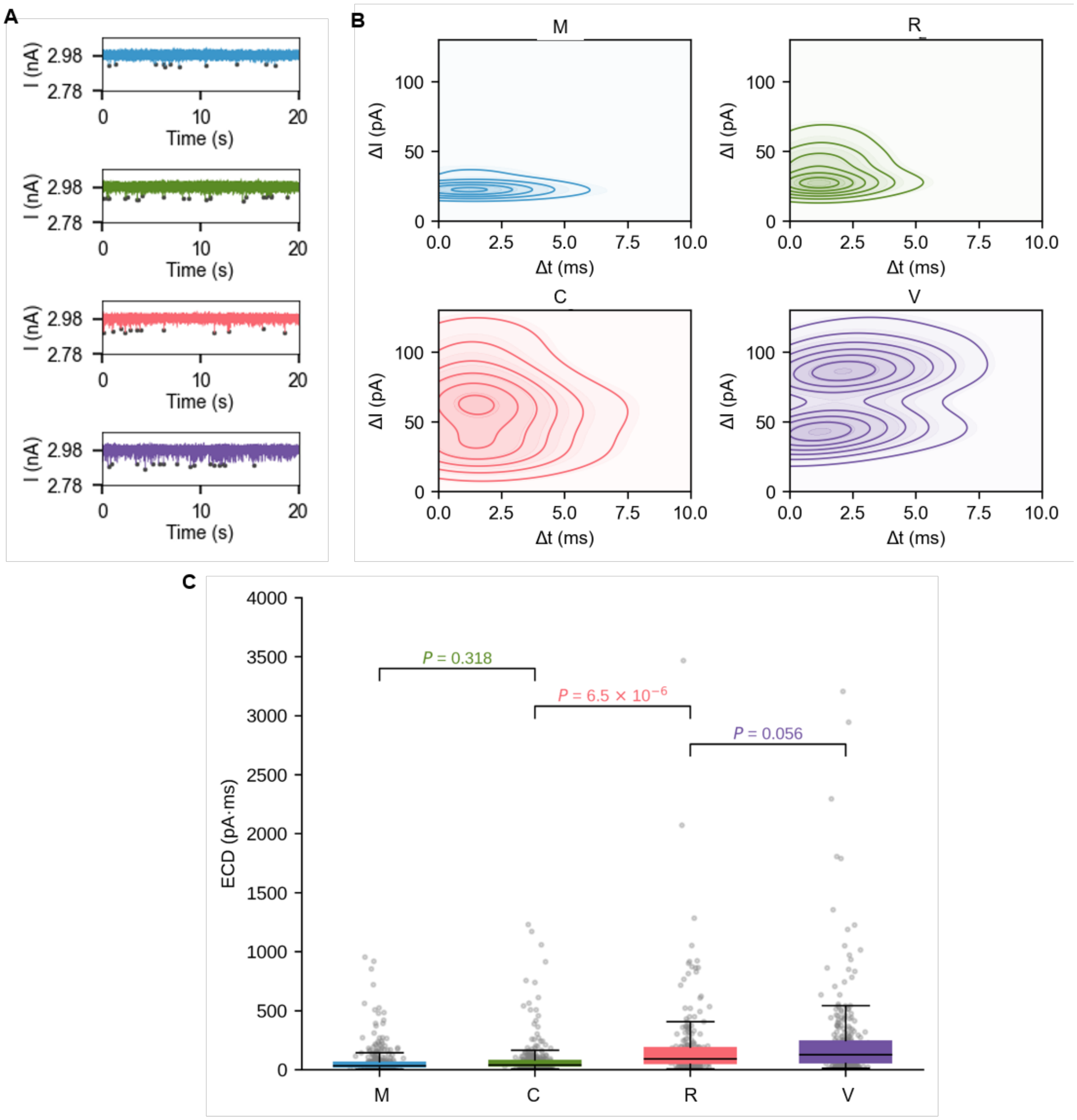

Figure S2. Statistical limitations of classification for primer-programmed RPA amplicons. A) A solid-state nanopore was used to record ionic current traces at 400 mV. **B)** Contour plots of blockade amplitude (ΔI) versus dwell time (Δt) reveal broad, skewed event distributions with substantial overlap between targets. **C)** Event charge deficit (ECD, ΔI × Δt) distributions for M, C, R, and V. Boxes indicate the interquartile range, center lines denote the median, whiskers extend to 1.5× the interquartile range, and gray points represent individual events. Event counts were n = 324 (M), 332 (C), 235 (R), and 303 (V). Adjacent targets were compared using two-sided Welch's t-tests, yielding P = 0.318 for M versus C, $P = 6.5 \times 10^{-6}$ for C versus R, and P = 0.056 for R versus V. Two of the three adjacent comparisons were not statistically significant, indicating that ECD alone does not consistently resolve the four target classes.

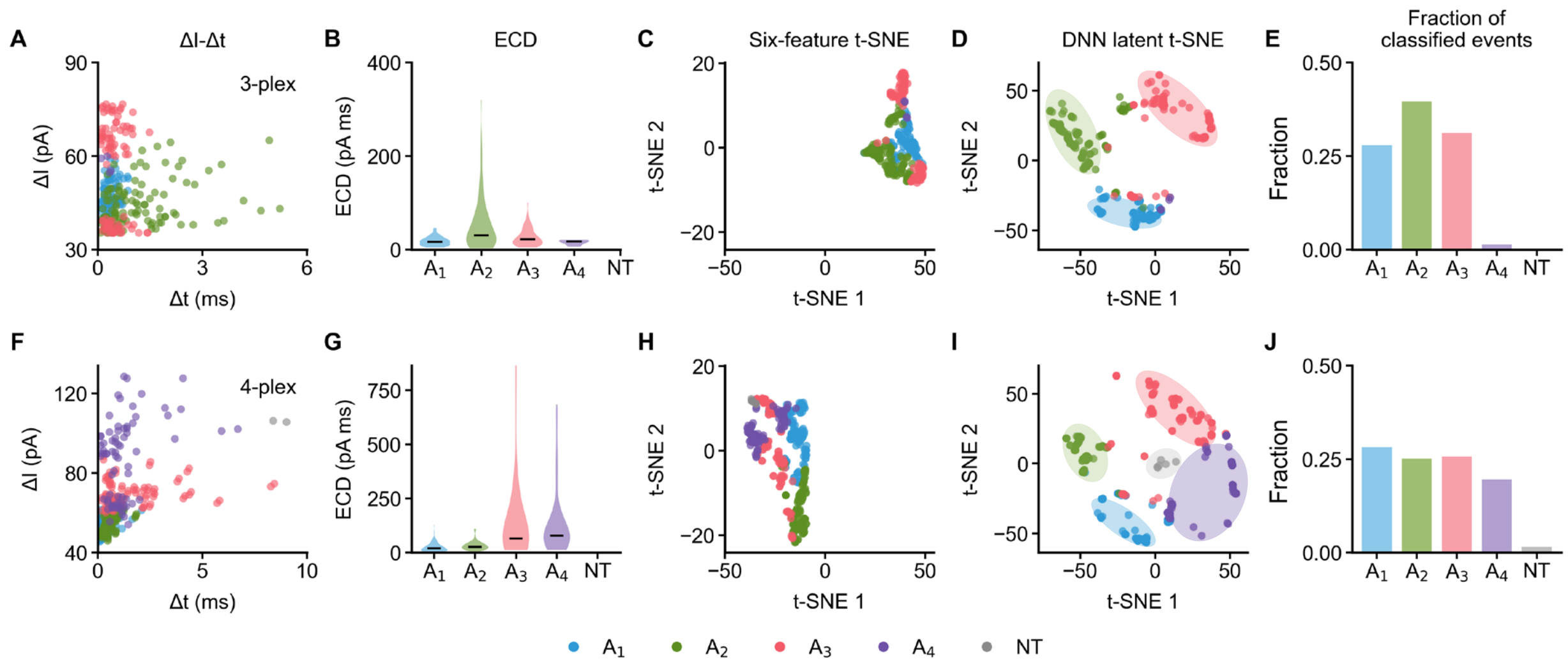

Figure S3. Multidimensional feature-space visualization of DNN-based discrimination of overlapping nanopore signals. We analyzed representative 3-plex and 4-plex samples to compare conventional nanopore descriptors with multidimensional feature representations. **A, F)** ΔI-Δt scatter plots show substantial overlap among amplicon-associated events. **B, G)** ECD distributions show overlapping event-charge profiles, indicating that ECD alone is insufficient for reliable event discrimination. **C, H)** t-SNE projections of standardized raw six-feature input vectors, including baseline current, RMS current, ΔI, Δt, inter-event interval, and nanopore diameter, reveal class-associated structure that is not apparent in the ΔI-Δt or ECD distributions. **D, I)** t-SNE projections of the penultimate-layer DNN latent representation show improved class organization after nonlinear transformation by the trained network. **E, J)** Fractions of classified events show the DNN-assigned event composition for representative 3-plex and 4-plex samples. t-SNE axes are dimensionless embedding coordinates and do not correspond to physical nanopore parameters. t-SNE was used only for visualization.

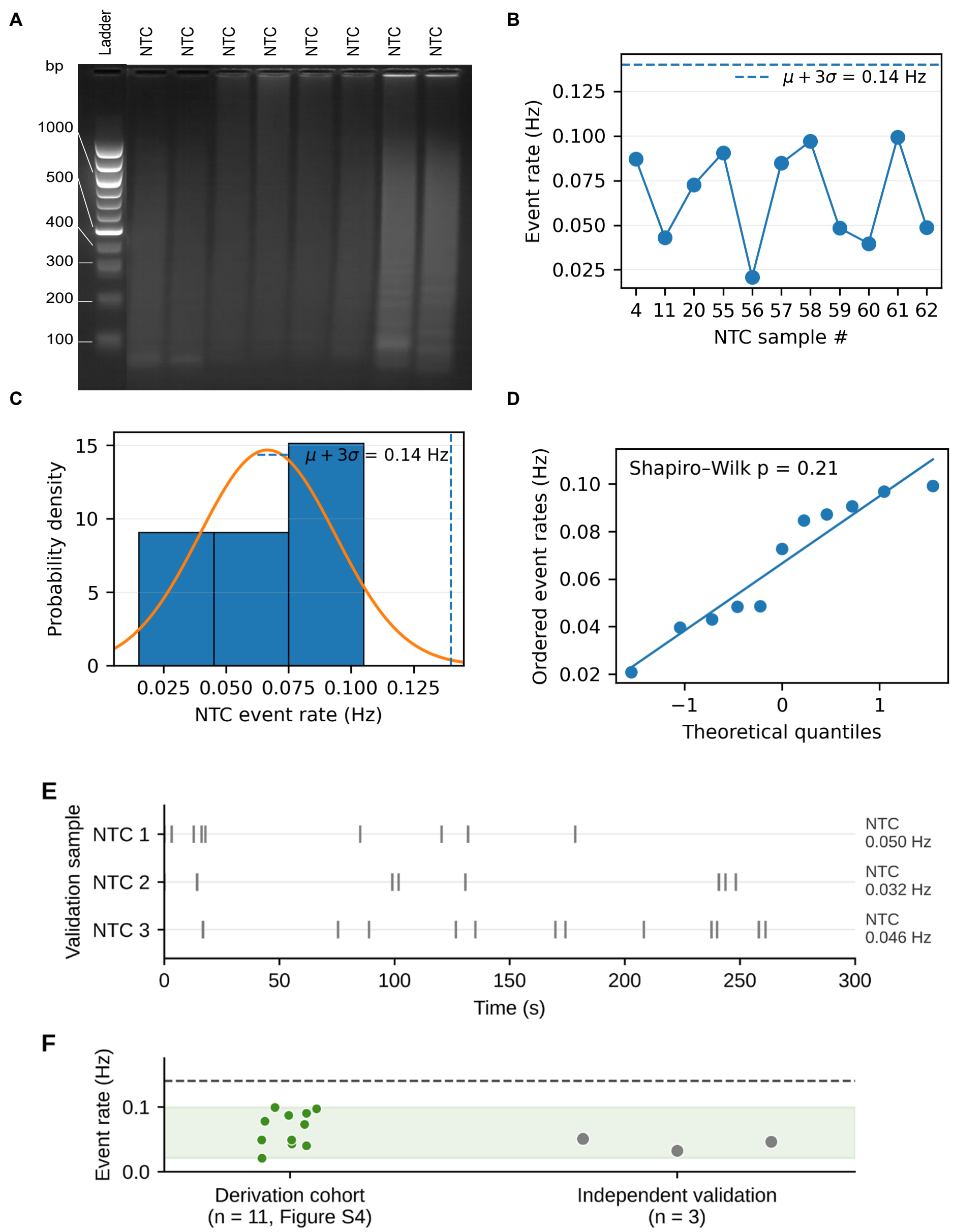

Figure S4. Statistical characterization of the NTC background used for sample-level threshold definition. A) Representative agarose gel electrophoresis of NTC measurements showing low-level nonspecific background products across independent reactions. **B)** Event rates calculated for 11 NTC measurements by normalizing the number of detected events to the corresponding acquisition time. The dashed line indicates the sample-level decision threshold defined as $\mu + 3\sigma = 0.14$ Hz. **C)** Distribution of NTC event rates with a fitted normal probability-density function and the corresponding $\mu + 3\sigma$ threshold. **D)** Q–Q analysis of the NTC event-rate distribution. The Shapiro–Wilk test showed no significant departure from normality ($p = 0.21$), supporting the use of the NTC-derived statistical boundary under the tested conditions. **E**) Event-level outputs for three independent NTC reactions measured on nanopores not represented in panels B to D, with each tick marking one detected event over a common 0 to 300 s display window and the measured event rate stated at the right. **F**) Event rates of the threshold-defining ($n = 11$) and validation ($n = 3$) NTC sets; the shaded band marks the threshold-defining range. We applied the threshold defined in panels B and C without recalculating μ or σ; all three validation measurements fell below it.

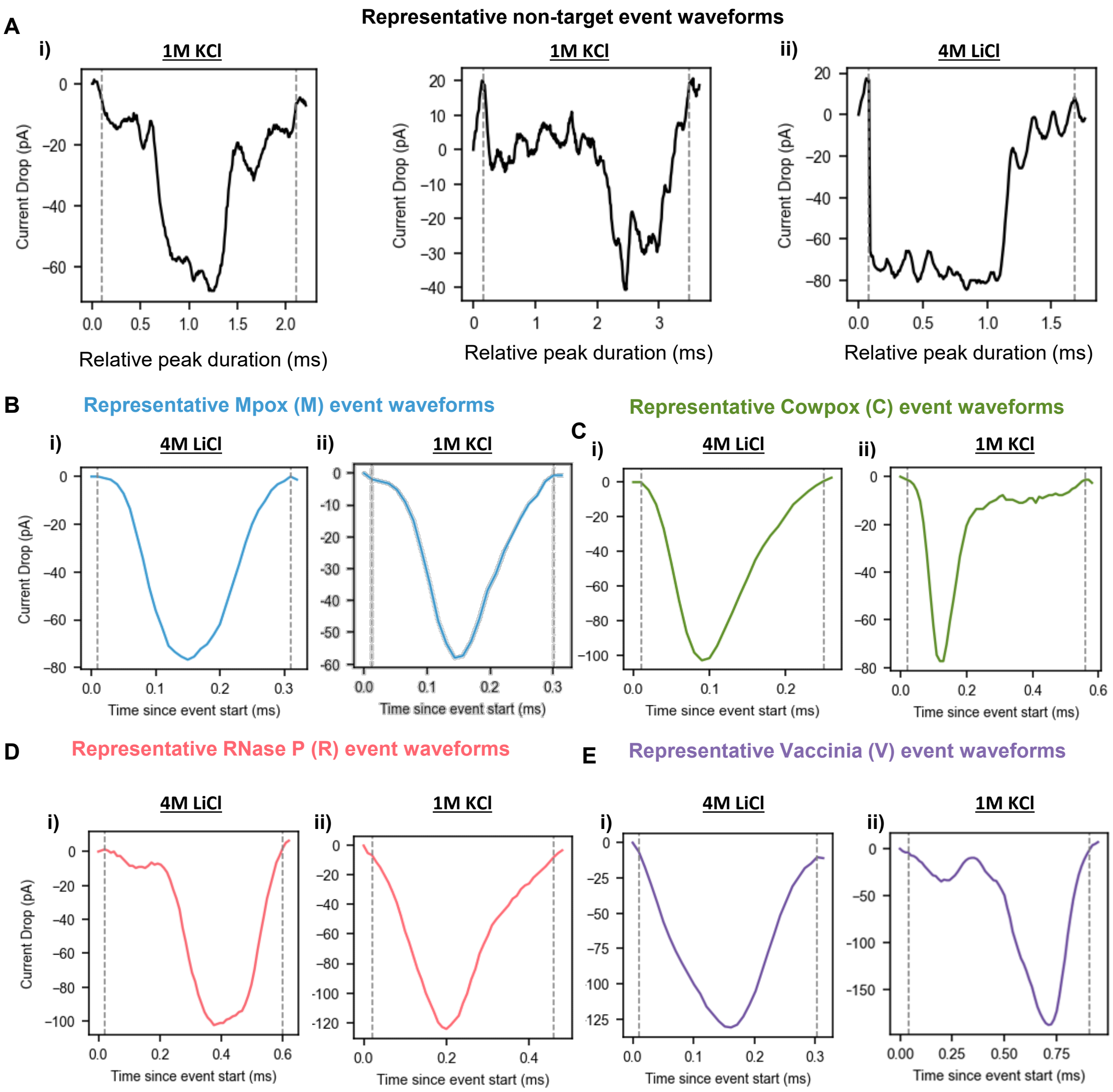

Figure S5. Representative nanopore translocation waveforms for NT and primer-programmed amplicons under different salt conditions. Blockade amplitude (ΔI) and dwell time (Δt) characteristics for NT (non-target control) and targets M, C, R, and V measured under two salt compositions: **(i)** 1 M KCl and **(ii)** 4 M LiCl. **A)** Representative waveform of NT. **B-E)** Representative waveforms of M, C, R, and V.

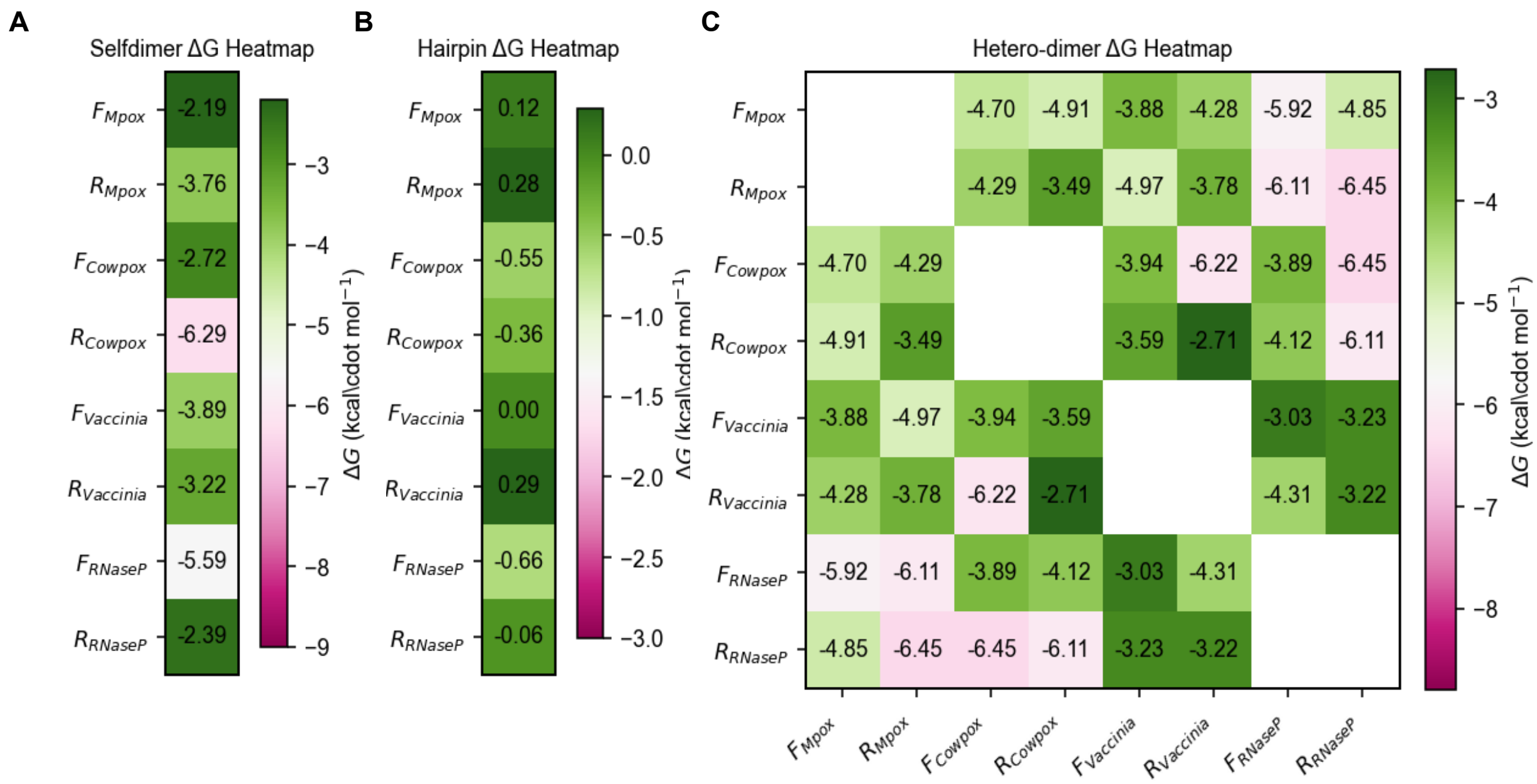

Figure S6. Thermodynamic screening of multiplex primer interactions. A) Self-dimer free energy (ΔG) heatmap for each forward and reverse primer in the Mpox, Cowpox, RNase P, and Vaccinia assays. **B)** Hairpin ΔG heatmap for the same primer set, indicating minimal predicted secondary structure. **C)** Heterodimer ΔG heatmap for all pairwise primer combinations within and across assays, used to evaluate cross-dimerization risk in the one-pot multiplex reaction. For all interaction modes, we applied a conservative acceptance threshold of ΔG>-9 kcalmol^{-1}.

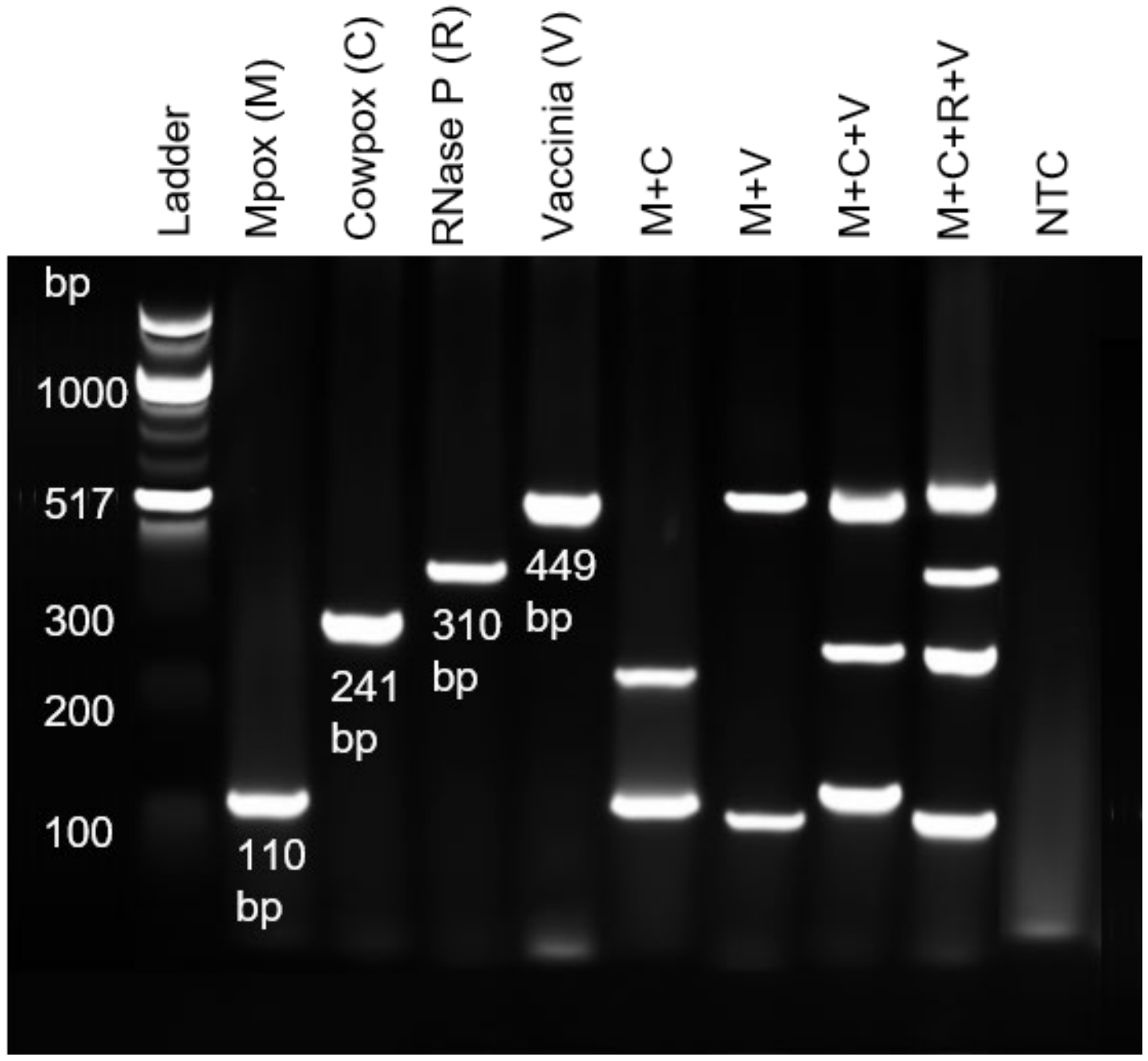

Figure S7. PCR validation of multiplex assay. Agarose gel of singleplex, duplex, triplex, and four-plex PCR products. Bands at 110, 241, 310, and 449 bp match the designed product sizes. The NTC shows no intense band. Conditions: 3% agarose, GelRed stain, electrophoresis at 6 Vcm^{-1}.

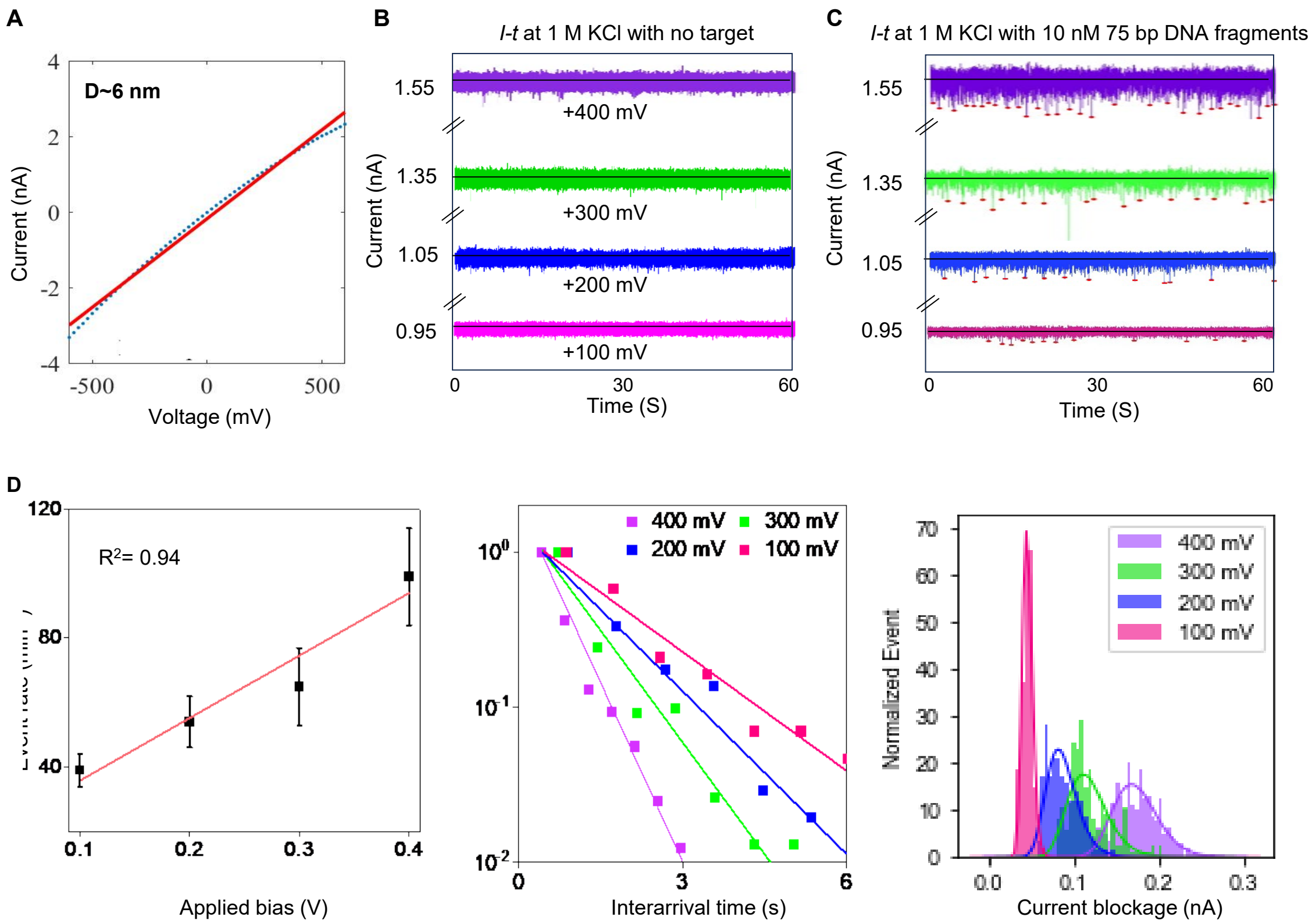

Figure S8. Electrical characterization and voltage-dependent sensing of 10 nM 75 bp DNA fragments at 1M KCl. A) Current–voltage (I–V) response of the nanopore measured from +100 mV to +500 mV. The linear fit yields a conductance of 4.6970 nS, consistent with a pore diameter of approximately 6 nm. **B)** Representative baseline current traces recorded under applied biases of 400 mV, 300 mV, 200 mV, and 100 mV, respectively. Each trace shows stable ionic conduction over a 60 s acquisition window. **C)** Representative current versus time (*I-t*) traces recorded at applied voltages of 100, 200, 300, and 400 mV. **D)** Event rate as a function of applied voltage, showing approximately linear scaling over the range 100 to 400 mV. **E)** Interarrival time distributions of translocation events at each applied voltage, which follow the Poisson distribution $e^{-\lambda t}$. **F)** Histograms of current blockage amplitudes for the four voltages, illustrating the voltage-dependent distribution of event magnitudes.

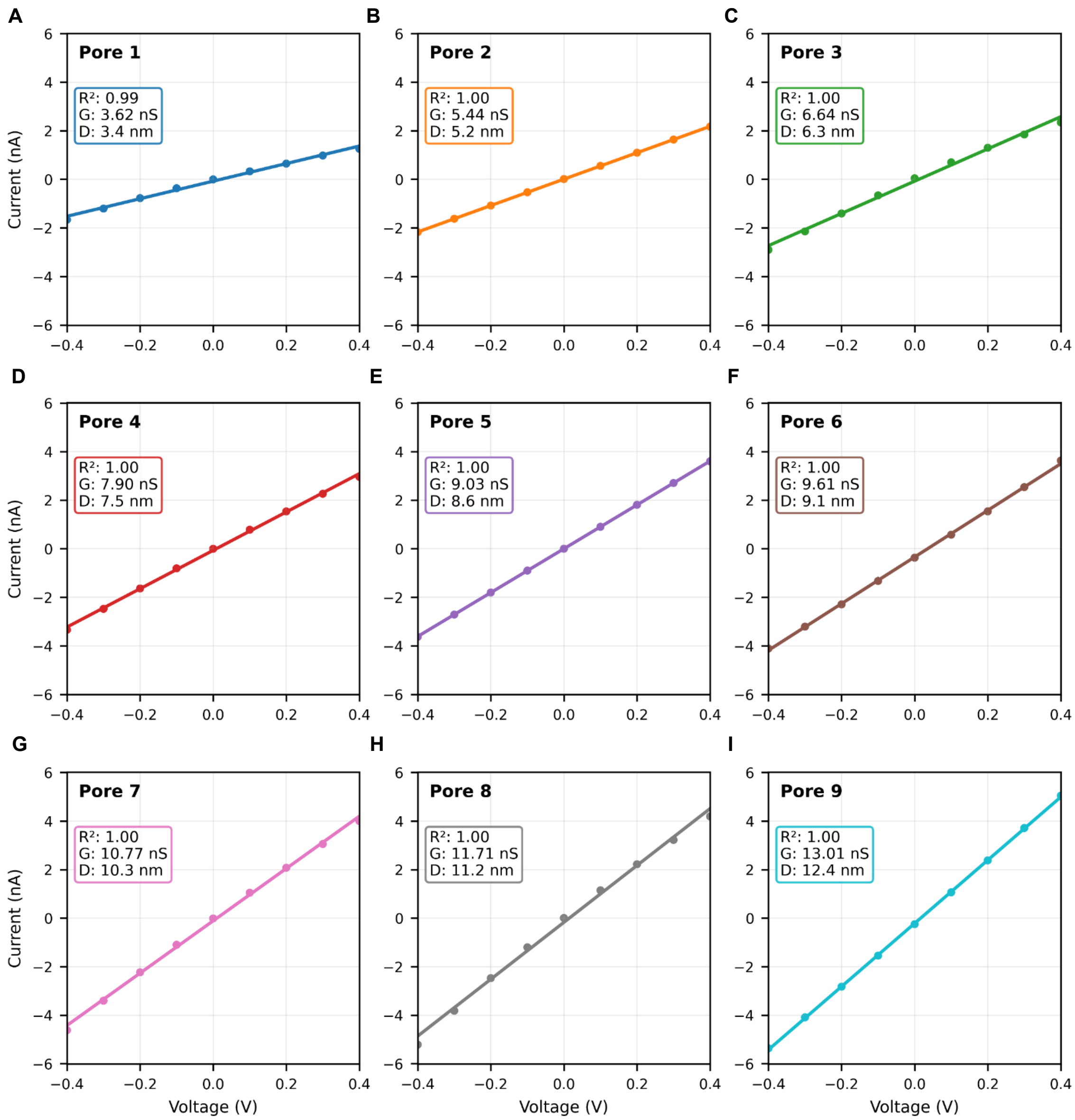

Figure S9. Determination of nanopore diameter from ionic conductance in the linear ohmic regime. Representative current–voltage (I–V) characteristics of nine independent conical solid-state nanopores (Pore 1–9, **A-I**) measured in 1 M KCl. For each pore, the current was recorded over the voltage range −0.4 to +0.4 V, and the conductance (G, nS) was extracted from a linear fit restricted to the ohmic region. The high linearity of all traces ($R^2 \geqslant 0.99$) confirms that the measurements lie within the linear regime, validating the conductance-based diameter approximation. The fitted conductance and the corresponding tip diameter (D), calculated using **Eq. (S1),** are annotated in each panel. The estimated diameters span 3.4–12.4 nm (G = 3.62–13.01 nS).

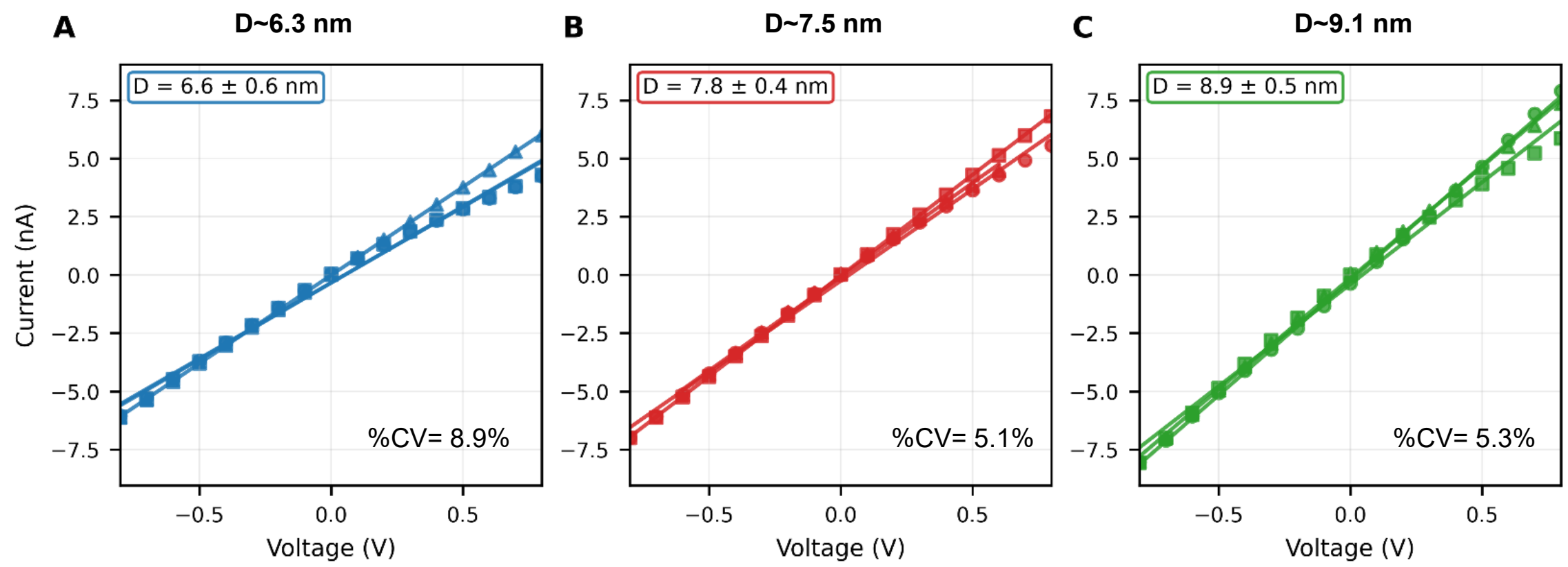

Figure S10. Reproducibility of nanopore diameter estimation within nominal size groups. Replicate current–voltage (I–V) measurements for three representative nanopore size classes: **A-C)** D ≈ 6.3 nm, 7.5 nm, and 9.1 nm. Multiple independent pores within each group were measured in 1 M KCl over the −0.8 to +0.8 V range, and conductance was extracted from linear fits in the ohmic regime. The mean estimated diameter with its standard deviation is annotated in each panel (D = 6.6 ± 0.6 nm, 7.8 ± 0.4 nm, and 8.9 ± 0.5 nm, respectively), and the coefficient of variation (%CV = 8.9%, 5.1%, and 5.3%) quantifies the spread across replicate pores. The consistently low %CV (≤ 8.9%) demonstrates that the conductance-based diameter estimation is reproducible.

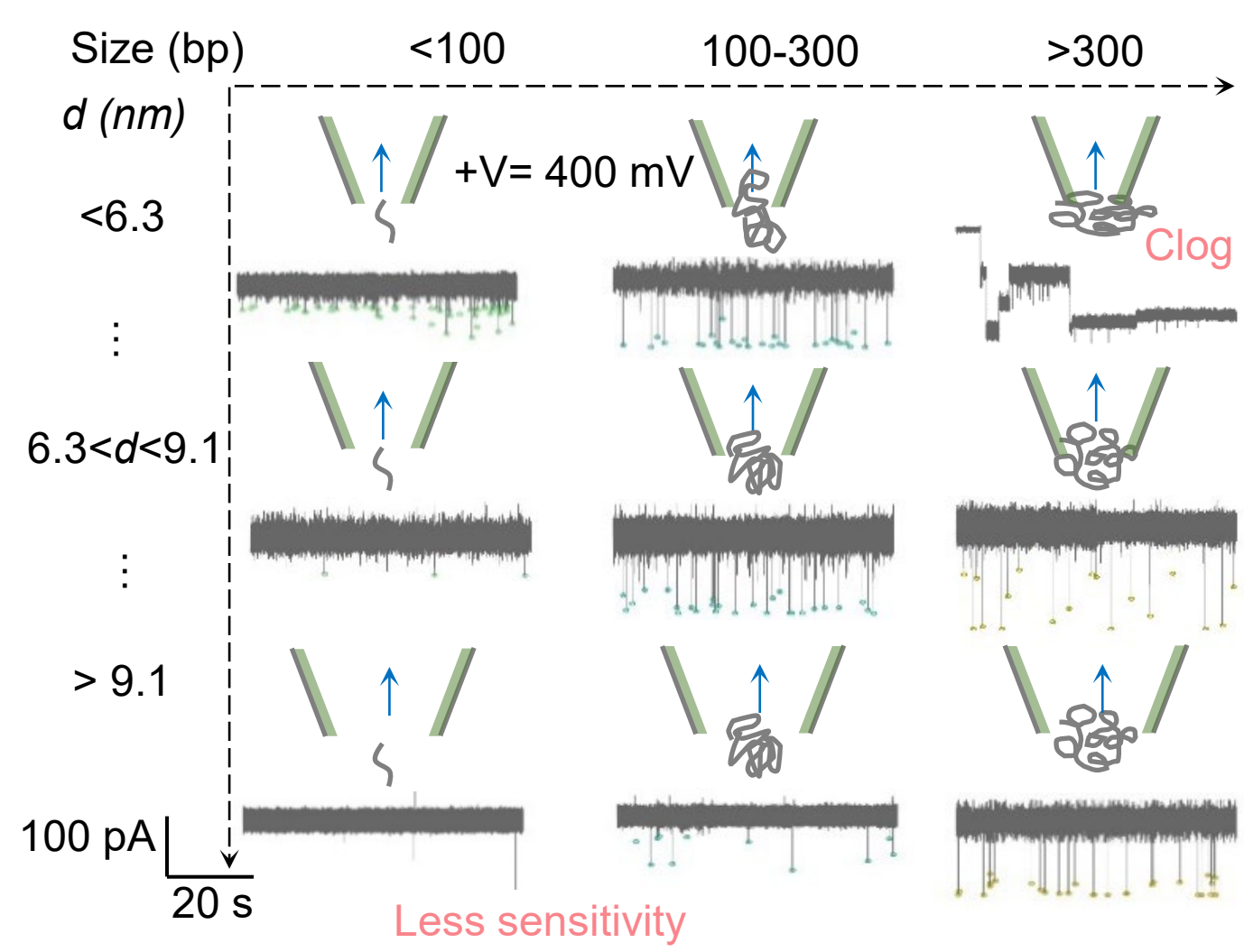

Figure S11. Transport physics underlying the size dependence of nanopore diameter and target length. Schematic overview of how nanopore diameter and DNA fragment size jointly determine translocation behavior and sensing performance. Large pores (>9.1 nm) yield ultrafast transport for short fragments (75–100 bp), lower sensitivity, whereas small pores (<6.3 nm) promote clogging-prone transport for long fragments (300–500 bp). Intermediate pore sizes (6.3<d<9.1 nm) provide a balance that supports well-resolved events across the full amplicon size range.

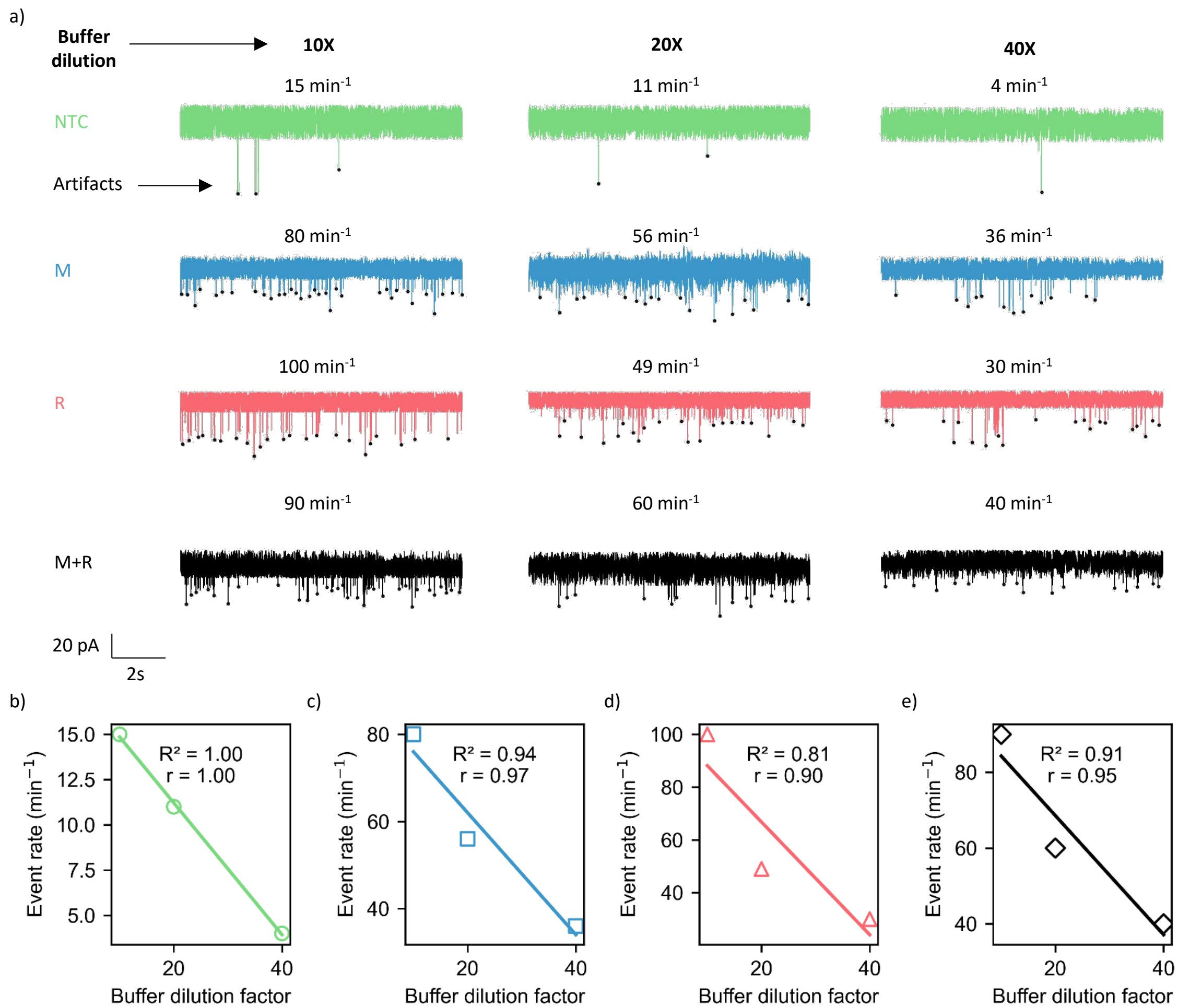

Figure S12. Event measurements using a 6.3 nm nanopore. A) The effect of buffer dilution on the current–time (I–T) trace. **B-E)** Linear plots for NTC, M R, and M+R duplex samples, with corresponding R^2 and r values indicated.

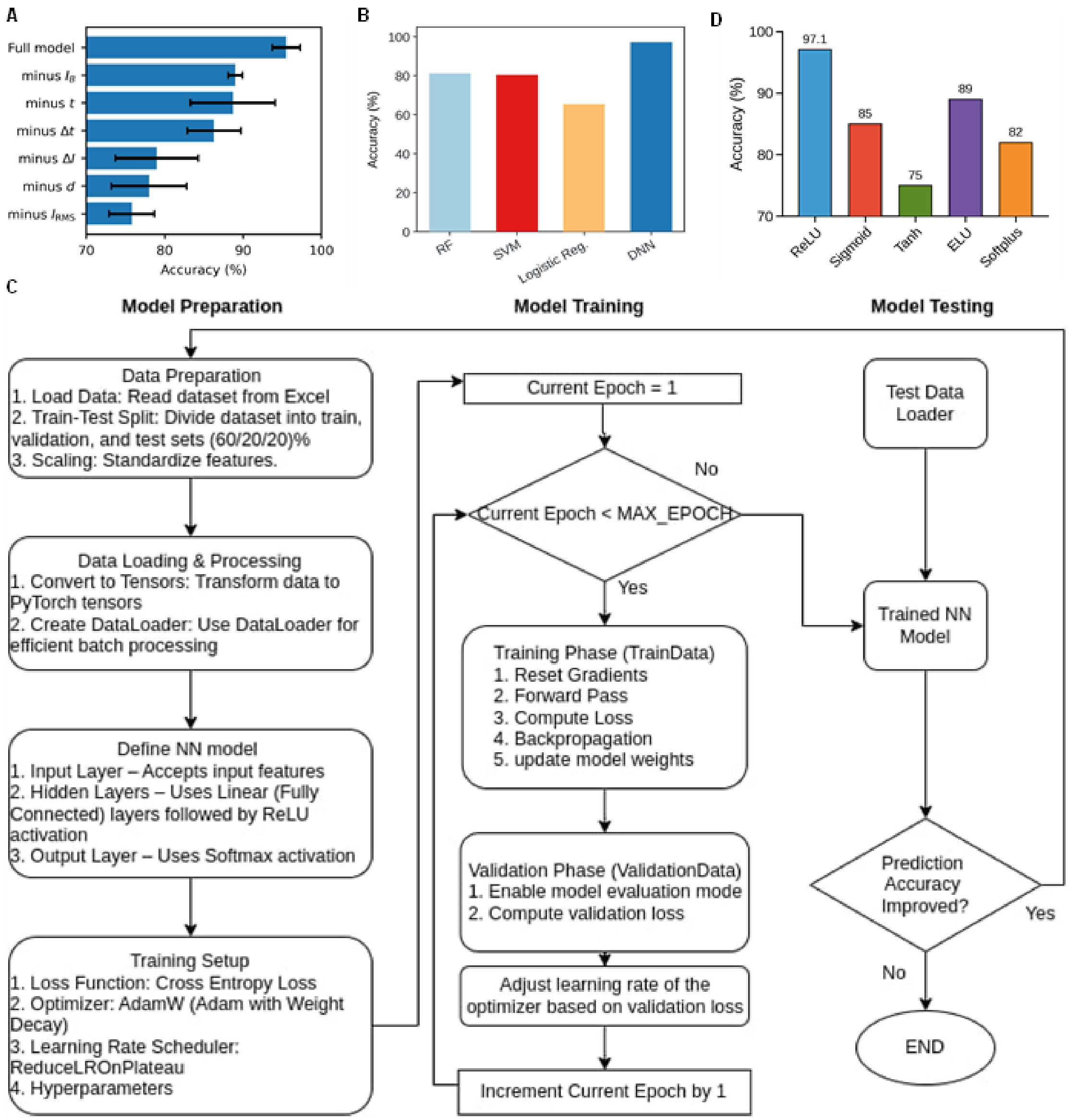

Figure S13. Selection and development workflow of the DNN event classifier. A) One-feature-at-a-time ablation analysis of the selected six-feature representation, showing mean classification accuracy ± SD across 8 repeated runs. **B)** Comparison of event-classification accuracy using random forest (RF), support vector machine (SVM), logistic regression, and DNN with the same six-feature representation and data-partitioning framework. The DNN achieved the highest accuracy of 96.7%, compared with 81.43% for RF, 80.50% for SVM, and 65.65% for logistic regression. **C)** End-to-end workflow for DNN model preparation, training, validation, and testing. Event-level features were standardized and partitioned into training, validation, and internal test subsets. Model parameters were optimized using cross-entropy loss and backpropagation, while validation performance was used for learning-rate adjustment and model selection. The internal test subset was excluded from model weight optimization. **D)** Comparison of activation functions using the same DNN framework. ReLU achieved the highest accuracy and was retained for subsequent model development.

I–t traces of targets used for DNN model training

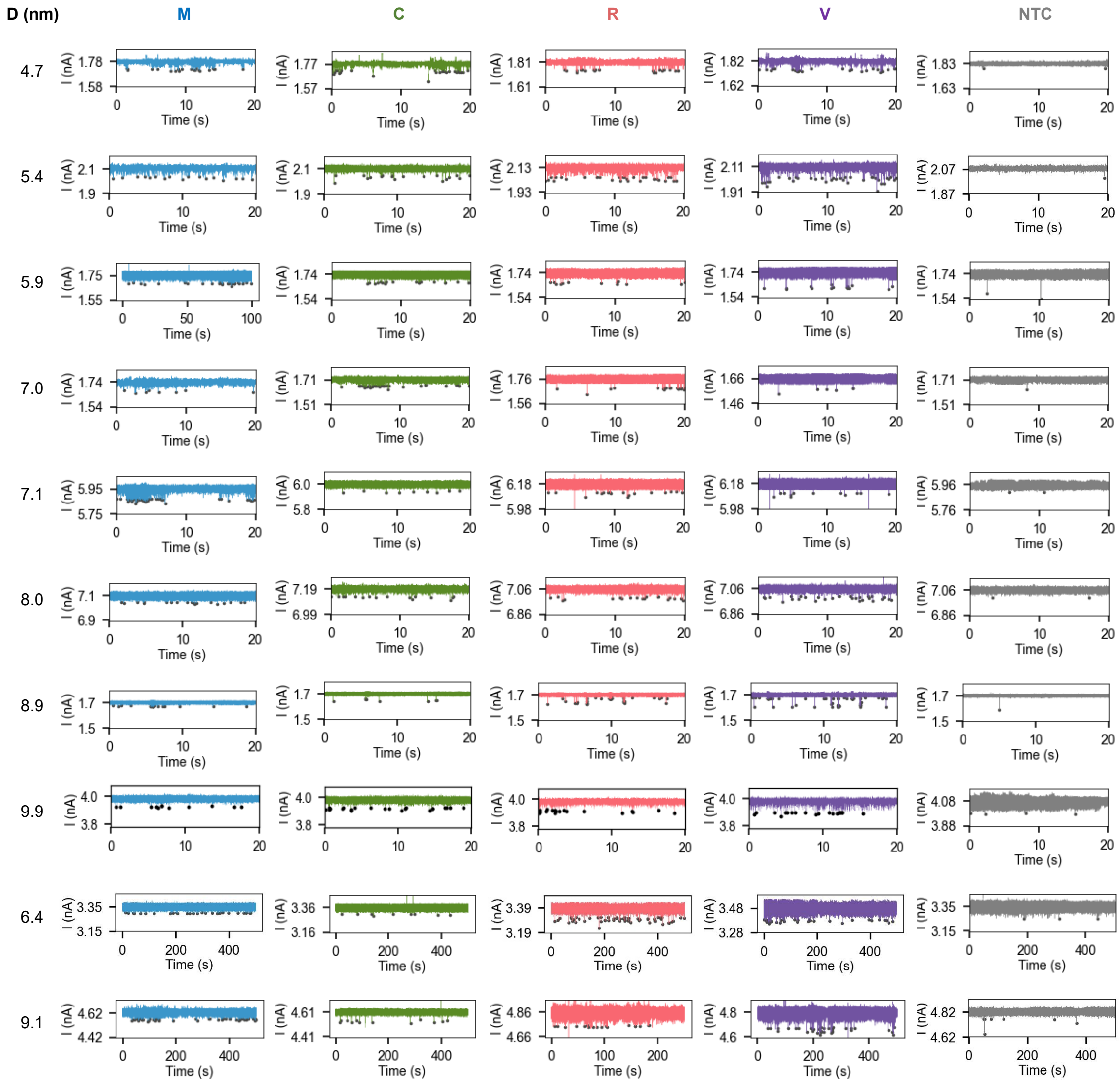

(Continue)

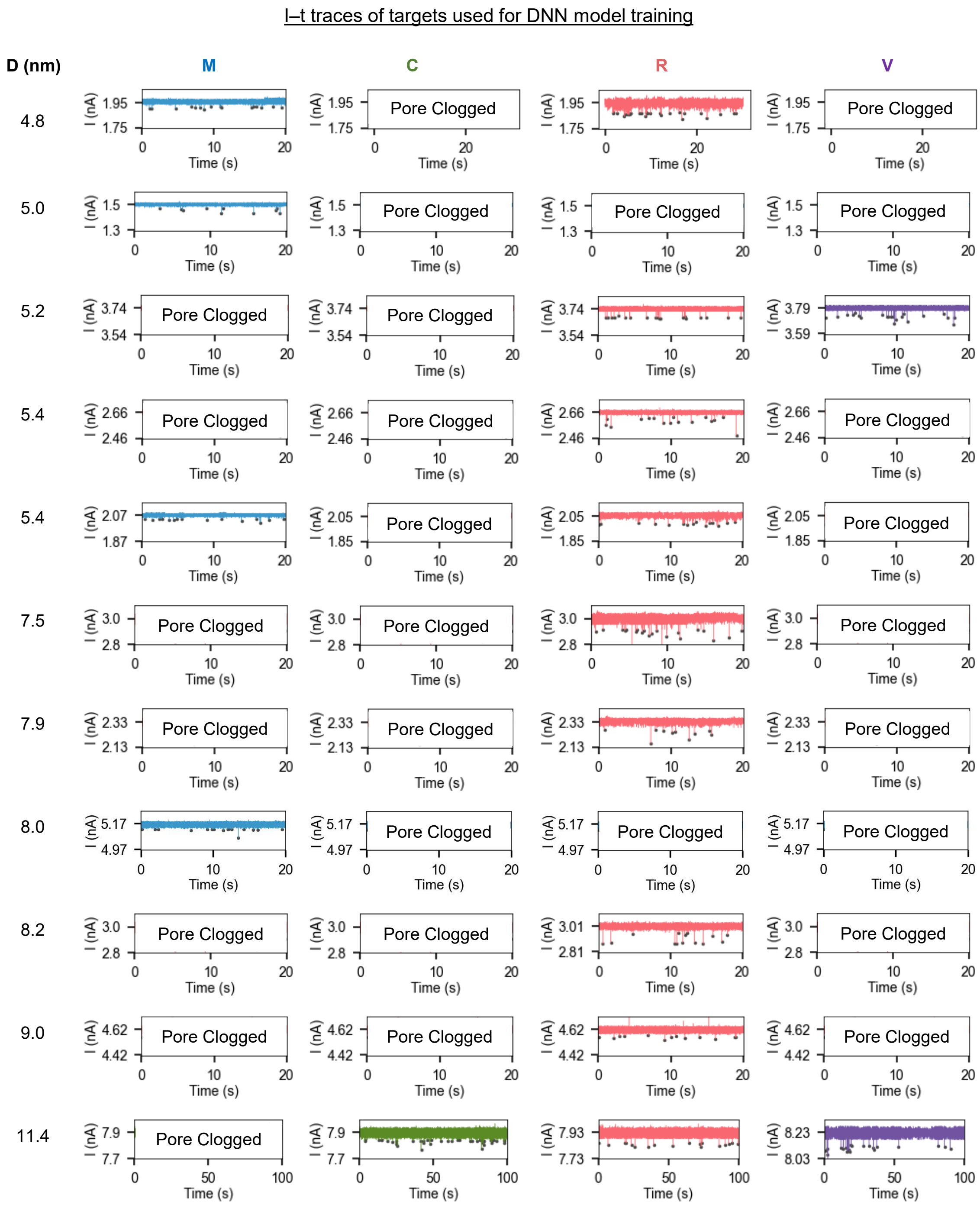

Figure S14. Current-time (I-t) traces of 66 samples used for training data from 21 nanopores. Representative acquisition windows are shown for each sample, with detected events marked by circular dots. Target classifications are indicated by color: blue for M, green for C, red for R, purple for V, and NTC. These traces illustrate the pore-to-pore variation in baseline current and event characteristics represented in the training dataset.

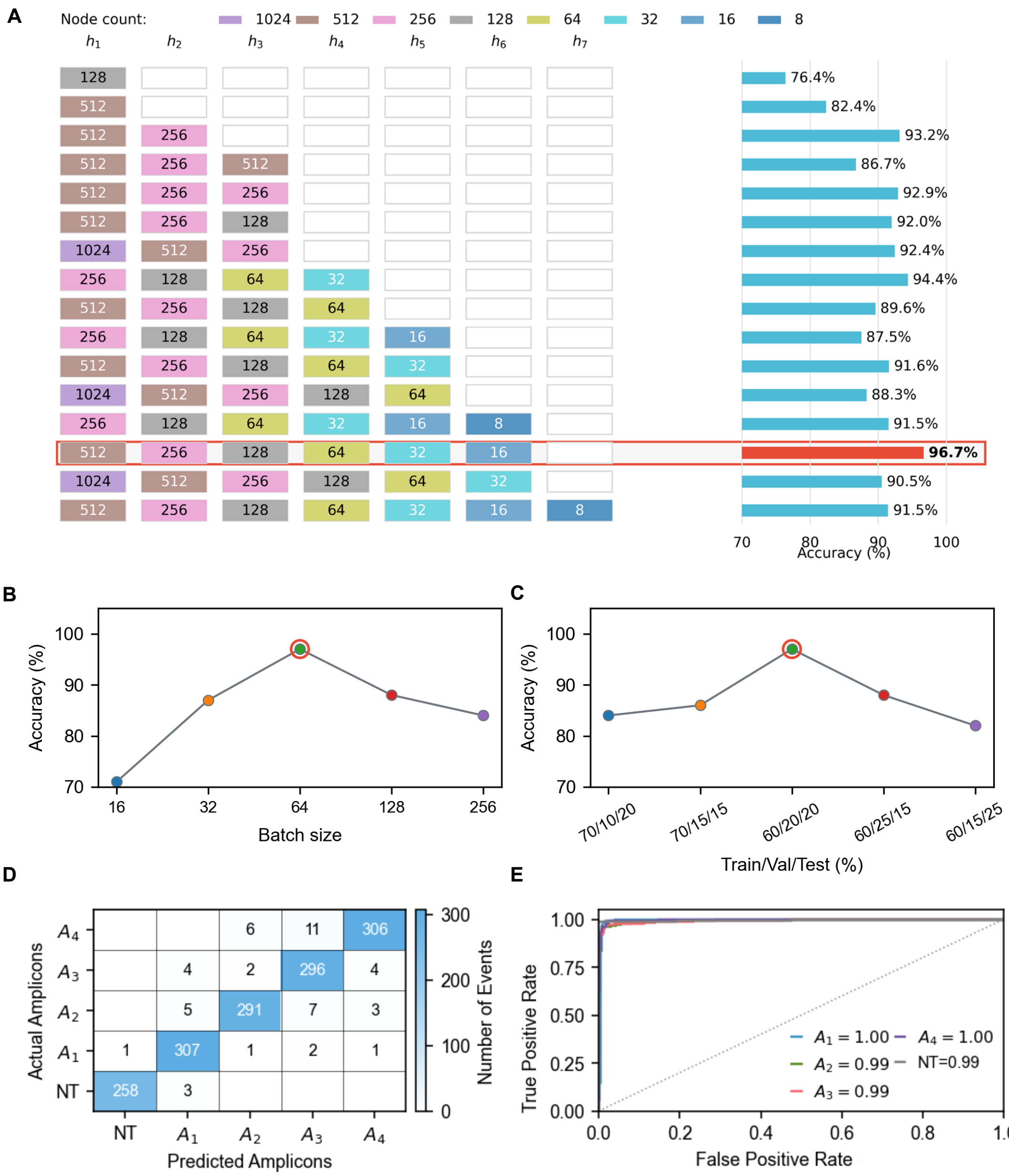

Figure S15. Optimization and performance of the DNN decoder for multiplexed amplicon classification. A) Sixteen fully connected network architectures spanning one to seven hidden layers and different node configurations were evaluated using the same six-feature input representation. Each colored cell indicates the number of nodes in the corresponding hidden layer, with the associated classification accuracy shown on the right. The 512–256–128–64–32–16 architecture achieved the highest accuracy of 96.7% and was selected for subsequent DNN training and evaluation**. B)** Test accuracy as a function of batch size, with a batch size of 64 yielding the highest accuracy of 97%. **C)** Test accuracy for different training, validation, and test splits, where a 60/20/20 split provides the best performance. **D)** Confusion matrix for five-class classification of A_1–A_4 and NT on the independent 23-sample held-out cohort, showing that most events are correctly assigned to their respective classes. **E)** Receiver operating characteristic (RO**C)** curves for each class, with AUC values of 0.99 to 1.00, confirming excellent discrimination among the four amplicons and NT.

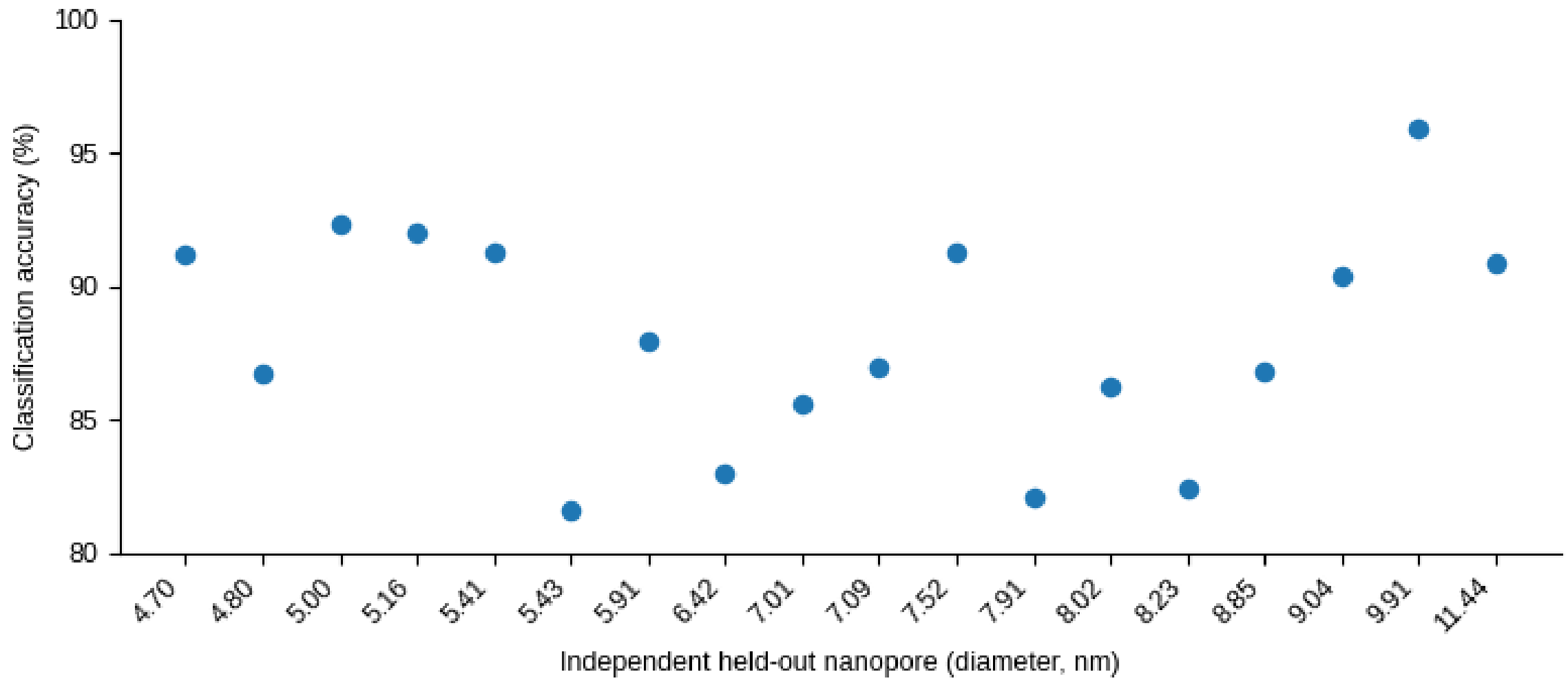

Figure S15F. Leave-one-pore-out validation. Each point represents the classification accuracy obtained when all events from one nanopore were excluded from model training, and that pore was subsequently used only for testing. The x-axis identifies the leave-out pore by its diameter.

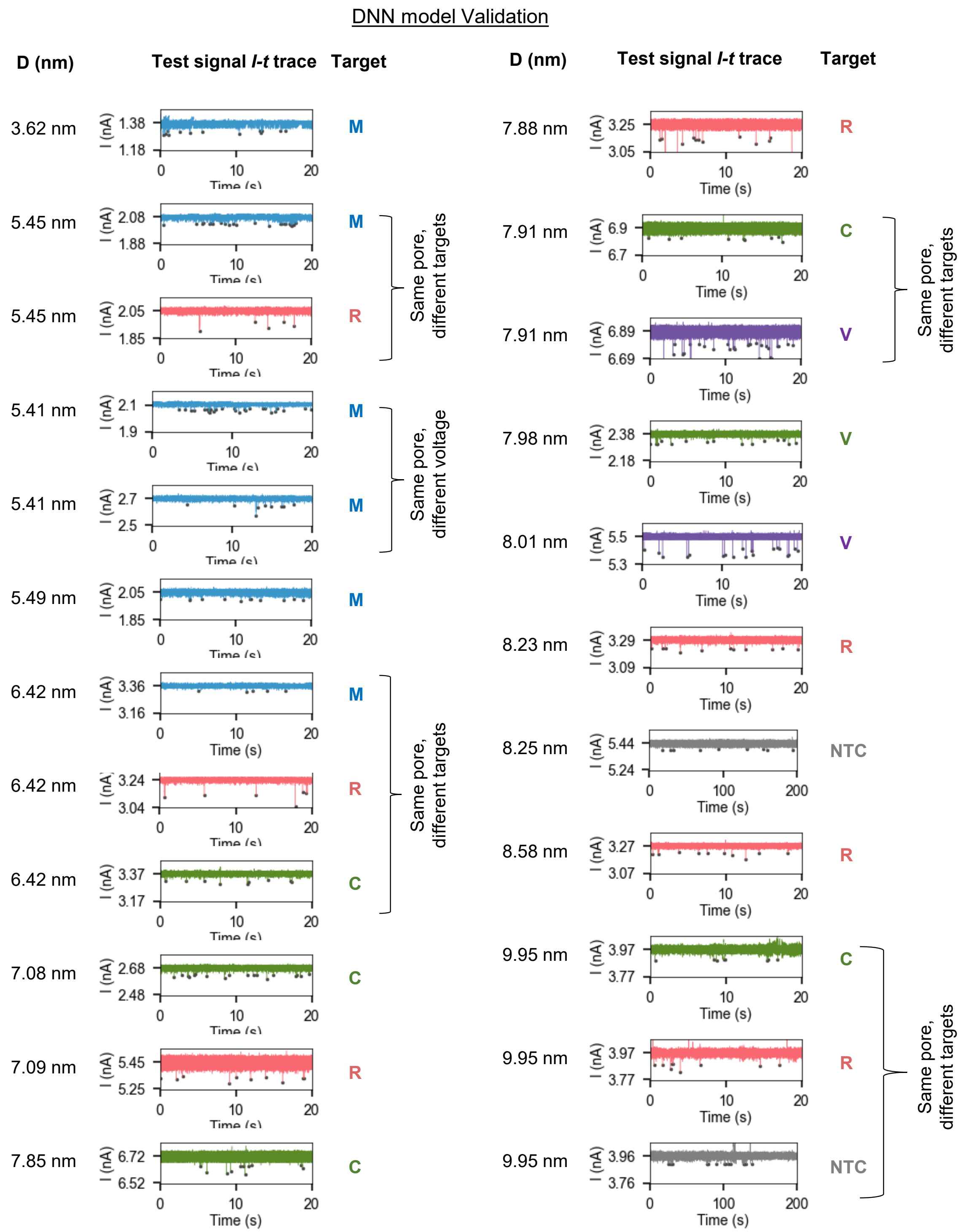

Figure S16. Current-time (I-t) traces of 23 samples used for held-out testing data from 16 nanopores. The traces are shown individually to illustrate the pore-to-pore variation in baseline current and event characteristics across the held-out pores, which would be obscured in a pooled summary. Representative acquisition windows are shown for each sample, with detected events marked by circular dots. Target classifications are indicated by color: blue for M, green for C, red for R, purple for V, and NTC.

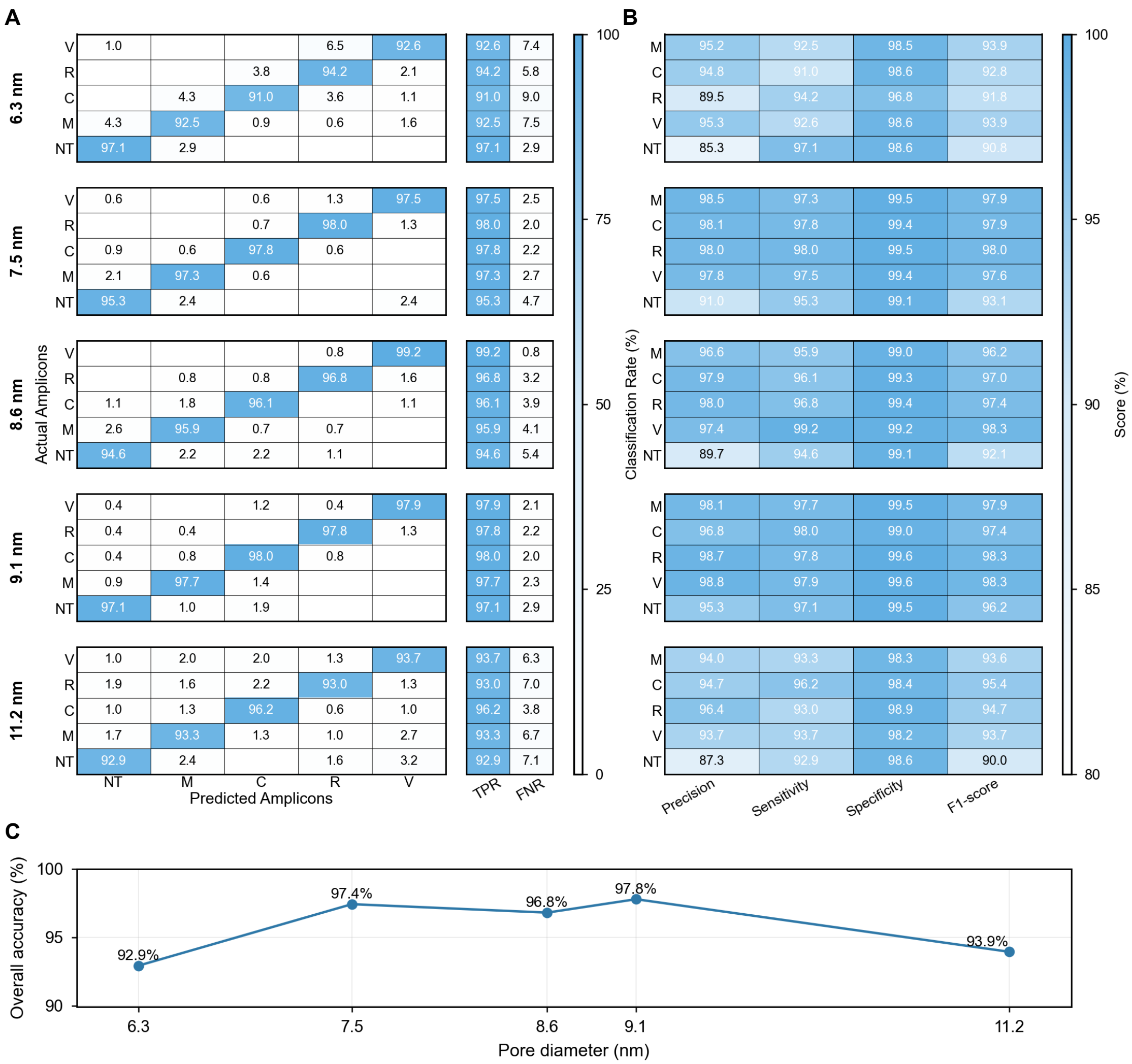

Figure S17. Nanopore-diameter-stratified performance of the DNN event classifier. A) Row-normalized confusion matrix for five-class event classification using nanopores with diameters of 6.3, 7.5, 8.6, 9.1, and 11.2 nm. Rows indicate the actual event class and columns indicate the DNN-predicted class for Mpox (M; A_1), Cowpox (C; A_2), RNase P (R; A_3), Vaccinia (V; A_4), and non-target events (NT). Values represent the percentage of events within each actual class. The adjacent columns report the class-specific true-positive rate (TPR) and false-negative rate (FNR). **B)** Per-class precision, sensitivity, specificity, and F1 score calculated separately for each nanopore diameter. **C)** Overall event-level classification accuracy as a function of nanopore diameter. Within the selected 6.3–9.1 nm operating window, the analysis included 4,904 test events, comprising 1,303, 1,400, 1,160, and 1,041 events at 6.3, 7.5, 8.6, and 9.1 nm, respectively. The corresponding overall accuracies were 92.9%, 97.4%, 96.8%, and 97.8%. An additional larger-pore comparison at 11.2 nm yielded an overall accuracy of 93.9%. These results demonstrate robust event classification within the selected operating window, with modest reductions at the 6.3 nm lower boundary and at the larger 11.2 nm pore size. Pores below 6.3 nm were not included in the analysis because frequent clogging, particularly for the longer amplicons, prevented reliable acquisition of a complete five-class evaluation dataset.

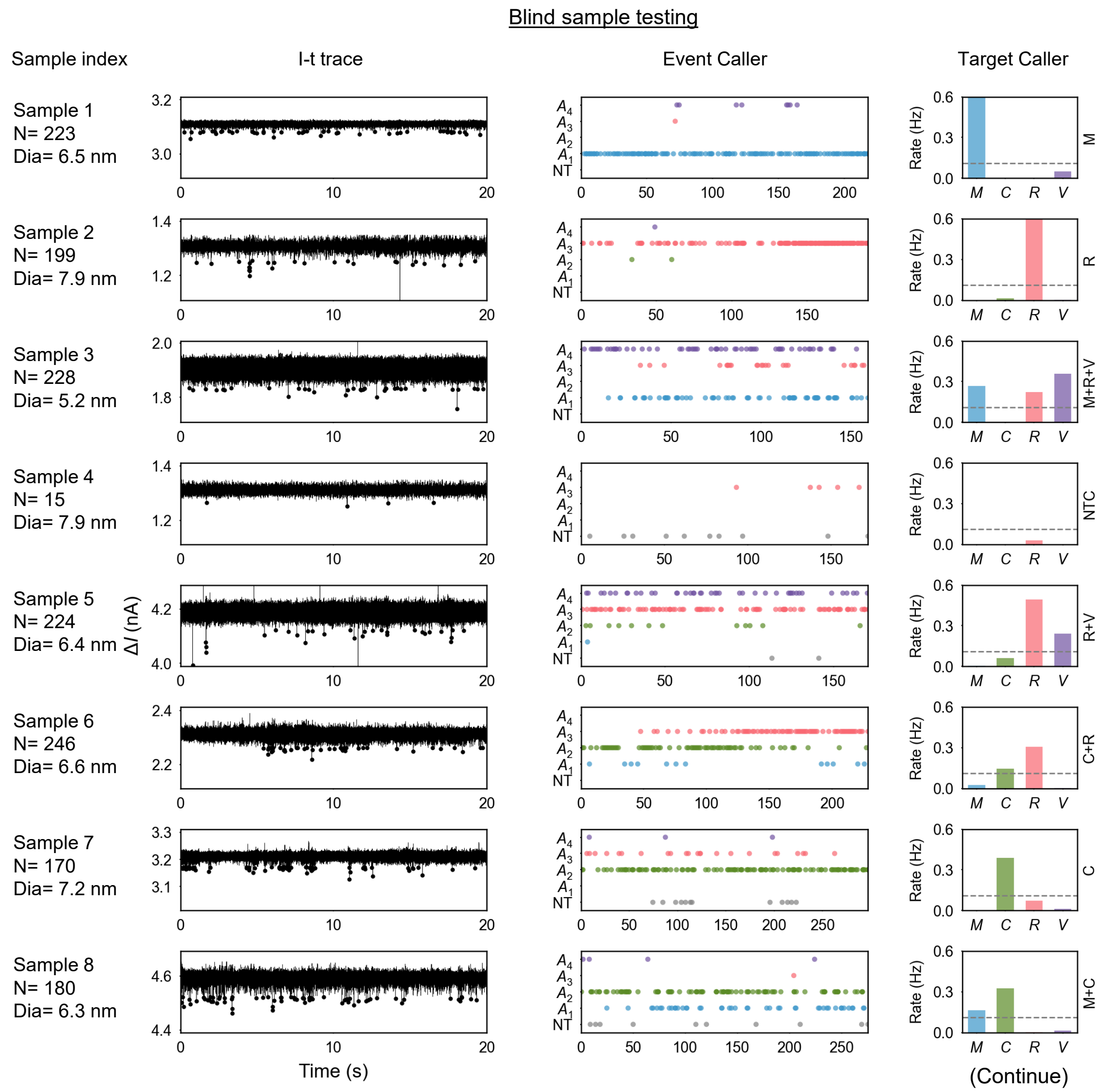

227 (Continue)

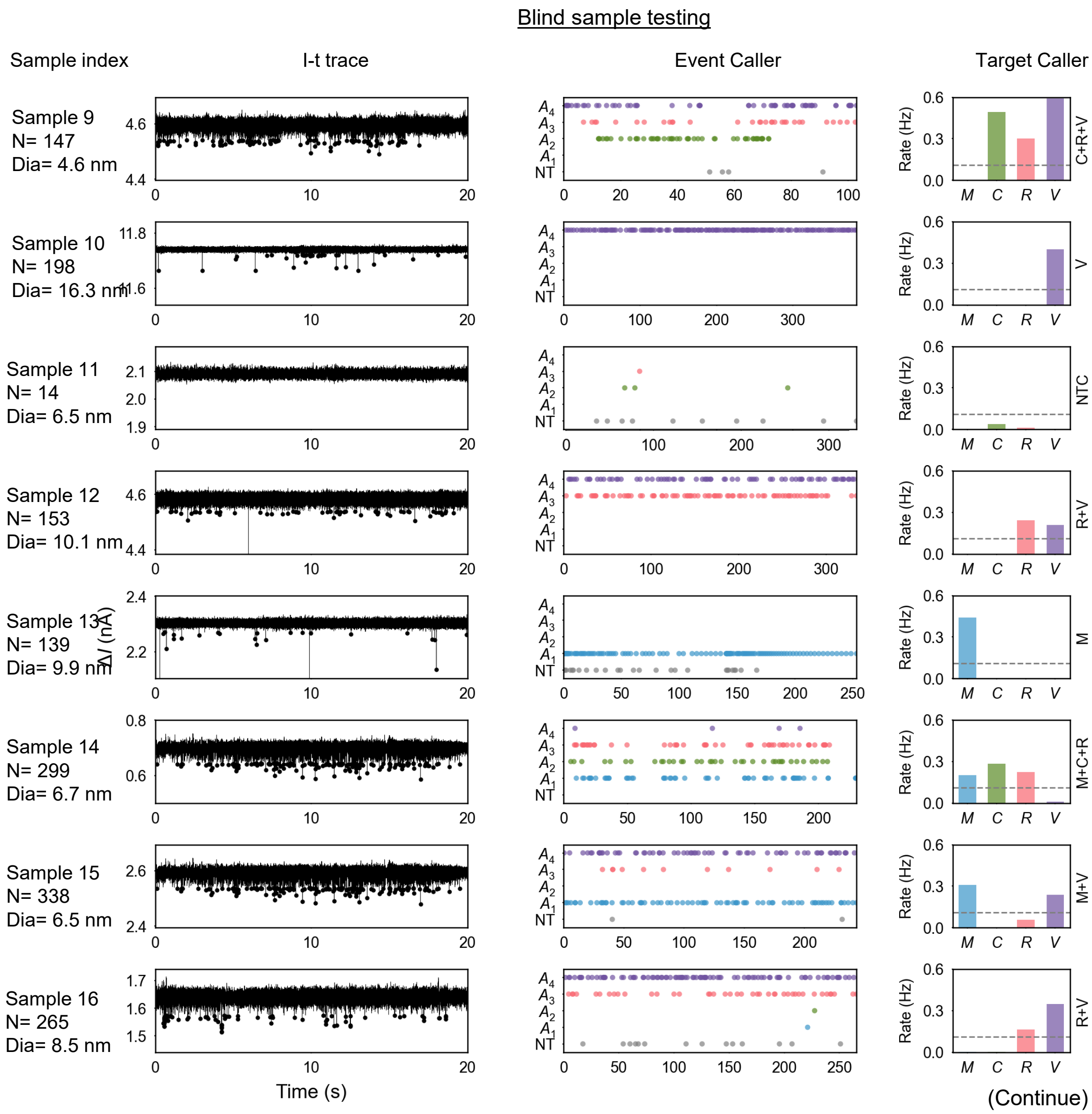
Blind sample testing
Sample index
I-t trace
Event Caller
Target Caller
Sample 9
N= 147
Dia= 4.6 nm
C+R+V
Sample 10
N= 198
Dia= 16.3 nm
V
Sample 11
N= 14
Dia= 6.5 nm
NTC
Sample 12
N= 153
Dia= 10.1 nm
R+V
Sample 13
N= 139
Dia= 9.9 nm
M
Sample 14
N= 299
Dia= 6.7 nm
M+C+R
Sample 15
N= 338
Dia= 6.5 nm
M+V
Sample 16
N= 265
Dia= 8.5 nm
R+V
ΔI (nA)
Time (s)
Rate (Hz)
M C R V
(Continue)

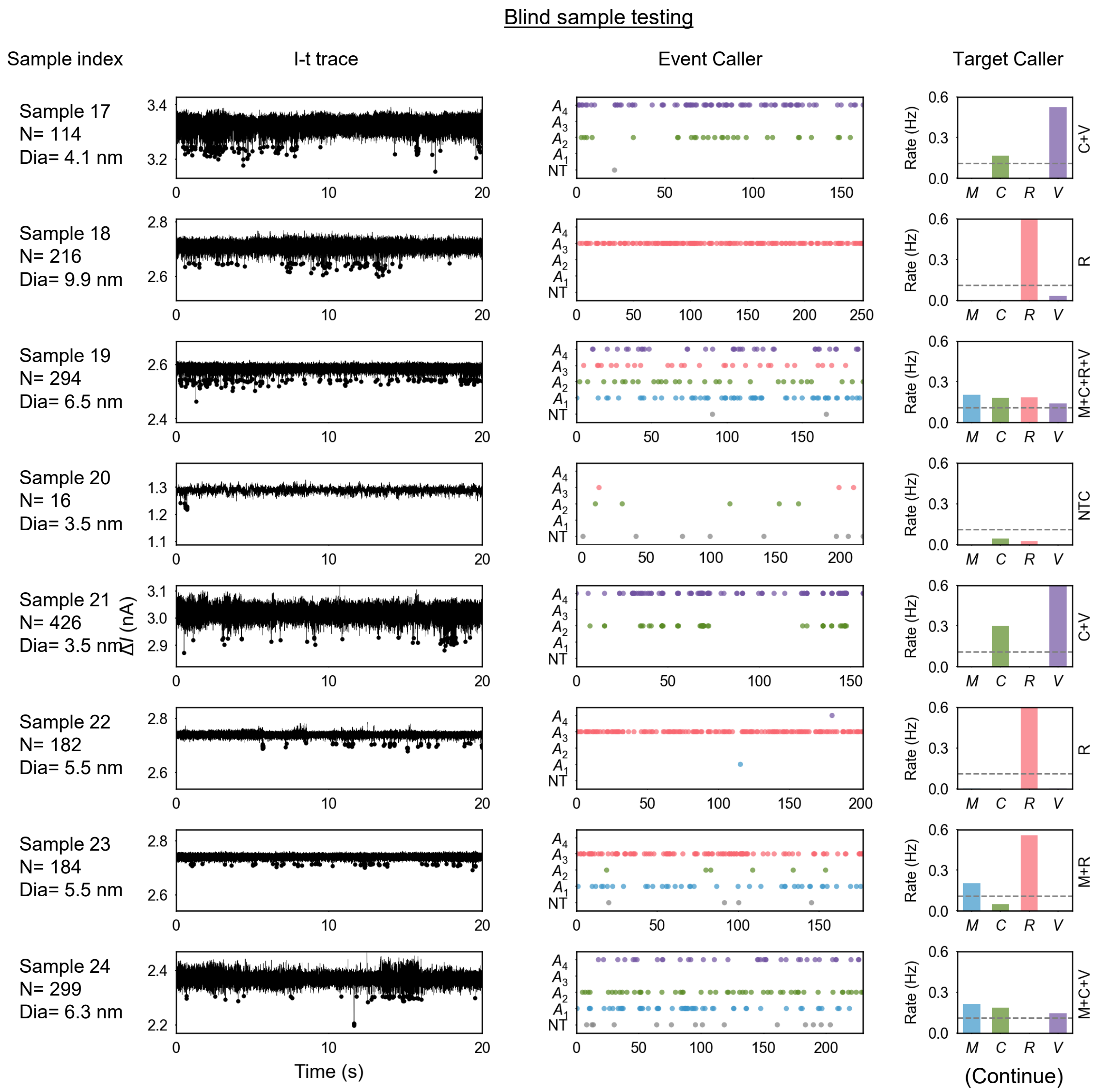
Blind sample testing
Sample index
I-t trace
Event Caller
Target Caller
Sample 17
N= 114
Dia= 4.1 nm
C+V
Sample 18
N= 216
Dia= 9.9 nm
R
Sample 19
N= 294
Dia= 6.5 nm
M+C+R+V
Sample 20
N= 16
Dia= 3.5 nm
NTC
Sample 21
N= 426
Dia= 3.5 nm
C+V
Sample 22
N= 182
Dia= 5.5 nm
R
Sample 23
N= 184
Dia= 5.5 nm
M+R
Sample 24
N= 299
Dia= 6.3 nm
M+C+V
ΔI (nA)
Time (s)
Rate (Hz)
M C R V
NT A_1 A_2 A_3 A_4
(Continue)

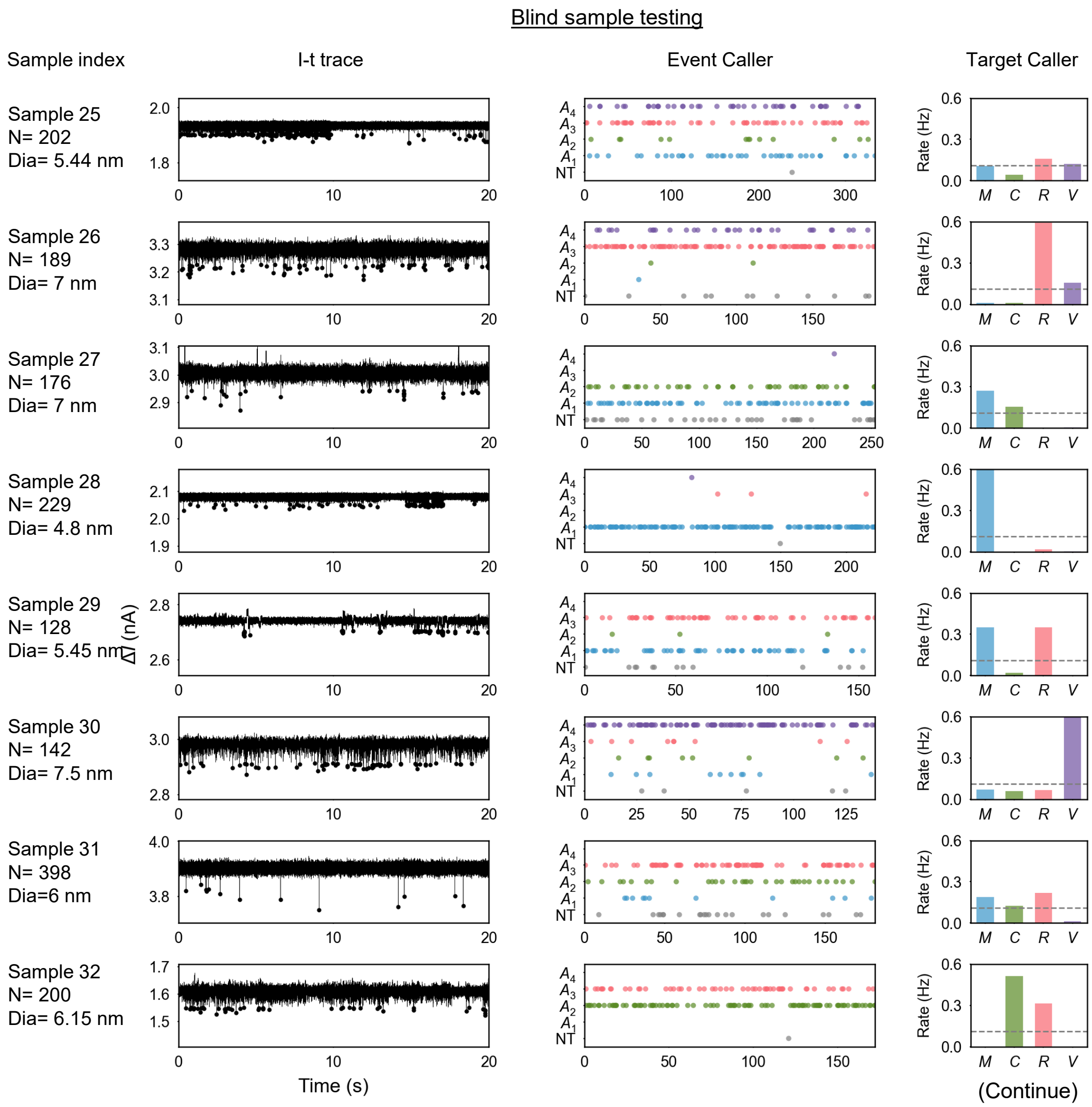
Blind sample testing
Sample index
I-t trace
Event Caller
Target Caller
Sample 25
N= 202
Dia= 5.44 nm
Sample 26
N= 189
Dia= 7 nm
Sample 27
N= 176
Dia= 7 nm
Sample 28
N= 229
Dia= 4.8 nm
Sample 29
N= 128
Dia= 5.45 nm
Sample 30
N= 142
Dia= 7.5 nm
Sample 31
N= 398
Dia=6 nm
Sample 32
N= 200
Dia= 6.15 nm
ΔI (nA)
Time (s)
A_4
A_3
A_2
A_1
NT
Rate (Hz)
M C R V
R+V
R+V
M+C
M
M+R
V
M+C+R
C+R
(Continue)

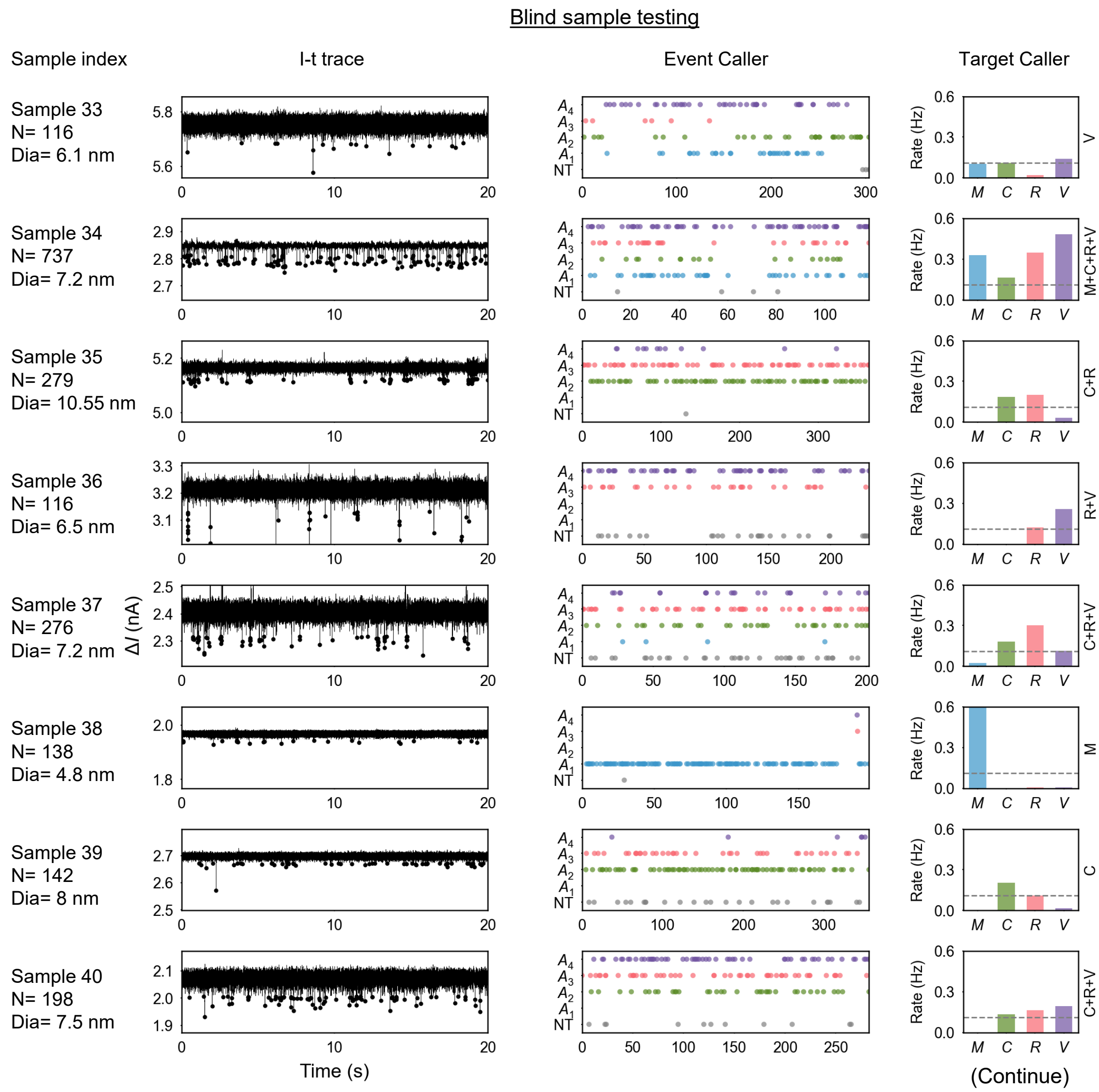
Blind sample testing
Sample index
I-t trace
Event Caller
Target Caller
Sample 33
N= 116
Dia= 6.1 nm
Sample 34
N= 737
Dia= 7.2 nm
Sample 35
N= 279
Dia= 10.55 nm
Sample 36
N= 116
Dia= 6.5 nm
Sample 37
N= 276
Dia= 7.2 nm
Sample 38
N= 138
Dia= 4.8 nm
Sample 39
N= 142
Dia= 8 nm
Sample 40
N= 198
Dia= 7.5 nm
ΔI (nA)
Time (s)
Rate (Hz)
V
M+C+R+V
C+R
R+V
C+R+V
M
C
C+R+V
(Continue)

231

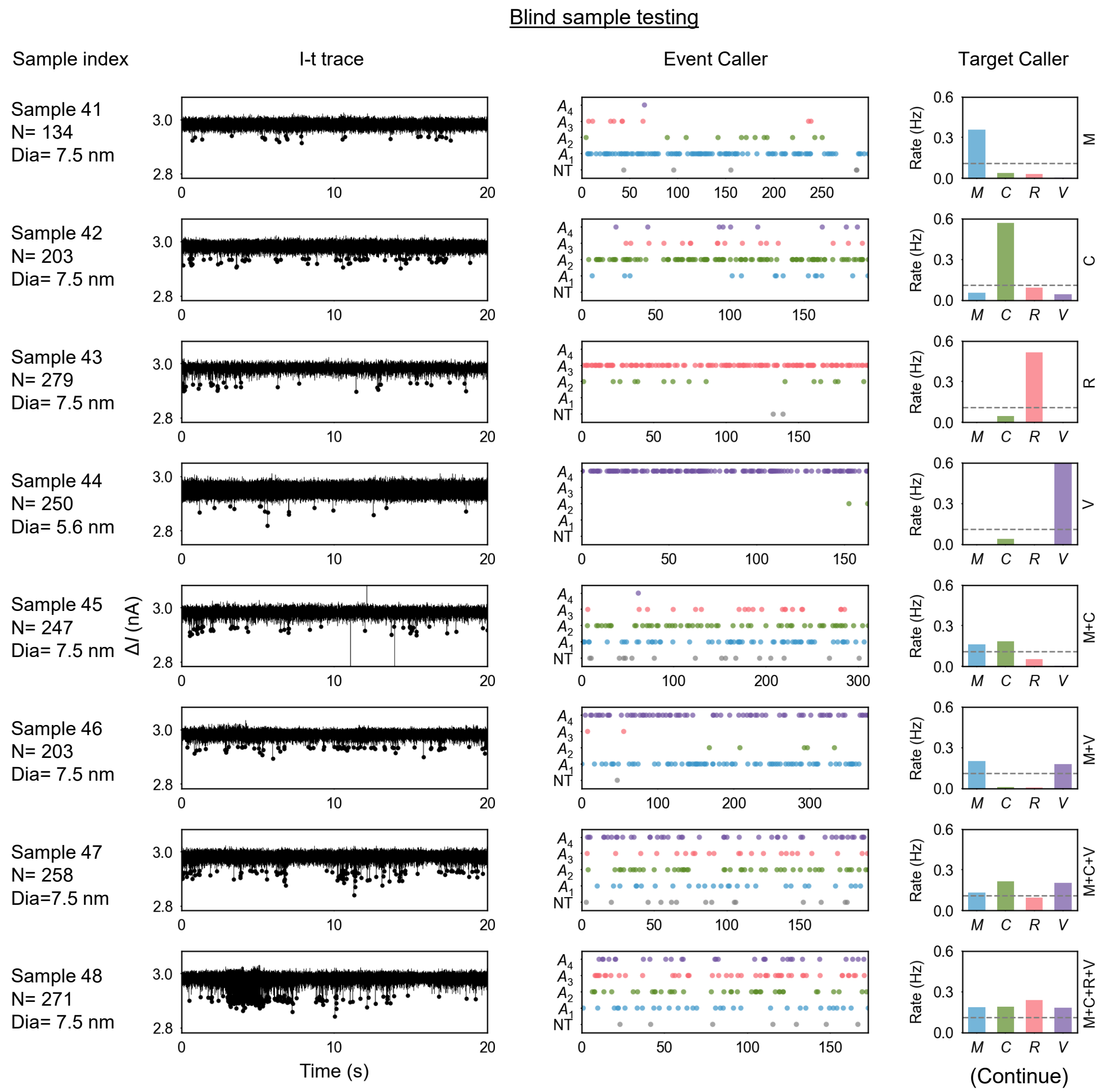
Blind sample testing
Sample index
I-t trace
Event Caller
Target Caller
Sample 41
N= 134
Dia= 7.5 nm
Sample 42
N= 203
Dia= 7.5 nm
Sample 43
N= 279
Dia= 7.5 nm
Sample 44
N= 250
Dia= 5.6 nm
Sample 45
N= 247
Dia= 7.5 nm
Sample 46
N= 203
Dia= 7.5 nm
Sample 47
N= 258
Dia=7.5 nm
Sample 48
N= 271
Dia= 7.5 nm
ΔI (nA)
Time (s)
Rate (Hz)
M
C
R
V
M+C
M+V
M+C+V
M+C+R+V
(Continue)

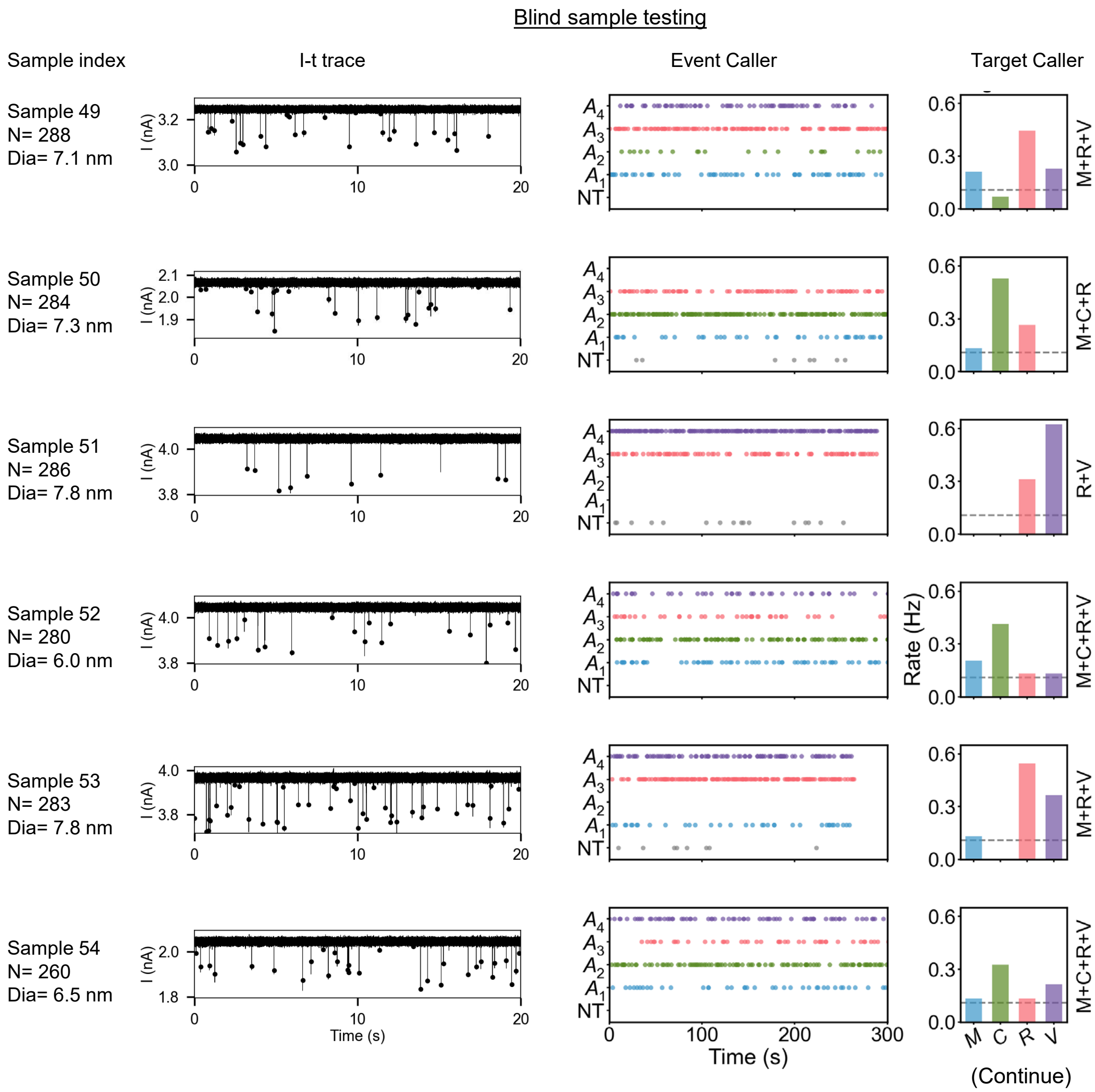

Blind sample testing
Sample index
I-t trace
Event Caller
Target Caller
Sample 49
N= 288
Dia= 7.1 nm
M+R+V
Sample 50
N= 284
Dia= 7.3 nm
M+C+R
Sample 51
N= 286
Dia= 7.8 nm
R+V
Sample 52
N= 280
Dia= 6.0 nm
M+C+R+V
Sample 53
N= 283
Dia= 7.8 nm
M+R+V
Sample 54
N= 260
Dia= 6.5 nm
M+C+R+V
I (nA)
Time (s)
Rate (Hz)
A_4
A_3
A_2
A_1
NT
M
C
R
V
(Continue)

234

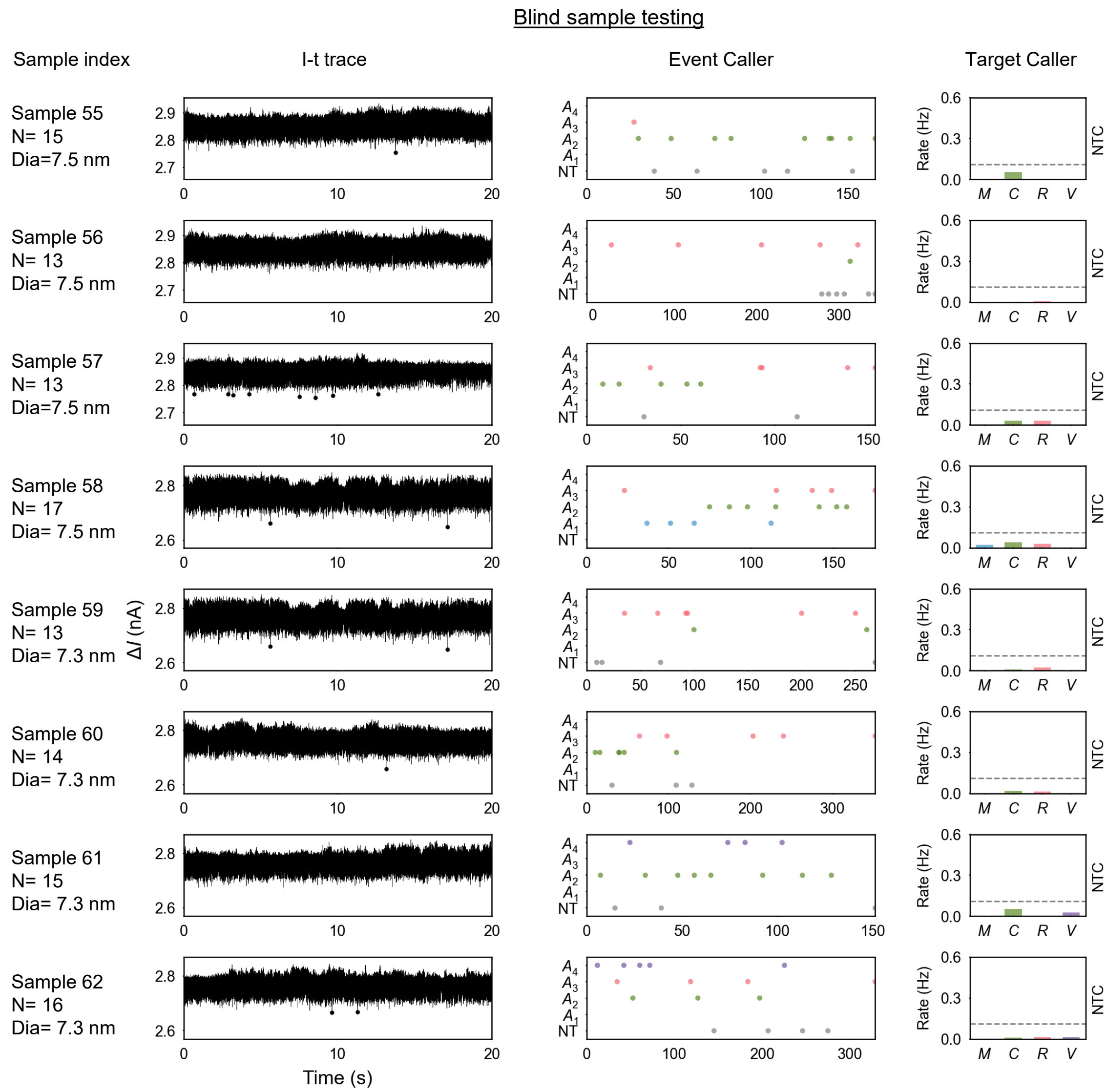

Figure S18. Sample-by-sample outputs for blinded multiplex calling across the 62-sample cohort. For each sample, the left panel shows the representative I-t trace, the middle panel shows time-stamped event-level classifications for the four target classes (A_1–A_4), and the NT class, and the right panel reports target-resolved event-rate histograms with the decision threshold indicated ($\mu + 3\sigma$ relative to the true no-template control baseline). The corresponding qualitative sample call is reported using the target symbols M, C, R, and V. Panels continue across pages to cover all 62 samples. Results are presented sample by sample so that the classification outcome underlying each blinded call summarized in **Figure 7B** can be inspected directly.

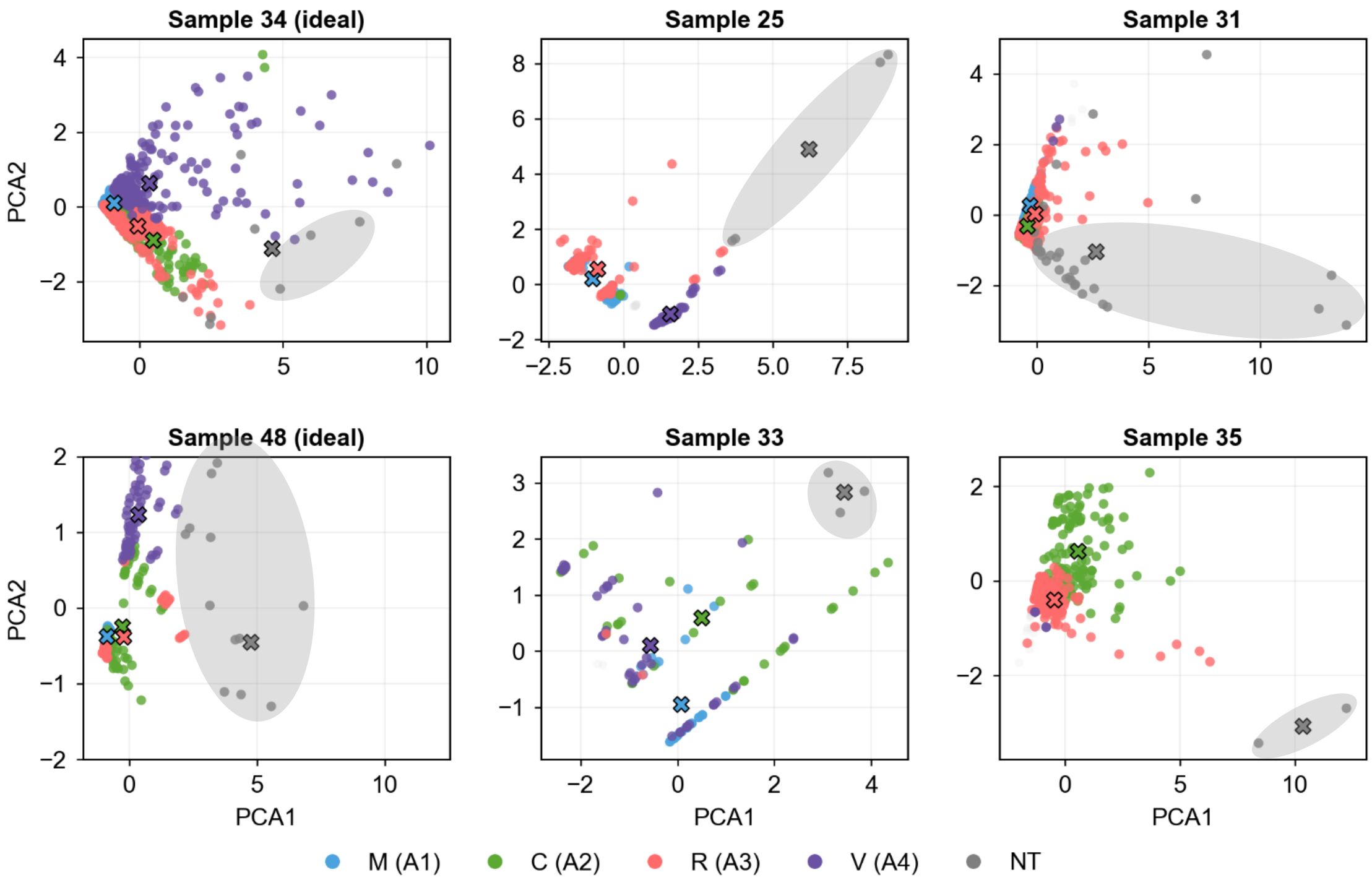

Figure S19. Event-level feature distributions of misclassified versus correctly classified blinded samples. Principal component analysis (PCA) of single-event translocation features for representative blinded samples. Events are colored by assigned molecular class: M (A_1), C (A_2), R (A_3), V (A_4), and non-target (NT). Black × markers indicate class centroids. Correctly classified reference samples #34 and #48 ("ideal") show dense target-event populations with clearer separation from NT events. In contrast, the misclassified samples #25, #31, #33, and #35 contain FN target populations, where target-consistent events are present but remain sparse or near the decision threshold. Samples #31 and #35 also contain FP calls, arising from low-abundance near-threshold background or nonspecific events assigned to an additional target class. Overall, these errors reflect threshold proximity, limited event abundance, and partial feature-space overlap rather than systematic cross-talk between target channels.

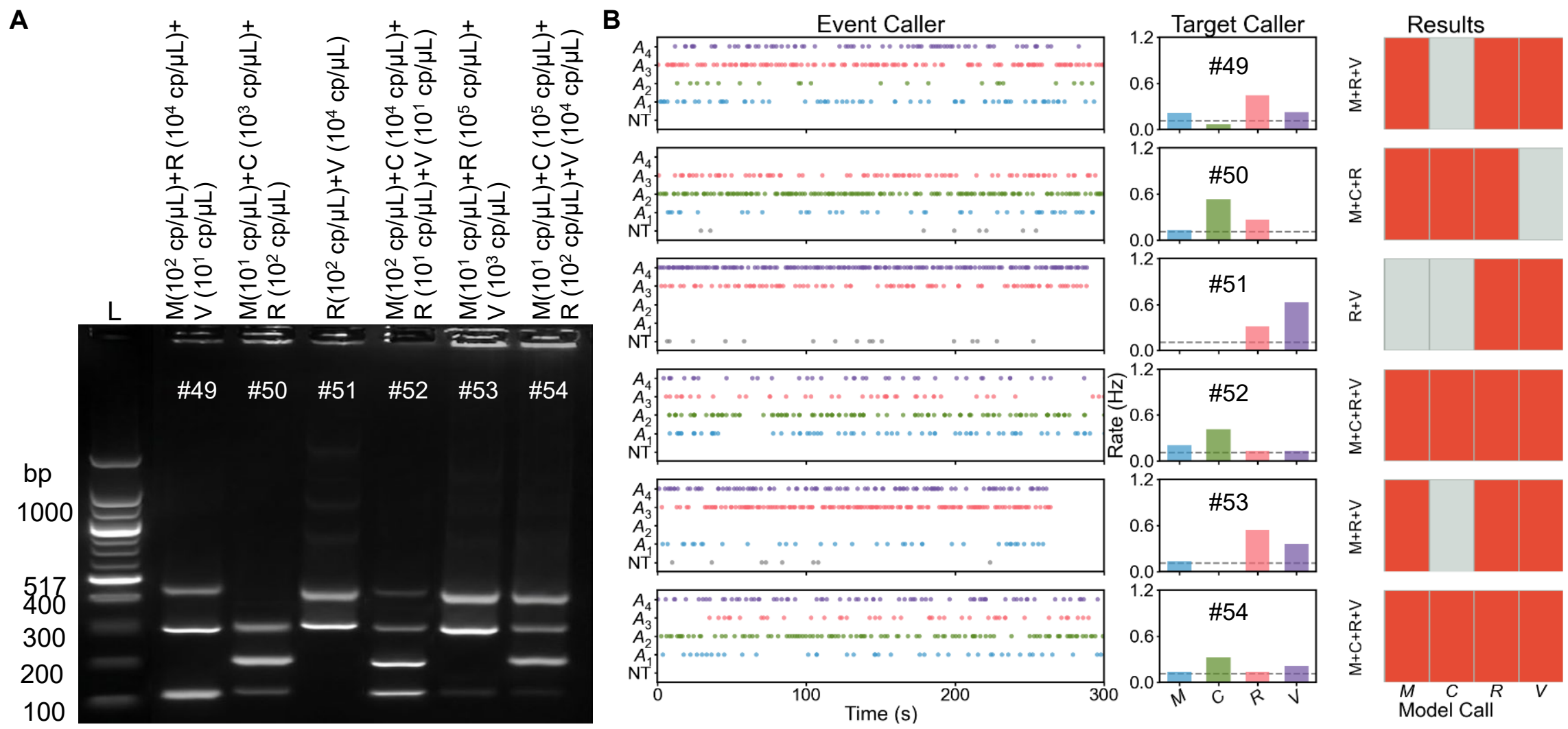

Figure S20. Detection under asymmetric target concentrations. Multi-target mixtures (samples #49–#54) combining high- and low-abundance targets at unequal inputs spanning ~10^1–10^5 copies/µL. **A)** Agarose gel confirms co-amplification of all intended targets; per-target input (M, C, R, V) is given above each lane. **B)** Nanopore analysis: the Event Caller assigns translocation events over 300 s to amplicon classes A_1–A_4 or non-target (NT); the Target Caller compares each per-target event rate (Hz) to an NTC threshold (dashed line), and the Results panel reports the model calls. Because each target-specific event rate is compared with the NTC-derived threshold, low-abundance targets stay above background, and model calls match every mixture's composition with no misclassification.

Section S3. Supplementary Tables

Table S1: Target sequences

Targets	Sequence
**Mpox**	TTTCGCGGGATACATCATCTATTATAGCATCAGCATCAGAATCTGTAGGCCGTGTATCAGCA<br>TCCATTGTCGTAGACCAACGAGGAGGAGTATCGTTGGAGCTGTAAACCATAGCA
**Cowpox**	GATGTGGCTGAACAGATACCTGCTACCATTGTCATCTTTAAAACTCTCCACTTTTCATCTTTT<br>CGGAGATCTTTCTTCAATGGTAGGATTATAATATCTGTGTTATAATCGTAATATCCACAATC<br>AGGATCTGTAAAGCGAGCTGAATCAGTATCCGTATCTGAATGCGGTTCAGCAGTCCCACCT<br>ATAAGGTATATATAATCATTTTACAACGGCAAGTCCAGCATTGCTCTGGGATATACAGTAG<br>A
**RNase P**	AGTCTGAGGCTCGTCCTGAATGATATGCGGCCTCGGACTTCAGCATGGCGGTGTTTGCAGAT<br>TTGGACCTGCGAGCGGGTTCTGACCTGAAGGCTCTGCGCGGACTTGTGGAGACAGCCGCTC<br>ACCGTGAGTTGCGGTCTCCCAGAGTCTCTGGGATGTCCCTGGAGGCTGATGGCGCGTGATCT<br>TACGGCATTATACGTATGATCGGTCCACGATCAGCTAGATTATCTAGTCAGCTTGATGTCAT<br>AGCTGTTTCCTGAGGCTCAATACTGACCATTTAAATCATACCTGACCTCCATAGCAGAAAGT
**Vaccinia**	GAAATAATACAAATGACTACGTTGTTATGAGTGCTTGGTATAAGGAGCCCAATTCCATTATT<br>CTTTTAGCTGCTAAAAGCGACGTCTTGTATTTTGATAATTATACCAAGGATAAAATATCTTA<br>CGACTCTCCATACGATGATCTAGTTACAACTATCACAATTAAATCATTGACTGCTAGAGATG<br>CCGGTACTTATGTATGTGCATTCTTTATGACATCAACTACAAATGACACTGATAAAGTAGAT<br>TATGAAGAATACTCCACAGAGTTGATTGTAAATACAGATAGTGAATCGACTATAGACATAA<br>TACTATCTGGATCTACACATTCACCGGAAACTAGTTCTAAGAAACCTGATTATATAGATAAT<br>TCTAATTGCTCGTCGGTATTCGAAATCGCGACTCCGGAACCAATTACTGATAATGTAGAAG<br>ATCATACAGACACCGTCACATACACTAGTGATAGC

Component	Volume (μL)/rxn	Final Concentration
Forward Primer (F_1 or F_2)	1.250	500 nM
Reverse Primer (R_1 or R_2)	1.250	500 nM
2x reaction RPA buffer	12.50	1 X
10x Basic E-mix (commercial)	2.50	1 X
dNTPs mix (commercial)	2.00	800~2000 μM
Nuclease-free water	2.00	
20x Core Reaction Mix (commercial)	1.25	1 X
Mpox/Cowpox/ RNase P/ Vaccinia target (2×10^5 cp/μL)	1.00	
Master mixture	Total vol= 23.75 μL	
Last step: Magnesium Acetate	1.25	14 mM
Incubate at 39°C for 20 min	Total vol= 25 μL	

Component	Volume (µL)/rxn	Final Concentration
Forward Primer (F_1)	1.000	400~500 nM
Forward Primer (F_2)	1.000	400~500 nM
Reverse Primer (R_1)	1.000	400~500 nM
Reverse Primer (R_2)	1.000	400~500 nM
2x reaction RPA buffer	12.50	1 X
10x Basic E-mix (commercial)	2.50	1 X
dNTPs mix (commercial)	1.50	1000~2000 µM
20x Core Reaction Mix (commercial)	1.25	1~1.2 X
Mpox target (stock: 1 × 10^5 cp/µL)	1.00	
Cowpox target (stock: 1 × 10^5 cp/µL)	1.00	
Master mixture	Total vol= 23.75 µL	
Last step: Magnesium Acetate	1.25	14 mM
Incubate at 39°C for 20 min	Total vol= 25 µL	

Table S4: Recipe of triplex RPA reaction

Component	Volume (µL)/rxn	Final Concentration
Forward Primer (F_1)	0.500	400~500 nM
Forward Primer (F_2)	0.500	400~500 nM
Forward Primer (F_4)	0.500	400~500 nM
Reverse Primer (R_1)	0.500	400~500 nM
Reverse Primer (R_2)	0.500	400~500 nM
Reverse Primer (R_4)	0.500	400~500 nM
2x reaction RPA buffer	12.50	1 X
10x Basic E-mix (commercial)	2.50	1 X
dNTPs mix (commercial)	1.50	1000~2200 µM
20x Core Reaction Mix (commercial)	1.25	1~1.4 X
Mpox target (stock: 1 × 10^5 cp/µL)	1.00	
Cowpox target (stock: 1 × 10^5 cp/µL)	1.00	
Vaccinia target (stock: 1 × 10^5 cp/µL)	1.00	
Master mixture	Total vol= 23.75 µL	
Last step: Magnesium Acetate	1.25	14 mM
Incubate at 39°C for 20 min	Total vol= 25 µL	

Table S5: Recipe of fourplex RPA reaction

Component	Volume (μL)/rxn	Final Concentration
Forward Primer (F_1)	0.500	400~500 nM
Forward Primer (F_2)	0.500	400~500 nM
Forward Primer (F_3)	0.500	400~500 nM
Forward Primer (F_4)	0.500	400~500 nM
Reverse Primer (R_1)	0.500	400~500 nM
Reverse Primer (R_2)	0.500	400~500 nM
Reverse Primer (R_3)	0.500	400~500 nM
Reverse Primer (R_4)	0.500	400~500 nM
2x reaction RPA buffer	12.50	1 X
10x Basic E-mix (commercial)	2.50	1 X
dNTPs mix (commercial)	1.50	1000~2400 μM
20x Core Reaction Mix (commercial)	1.25	1~1.6 X
Mpox target (stock: 2 × 10^5 cp/μL)	0.50	
Cowpox target (stock: 2 × 10^5 cp/μL)	0.50	
RNase P target (stock: 2 × 10^5 cp/μL)	0.50	
Vaccinia target (stock: 2 × 10^5 cp/μL)	0.50	
Master mixture	Total vol= 23.75 μL	
Last step: Magnesium Acetate	1.25	14 mM
Incubate at 39°C for 20 min	Total vol= 25 μL	

Component	Volume (μL)/rxn	Final Concentration
Forward Primer	1.20	600 nM
Reverse Primer	1.20	600 nM
TaqMan™ Fast Virus 1-Step Master Mix	10.00	1 X
Nuclease-free water	6.60	
Target (stock:1 × 10^5 cp/μL)	1.00	
Master mixture	Total vol= 20 μL	

Component	Volume (μL)/rxn	Final Concentration
Forward Primer (F_1)	2.00	400~600 nM
Forward Primer (F_2)	2.00	400~600 nM
Reverse Primer (R_1)	2.00	400~600 nM
Reverse Primer (R_2)	2.00	400~600 nM
TaqMan™ Fast Virus 1-Step Master Mix	10.00	1 X
Mpox target (stock: 1 × 10^5 cp/μL)	1.00	
Cowpox target (stock: 1 × 10^5 cp/μL)	1.00	
	Total vol= 20 μL	

Table S8: Recipe of triplex PCR reaction

Component	Volume (μL)/rxn	Final Concentration
Forward Primer (F_1)	1.00	400~600 nM
Forward Primer (F_2)	1.00	400~600 nM
Forward Primer (F_4)	1.00	400~600 nM
Reverse Primer (R_1)	1.00	400~600 nM
Reverse Primer (R_2)	1.00	400~600 nM
Reverse Primer (R_4)	1.00	400~600 nM
TaqMan™ Fast Virus 1-Step Master Mix	5.00	1 X
Nuclease-free water	6.00	1 X
Mpox target (stock: 1 × 10^5 cp/μL)	1.00	
Cowpox target (stock: 1 × 10^5 cp/μL)	1.00	
Vaccinia target (stock: 1 × 10^5 cp/μL)	1.00	

Total vol = 20 μL

Table S9: Recipe of four-plex PCR reaction

Component	Volume (μL)/rxn	Final Concentration
Forward Primer (F_1)	0.50	400~600 nM
Forward Primer (F_2)	0.50	400~600 nM
Forward Primer (F_3)	0.50	400~600 nM
Forward Primer (F_4)	0.50	400~600 nM
Reverse Primer (R_1)	0.50	400~600 nM
Reverse Primer (R_2)	0.50	400~600 nM
Reverse Primer (R_3)	0.50	400~600 nM
Reverse Primer (R_4)	0.50	400~600 nM
TaqMan Fast Virus 1-Step Master Mix (	5.00	1 X
Nuclease-free water	7.00	
Mpox target (stock: 2 × 10^5 cp/μL)	1.00	
Cowpox target (stock: 2 × 10^5 cp/μL)	1.00	
RNase P target (stock: 2 × 10^5 cp/μL)	1.00	
Vaccinia target (stock: 2 × 10^5 cp/μL)	1.00	

Total vol = 20 μL
